# Metformin’s Overall Effectiveness and Combined Action with Lifestyle Interventions in Preventing Type-2 Diabetes Mellitus in High-Risk Metformin-Naïve Patients: An Updated Systematic Review and Meta-Analysis of Published RCTs

**DOI:** 10.3390/jcm14144947

**Published:** 2025-07-12

**Authors:** Georgios I. Tsironikos, Vasiliki Tsolaki, George E. Zakynthinos, Vasiliki Rammou, Despoina Kyprianidou, Thomas Antonogiannis, Epaminondas Zakynthinos, Alexandra Bargiota

**Affiliations:** 1 Department of Research for General Medicine and Primary Health Care, Faculty of Medicine, University of Ioannina, Ioannina, University Campus, 45110 Ioannina, Greece; g.tsironikos@uoi.gr; 2 Department of Critical Care, University Hospital of Larissa, Faculty of Medicine, University of Thessaly, Mezourlo, 41335 Larissa, Greece; ezakynth@med.uth.gr; 3 3rd Department of Cardiology, “Sotiria” Chest Diseases Hospital, Medical School, National and Kapodistrian University of Athens, 11527 Athens, Greece; gzakynthinos@uth.gr; 4 Faculty of Medicine, University of Thessaly, Mezourlo, 41335 Larissa, Greece; vas19ram@gmail.com (V.R.); thomas.antonogiannis@gmail.com (T.A.); 5 Medical School, National and Kapodistrian University of Athens, 11527 Athens, Greece; depykyp@med.uoa.gr; 6 Department of Internal Medicine-Endocrinology, University Hospital of Larissa, Faculty of Medicine, University of Thessaly, Mezourlo, 41335 Larissa, Greece; abargio@med.uth.gr

**Keywords:** metformin, diet, nutrition, exercise, physical activity, lifestyle, diabetes

## Abstract

**Background:** The effectiveness of metformin in preventing Type-2 Diabetes Mellitus (T2DM) is examined. There are new available data. Currently, there are no available analyses classifying its effectiveness compared to placebo, standard care, or lifestyle interventions, and there is limited evidence on the combined action of metformin and lifestyle interventions in preventing T2DM. **Objective:** To calculate the updated overall effectiveness of metformin in preventing T2DM using all available and most recent data, and to explore the effectiveness of metformin and lifestyle interventions in preventing T2DM. **Materials and Methods:** A search was performed in PubMed and the Cochrane Library Central Register of Controlled Trials (CENTRAL) (from inception to 24 May 2025). A systematic review (SR) and meta-analysis (MA) of randomized controlled trials (RCTs) was carried out, including metformin-naïve adults with any identified diabetes risk factors. The overall effectiveness of metformin was estimated by combining studies that compare metformin against placebo, metformin and standard care against standard care, and metformin plus lifestyle interventions and the same lifestyle interventions. The combined action of metformin and lifestyle interventions was evaluated against standard care. We performed a GRADE assessment of the overall evidence. **Results:** Overall, metformin may reduce the incidence of T2DM by 23% in high-risk adults (OR 0.77, 95% CI 0.67, 0.88, *p*-value 0.0001) and 25% in patients with prediabetes (OR 0.75, 95%CI 0.66, 0.86, *p*-value < 0.0001). It is also effective in both obese and normal-weight patients, in Caucasians, in studies with female predominance, in studies with a mean age over 60 years, at 1700 mg daily, and after 18 months of administration. Effectiveness weakens after interruption of administration. Metformin is more effective compared to placebo and when combined with standard care than standard care alone, but not when combined with lifestyle interventions against lifestyle interventions alone. Metformin and lifestyle interventions reduce the incidence of diabetes in patients with prediabetes by 52% compared to standard care (OR 0.48, 95% CI 0.30, 0.77; *p*-value 0.002). There are effectiveness concerns in studies with more men than women, Asian Indians and Pakistanis, a mean age below 60 years, 500 mg of metformin daily, and after six months. The effect is reduced during post-intervention. Finally, metformin alone is more effective than standard care (OR 0.56, 95% CI 0.34, 0.90, *p*-value 0.02). The quality of evidence was moderate for the overall effectiveness of metformin and metformin combined with lifestyle interventions, and low for metformin against standard care. **Conclusions:** A 1700 mg dose of metformin daily is effective in preventing T2DM, especially in Caucasians, in women over 60 years, in prediabetes, and independent of obesity. Lifestyle interventions and 500 mg of metformin daily may prevent T2DM in patients with prediabetes, especially in men and Asian Indians or Pakistanis under 60 years. The effectiveness of complex interventions is more pronounced than that of metformin alone in patients with prediabetes. Further research is needed for post-intervention effectiveness, patients with any diabetes risk factors, patients from different regions, and women in complex interventions.

## 1. Introduction

Type-2 Diabetes Mellitus (T2DM) is the result of genetic predisposition, inflammation, and metabolic stress that lead to progressive pancreatic β-cell dysfunction with insufficient insulin secretion, often combined with insulin resistance and metabolic syndrome, resulting in hyperglycemia [1]. It is the most common type of DM, representing 90–95% of all diabetes cases [2]. The median estimated age of diabetes onset is 42.5 years [3]. The incidence of DM is distributed equally among females and males, affecting 8.2% of people across the world [3]. DM’s prevalence is increasing worldwide [4]. Globally, 642 million people will have T2DM by 2040 [5].

Currently, prediabetes, defined as either impaired fasting glucose (IFG), impaired glucose tolerance (IGT), or elevated hemoglobin A1c (HbA1c) between 5.7% and 6.4%, does not represent a separate metabolic disorder, constituting an independent risk factor for T2DM [1]. Additional risk factors include a history of gestational DM (GDM) [1], history of polycystic ovary syndrome (PCOS) [1], family history of DM in first-degree relatives [1], age greater than 45 years [6], high-risk ethnicities (i.e., Hispanic, Latino, Native American, African American, Asian American, Alaska Native, Pacific islander) [1,6], unhealthy nutrition [4], physical inactivity [1] or physical activity (PA) less than three times weekly [6], overweight or obesity [6], central adiposity [7], non-alcoholic fatty liver disease (NAFLD) [6], hypertension (HY) or anti-hypertensive treatment [1], hypertriglyceridemia (>250 mg/dL) and/or low high-density lipoprotein (HDL) cholesterol (<35 mg/dL) [1], cardiovascular disease (CVD) [1], insulin resistance-related acanthosis nigricans [1], and human immunodeficiency virus (HIV) infection [1].

Metformin is a cornerstone in T2DM treatment either alone [8] or combined with another antidiabetic drug [9], and its administration during pregnancy may decrease the incidence of GDM in women with PCOS [10,11,12].

For high-risk patients, two previously published meta-analyses (MAs), one in 2008 and the other in 2019, reported significant results for metformin in preventing T2DM [13,14]. For patients with prediabetes, metformin is also effective in preventing T2DM, according to four other MAs [15,16,17,18]. Two of them are published in the Chinese language [15,18], and the remaining two include data published before 2021. Finally, a MA published in 2023 included data published before 2016 and demonstrated a decreased incidence of T2DM in both high-risk and prediabetic patients [19].

Systematic reviews (SRs) and MAs have also declared the effectiveness of lifestyle interventions alone in preventing T2DM in high-risk [20] and prediabetic patients [21]. An antidiabetic regimen including metformin in addition to lifestyle modifications should be considered in real life but needs ongoing healthcare support [22].

At present, the overall effectiveness of metformin is not clearly classified in terms of placebo, standard care, and lifestyle interventions. The key clinical questions that we aim to address in the present MA are (1) which patient population could metformin benefit more, (2) what should be the optimal dose, (3) what should be the length of treatment, and (4) whether there is a post-intervention benefit. Moreover, we intend to assess (1) if metformin administration is more effective than standard care or lifestyle interventions, and (2) whether the combination of metformin and lifestyle interventions is more effective in preventing diabetes. Finally, the most recently published data have not been systematically analyzed, as they were not included in previous MAs [23,24,25].

## 2. Materials and Methods

Our initial protocol included a search for pharmaceutical and lifestyle interventions for diabetes prevention between 1 January 2000 and 31 July 2024 and was pre-registered in the Open Science Framework (OSF) (Registration DOI 10.17605/OSF.IO/8XH4G). To broaden the date of search (from inception to 24 May 2025) and specify the topic of the present study, we created an updated protocol in the OSF (Registration DOI 10.17605/OSF.IO/D4MRH [26]). The SR was performed according to PRISMA extension guidelines for complex interventions [27].

### 2.1. Search Strategy

The search for eligible RCTs was performed in PubMed and the Cochrane Library Central Register of Controlled Trials (CENTRAL) (from the inception of data to 24 May 2025). The keywords were related to diabetes, metformin, diet or nutrition, exercise or physical activity (PA), and lifestyle (Supplemental Table S1). The search in PubMed was applied combined with the Cochrane collaboration search algorithm for RCTs (Supplemental Table S1). The systematic search was completed by using the same keywords in CENTRAL. After removing duplicates using EndNote 21.5 software, four researchers (GIT, VT, GZ, VR) screened all databases. Potentially eligible studies based on titles and/or abstracts were retrieved, and the full text was checked. A fifth contributor (AB) checked the studies that the four investigators (GIT, VT, GZ, VR) could not decide on. Discrepancies were solved through consensus.

### 2.2. Eligibility Criteria

We accepted RCTs in the English language. Pilot or feasibility RCTs and studies reporting secondary analyses of RCTs were not accepted. Conference proceedings were also excluded. The PICO (population, intervention, control, outcome) approach was implemented to select trials. RCTs with adult participants who had any identified risk factor for T2DM that received metformin (alone or combined with standard care or lifestyle interventions) versus placebo, standard care, or lifestyle interventions, and reporting new occurrences of T2DM were considered eligible. Studies assessing the incidence of T2DM either as the primary or secondary outcome were considered eligible. RCTs that included patients who had previously received metformin and did not report new cases of T2DM in their outcomes were excluded. Overweight and obesity were also considered as risk factors. The definition of standard care was accepted as reported in each study. Any diagnostic modality for T2DM was accepted. If studies included more than one diagnostic test and reported different results, we accepted the laboratory exam with the most T2DM occurrences. For studies reporting the incidence of diabetes in different time periods, we accepted diagnoses in the final period of interventions.

The overall effect of metformin was evaluated in studies comparing metformin versus placebo, and in studies applying standard care or lifestyle intervention in the control group and metformin with the same standard care or lifestyle intervention in the experimental group.

### 2.3. Data Extraction

The data were extracted by four investigators (GIT, VT, GZ, VR). Where necessary, a fifth investigator (AB) contributed to the final decision. Data extraction included the name of the first author, publication year, country, type of RCT with number of clusters or centers if clustered or multicenter, number of arms if multiarm, study duration, and drop-out rate. We also extracted potential follow-up durations for studies assessing post-intervention outcomes. Data extraction also included the total sample size, characteristics of participants (gender, age, risk factors for T2DM), characteristics of interventions (dosage, duration), characteristics of the comparator arm, the number of patients that were analyzed for T2DM, events of T2DM, diagnostic tests for diabetes, and potential adverse events.

### 2.4. Quality Assessment of the Studies and Rating of Overall Evidence

Regarding the quality of eligible trials, the data were also extracted by four researchers (GIT, VT, GZ, VR). If required, a fifth researcher (AB) checked the items. The risk of bias tool proposed by the Cochrane Collaboration was used for quality assessment of eligible RCTs [28]. To rate overall evidence, we used the Grading of Recommendations, Assessment, Development, and Evaluation (GRADE) tool (GRADEpro, version 3.6.1 McMaster University, 2011).

### 2.5. Statistical Analysis

For our analyses, we used the Review Manager software version 5.4.1 (Cochrane Collaboration, London, UK) and the Statistical Package for the Social Sciences (SPSS) software version 29.0 (SPSS, Inc., Chicago, IL, USA).

The main analyses included all available data. The statistically significant level was generally set at *p*-value < 0.05 [26]; for Cochran’s Q statistic, it was set at *p*-value < 0.1 [26]. MAs were performed to combine the events of T2DM. Heterogeneity across studies was assessed by Cochran’s Q statistic (statistically significant for *p*-value < 0.1) [29] and measured by the I^2^ index (<25%, low; 25–49%, moderate; 50–75%, large; >75%, very large) [30,31]. Both fixed effects (FE) and random effects (RE) models of MA were created. If heterogeneity was large, synthesis was performed by the RE model [30,31].

Subgroup analyses were performed based on the studies’ characteristics (similar countries, follow-up durations), population characteristics (gender, mean age, diabetes risk factors), and intervention characteristics (dosage, duration, T2DM assessed as the primary or secondary outcome). Sensitivity analyses were also performed to assess the effect of the RCT with the largest sample size, studies with post-intervention follow-up of participants, and studies with drop-outs. Finally, we performed meta-regression analyses with study duration and baseline risk as covariates of the T2DM odds ratio (OR) [32].

Publication bias was assessed optically by funnel plots (a symmetrical inverted funnel in the absence of bias), and statistically by Egger’s test [33].

## 3. Results

**Figure 1:**
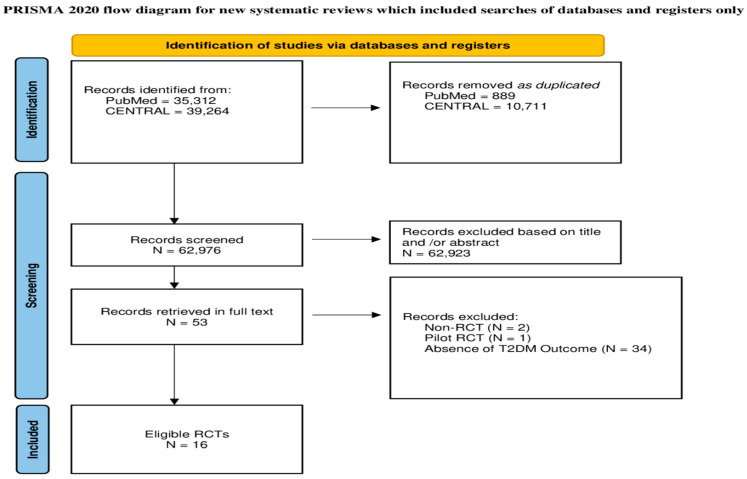
Flowchart of the study selection procedure.

The initial search yielded 74,576 items. After removing 11,600 duplicates, 62,976 items remained to be assessed for potential eligibility. A total of 62,923 papers were excluded based on their title and/or abstract. Then, we retrieved the full text of the remaining 53 papers. Two studies had non-RCT designs, one was a pilot RCT, and 34 did not report the outcome of T2DM and were excluded. Finally, 16 RCTs were accepted (Figure 1).

### 3.1. Characteristics of Eligible Studies

The eligible RCTs were published between 1996 and 2023, and they all had a parallel design [23,24,25,34,35,36,37,38,39,40,41,42,43,44,45,46]. Seven were conducted in Asia [23,24,35,38,39,40,43] (two in India [38,43], two in China [24,35], one in Thailand [23], and one in Pakistan [40]), six trials in Europe [34,36,41,42,45,46] (two in Scotland, United Kingdom (UK) [41,46]; two in The Netherlands [42,45]; one in France [34]; and one in Finland [36]), two in north America (in the United States of America (USA)) [37,44], and one in Africa (in Tanzania) [25]. Four studies were multiarm [37,38,40,44] (one study with four arms [38], and the other three with three arms [37,40,44]). Thirteen RCTs were single-centered [23,24,35,36,38,39,40,41,42,43,44,45,46], and three studies were multicentered [25,34,37] (one of them included 27 centers [37], while two did not report the number of included centers [25,34]). Study duration varied between 4 and 48 months [23,24,25,34,35,36,37,38,39,40,41,42,43,44,45,46]. Two studies reported participants’ post-intervention follow-up: a 30-month duration in one of them [43] and a 24-month duration in the other one [45]. Five trials had no drop-outs [23,36,38,42,45]. Six studies had a drop-out rate up to 10% (2% [44], 5% [24,43], 7% [46], 8% [41], 10% [37]), three between 10% and 20% (13% [39], 14% [40], 19% [25]), and two more than 20% (22% [25], 29% [34]) (Table 1).

### 3.2. Characteristics of Participants

A total of 8529 adults were included in eligible trials [23,24,25,34,35,36,37,38,39,40,41,42,43,44,45,46]. Male and female genders represented 54% and 46% of the studies’ participants [23,24,25,34,35,36,37,38,39,40,41,42,43,44,45,46]. Their mean age varied between 44.4 [43] and 67.5 years [37]. The ethnicities included 3515 Asians (41%) [1952 Chinese and Thailand Asians (56%), 1106 Indians (31%), 317 Pakistanis (9%), 124 Asian Americans including Pacific Islanders (4%), and 14 Asians living in Europe (<1%)], 3227 Caucasians (38%) [1769 (55%) from North America and 1458 from Europe (45%)], 1017 Africans (12%) [644 African Americans (63%), 364 Sub-Saharan Africans (36%), and 9 Africans living in Europe (<1%)], 600 Hispanics (7%), and 171 Indian Americans (2%) [23,24,25,35,36,37,38,39,40,41,42,43,44,45,46]. One study did not report data about ethnicities [34] (Table 2).

#### Risk Factors

Several T2DM risk factors were evaluated in eligible trials [23,24,25,35,36,37,38,39,40,41,42,43,44,45,46]. Probable concurrence of risk factors that may affect a portion of individuals was reported in eight RCTs [23,25,33,34,35,37,38,46]. Prediabetes was the most common overall risk factor, considered in eleven RCTs [23,24,25,35,36,37,38,39,40,43,44], overweight/obesity in four trials [34,36,37,43], CVD in four RCTs [42,43,45,46], central adiposity in three studies [34,41,43], HIV infection in another two [23,25], and family history of T2DM in first-degree relatives in one study [36]. The overall number of T2DM risk factors included was one in seven studies [24,35,38,40,42,45,46], two in five studies [23,25,34,37,41], and three in two studies [36,43]. Probable coexistent risk factors were included in eight studies, comprising prediabetes, family history of T2DM or GDM, HY, elevated triglycerides, and low HDL cholesterol [23,25,34,35,37,38,43,46] (Table 2).

### 3.3. Characteristics of Interventions and Comparators

The components of comparator arms vary across studies [23,24,25,35,36,37,38,39,40,41,42,43,44,45,46]. They include either single or combined elements [23,24,25,35,36,37,38,39,40,41,42,43,44,45,46]. Single-part arms may be composed of an active intervention (i.e., metformin or lifestyle), standard care, or placebo, and combined-part arms may contain mixed items such as two active interventions (i.e., metformin plus lifestyle), metformin in addition to standard care, or standard care with placebo [23,24,25,35,36,37,38,39,40,41,42,43,44,45,46]. Interventions’ duration varied between 4 and 33.6 months [23,24,25,35,36,37,38,39,40,41,42,43,44,45,46] (Table 3).

#### 3.3.1. Metformin

The total daily intake of metformin also varied between 500 milligrams (mg) and 2000 mg [23,24,25,35,36,37,38,39,40,41,42,43,44,45,46]. One trial included 500 mg for most participants and 1000 mg for part of them during the last few months of the intervention [38], two studies 750 mg [35,39], six 1000 mg [23,36,40,42,43,45], four 1700 mg [34,37,41,44], and two 2000 mg [25,46]. Titration of the drug’s initial dose is reported in four RCTs [24,37,41,46]. The administration of metformin is distributed in equal doses twice daily in thirteen studies [23,24,34,36,37,38,40,41,42,43,44,45,46], three times daily in two studies [35,39], and one study reported the daily distribution of a total amount of 2000 mg in tablets of 500 mg [25] (Table 3).

Eight RCTs reported the assessment of medicine adherence [23,25,37,38,40,41,43,44]. Six of them adopted pill count as the medicine adherence measure [23,37,38,41,43,44]. One study described that tablets were counted every three months, considering a percentage of consumed tablets over 50% as acceptable [38]. This percentage was estimated by diaries where patients recorded consumed tablets and missed doses [38]. Patients received numbered bottles in another trial, and the acceptable percentage after counting tablets was set at more than 80% [41]. Four trials did not provide details concerning pill count [23,37,43,44]. One of them reported interviews in addition to pill count to assess adherence [37], and another reported pill count during every visit [43]. Two trials reported the assessment of metformin adherence at every visit; however, adherence assessment tools were not reported [25,40]. Additionally, one reported that medical doctors (MDs) assessed adherence [40]. Seven studies did not report data regarding medicine adherence [34,35,36,39,42,45,46] (Table 3).

#### 3.3.2. Lifestyle

Lifestyle interventions were applied in seven RCTs and included both dietary and exercise components [24,37,38,39,40,43,44]. The initial lifestyle arm of the United States (US) Diabetes Prevention Program (DPP) [37] was also adopted by two other studies [43,44]. Zhang et al. used the Chinese Diabetes Prevention Program (CDPP) in 2023 [24]. Some lifestyle interventions included motivation strategies [24,37,38,39,40,43,44] (Table 3).

Six trials reported adherence assessment [24,37,38,40,43,44]. Self-reported adherence was assessed in five lifestyle programs [24,37,38,43,44]. Moreover, three of them used questionnaires [24,37,43]. In one study, the adherence was assessed by MDs during visits [40]. Finally, one RCT did not report assessment of lifestyle intervention adherence [39] (Table 3).

#### 3.3.3. Standard Care, Placebo

Standard care was reported in 10 RCTs [23,25,34,37,38,39,40,42,43,45]. In eight RCTs, it consisted mainly of general lifestyle counseling [23,25,34,37,38,39,40,43]. Particularly, two studies reported diet and exercise counseling [23,34]. One trial described healthcare advice [38]. Another RCT applied diabetic education with follow-up education of patients annually [39]. Two studies provided written information, in addition to general diet and exercise counseling [37,44]. They both included an annual individual session for all participants to enhance the adoption of local dietary guidelines for weight loss and increase PA [37,44]. Moreover, one of them included educational materials for T2DM prevention that were provided during sessions [44]. The definition of standard care in another study included one individual session with a physician, a dietitian and a fitness instructor and one group session where patients were given general advice on a healthy diet and increasing PA [43]. Finally, two trials reported standard care for patients with CVD [42,45] (Table 3).

Placebo, when administered, was matched to metformin [34,35,36,37,41,46] (Table 3).

### 3.4. Effectiveness and Safety of Interventions

T2DM was assessed as the primary outcome in eight RCTs which evaluated metformin’s overall effect [23,24,25,35,37,38,40,45], and as the secondary outcome in the other four [34,36,41,42,46]. The primary outcomes in five studies that evaluated the incidence of diabetes as the secondary outcome were changes in body weight and in metabolic and lipidemic profile in patients with overweight/obesity and central adiposity [34], metformin sensitivity and glucose tolerance in patients with prediabetes [36], the progression of mean distal carotid intima–media thickness in patients with coronary heart disease (CHD) [41], cardiovascular risk profile in patients with CHD [42], and left ventricular hypertrophy in patients with CHD and prediabetes [46] (Supplemental Table S2).

T2DM was the primary outcome of four RCTs that evaluated metformin plus lifestyle interventions versus standard care [38,39,40,43]. For two studies including comparators for metformin versus standard care, T2DM was reported as either the primary [38] or secondary outcome [44]. The study that reported the incidence of diabetes as the secondary outcome considered participants’ weight as the primary outcome [44] (Supplemental Table S2).

The diagnosis of T2DM was set by a combined adoption of fasting plasma glucose (FPG), HbA1c, and the two-hour 75 g oral glucose tolerance test (2 h 75 g OGTT) in four RCTs [23,24,25,42], by FPG and 75 g OGTT in four RCTs [37,38,40,43], by FPG and HbA1c in two RCTs [44,46], by 75 g OGTT alone in four studies [34,35,36,39], and by HbA1c alone in two trials [41,45] (Supplemental Table S2).

#### 3.4.1. Effectiveness

##### Overall Effectiveness of Metformin

**Figure 2:**
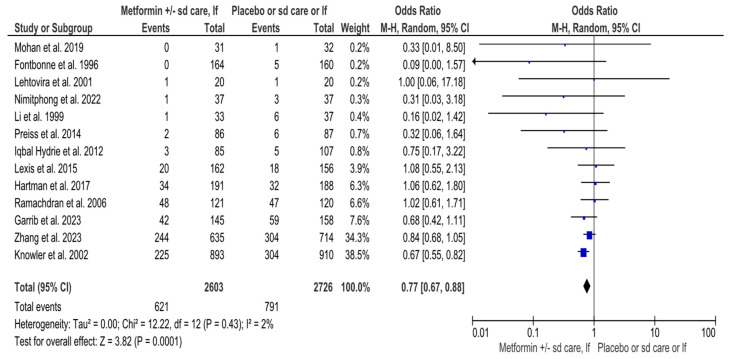
Interventions including metformin and the risk of developing Type-2 Diabetes Mellitus. Sd, standard; lf, lifestyle; Events, number of participants with Type-2 Diabetes Mellitus; Total, number of participants at risk for Type-2 Diabetes Mellitus; blue dots, weight of studies; black blocks, 95% confidence interval of studies; diamond, estimate with 95% confidence interval. Studies are presented in ascending weight [23,24,25,28,34,35,36,37,40,41,42,45,46].

A total of 5329 high-risk individuals (2603 in the intervention and 2726 in the control arm) were analyzed for T2DM in 13 RCTs that evaluated overall effect of metformin in preventing diabetes [31,32,33,34,35,37,38,39,42,43,44,45,46] (Supplemental Table S2) (Figure 2). Among them, 1412 (26.5%) [(621 (23.9%) in the intervention, and 791 (29%) in the control group)] developed T2DM (Supplemental Table S2) (Figure 2). Although the Q statistic was non-significant for heterogeneity (Q 12.22, *p*-value 0.43), the upper limit of the I^2^ index for variability measurements was more than 50% (I^2^ 2%, 95% CI 0, 57%) (Figure 2). Thus, the RE model was used for synthesis (Figure 2). The MA revealed statistically significant results for the overall effect of metformin in preventing T2DM among high-risk adults (OR 0.77, 95% CI 0.67, 0.88, *p*-value 0.0001) (Figure 2).

#### 3.4.2. Subgroup and Sensitivity Analyses, and Meta-Regressions for Overall Effect of Metformin

Due to potential heterogeneity, subgroup and sensitivity analyses were also performed with the RE model. The results remained significant in the subgroup analysis for the study that was conducted in the USA (OR 0.67, 95%CI 0.55, 0.82, *p*-value 0.0001) (Figure S1), and for the following studies: where women comprised the majority of participants (OR 0.76, 95%CI 0.61, 0.95, *p*-value 0.02) (Figure S2); with participants’ mean age greater than 60 years (OR 0.66, 95%CI 0.54, 0.81, *p*-value < 0.0001) (Figure S3); assessing prediabetes as the main risk factor (OR 0.75, 95%CI 0.66, 0.86, *p*-value < 0.0001) (Figure S4); including overweight/obesity as the main risk factor (OR 0.66, 95%CI 0.53, 0.84, *p*-value 0.0006) and for studies not considering overweight/obesity (OR 0.78, 95%CI 0.66, 0.92, *p*-value 0.003) (Figure S5); for studies with a daily metformin dosage of 1700 mg (OR 0.81, 95%CI 0.72, 0.92, *p*-value 0.001) (Figure S8); implementing interventions longer than 18 months (OR 0.78, 95%CI 0.63, 0.96, *p*-value 0.02); (Figure S9); without a post-intervention follow-up (OR 0.75, 95%CI 0.66, 0.86, *p*-value < 0.0001) (Figure S10); and considering the incidence of T2DM as the primary outcome (OR 0.77, 95%CI 0.66, 0.90, *p*-value 0.0007) (Figure S11). On the contrary, a statistically significant result was not found for studies including high-risk patients with either CAD (Figure S6); or HIV infection (Figure S7); [(OR 0.97, 95%CI 0.65, 1.45, *p*-value 0.89) and (OR 0.66, 95%CI 0.41, 1.06, *p*-value 0.09), respectively]. Finally, metformin alone was more effective compared to placebo (OR 0.32, 95%CI 0.11, 0.98, *p*-value 0.05), and when combined with standard care, compared to standard care alone (OR 0.75, 95%CI 0.58, 0.97, *p*-value 0.03) (Figure S12). However, no differences were found for metformin combined with lifestyle interventions against lifestyle interventions alone (OR 0.86, 95%CI 0.71, 1.05, *p*-value 0.15) (Figure S12). Subgroup differences were non-significant in all analyses (Supplemental Table S3).

The results also remained significant in sensitivity analyses exploring the effect of the RCT with the largest sample size (OR 0.84, 95%CI 0.71, 0.99, *p*-value 0.04) (Figure S18), with a post-intervention follow-up (OR 0.75, 95%CI 0.66, 0.86, *p*-value < 0.0001) (Figure S19), and RCTs with a drop-out rate more than 10% (OR 0.78, 95%CI 0.68, 0.89, *p*-value 0.0003) (Figure S20) (Supplemental Table S3). Finally, meta-regression analyses with the intervention’s duration and baseline risk as covariates did not affect the summary OR (Supplemental Table S4).

##### Effectiveness of Metformin Plus Lifestyle Interventions Versus Standard Care

**Figure 3:**
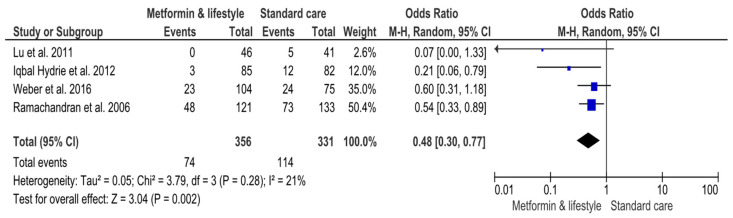
Effects of interventions including metformin and lifestyle interventions on the risk of developing Type-2 Diabetes Mellitus. Events, number of participants with Type-2 Diabetes Mellitus; Total, number of participants at risk for Type-2 Diabetes Mellitus; blue dots, weight of studies; black blocks, 95% confidence interval of studies; diamond, estimate with 95% confidence interval. Studies are presented in ascending weight [38,39,40,43].

Fewer occurrences were identified for patients who received metformin plus lifestyle interventions versus standard care. Particularly, 74 (20.8%) out of 356 participants in mixed interventions developed T2DM versus 114 (34.4%) out of 331 patients in the standard care arm (Supplemental Table S2) (Figure 3). In total, 188 (27.4%) patients were diagnosed with diabetes out of 687 participants (Supplemental Table S2) (Figure 3). The result of the MA was statistically significant (OR 0.48, 95%CI 0.30, 0.77; *p*-value 0.002) (Figure 3). Heterogeneity across studies was non-significant (Q 3.79, *p*-value 0.28) (Figure 3). However, the RE model applied in the MA found the upper limit of I^2^ and CI to be more than 75%, indicating very high variability (I^2^ 21%, 95%CI 0, 90%) (Figure 3).

#### 3.4.3. Subgroup and Sensitivity Analyses, and Meta-Regressions for Metformin Plus Lifestyle Interventions Versus Standard Care

Analyses were also conducted with the RE model. Significant results were found for studies that were performed in India (OR 0.56, 95%CI 0.38, 0.84, *p*-value 0.005) (Figure S13); for studies including participants with a mean age below 60 years (OR 0.52, 95%CI 0.35, 0.76, *p*-value 0.0008) (Figure S14); and for studies with a metformin dosage of 500 mg daily (OR 0.54, 95%CI 0.33, 0.89, *p*-value 0.02) (Figure S15). In subgroup analyses based on study duration and post-intervention follow-up, a significant effect was found for studies that lasted more than six months (Figure S16) and adopted a continuous intervention without post-intervention follow-up (Figure S17) (OR 0.34, 95%CI 0.14, 0.86; *p*-value 0.02). The result was non-significant for the study that implemented interventions up to six months (Figure S16) and assessed post-intervention outcomes (Figure S17) (OR 0.60, 95%CI 0.31, 1.18; *p*-value 0.14). All subgroup analyses had non-statistically significant differences (Supplemental Table S3).

In sensitivity analyses, the studies with the largest sample size (Figure S21), post-intervention duration (Figure S22), and drop-out rate more than 10% (Figure S23) did not have significant results [(OR 0.34, 95%CI 0.12, 0.95, *p*-value 0.04), (OR 0.34, 95%CI 0.14, 0.86, *p*-value 0.02), and OR (0.56, 95%CI 0.38, 0.84, *p*-value 0.005), respectively] (Supplemental Table S3). Finally, meta-regressions for T2DM OR with covariates of intervention duration and baseline risk were non-significant (Supplemental Table S4).

##### Effectiveness of Metformin Versus Standard Care

**Figure 4:**
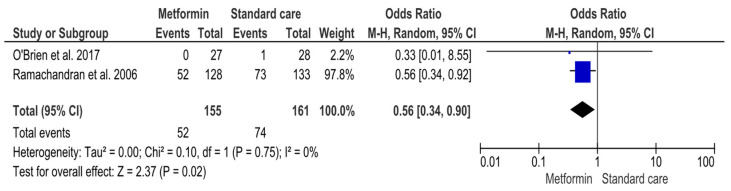
Interventions including metformin compared to standard care and the risk of developing Type-2 Diabetes Mellitus. Events, number of participants with Type-2 Diabetes Mellitus; Total, number of participants at risk for Type-2 Diabetes Mellitus; blue dots, weight of studies; black blocks, 95% confidence interval of studies; diamond, estimate with 95% confidence interval. Studies are presented in ascending weight [38,44].

Two RCTs were included in the analysis comparing metformin versus standard care (Supplemental Table S2) (Figure 4). A total of 155 participants received metformin and 161 received standard care (Supplemental Table S2) (Figure 4). Exploring the comparative effect of metformin versus standard care, fewer diabetes cases were detected in the metformin group compared to the standard care group [52 (33.5%)/74 (46%), respectively]. In total, 126 (39.9%) participants were diagnosed with T2DM (Supplemental Table S2) (Figure 4). The result of the MA was significant (OR 0.56, 95%CI 0.34, 0.90; *p*-value 0.02) (Figure 4). Despite non-significant heterogeneity (Q 0,10, *p*-value 0.75; I^2^ 0), variability across studies cannot be excluded due to the low number of studies and inapplicable 95%CI of I^2^ (Figure 4). Therefore, the data were combined with the RE model (Figure 4).

#### 3.4.4. Safety

Adverse events, when reported, were mainly mild and related to gastrointestinal manifestations and hypoglycemia symptoms [24,25,34,35,36,37,38,39,40,41,44,46]. Other reported events, probably not associated with metformin, included musculoskeletal disorders [37], vertigo [44], headache [34,44], and rash [34,43]. Severe adverse events (i.e., CVD events [38,41,46], neoplasms [41]) or deaths [24,25,38,41] were very rare and were not related to interventions [25] (Supplemental Table S2).

## 4. Quality of Reporting, Potential Bias, and Quality of Evidence

**Figure 5:**
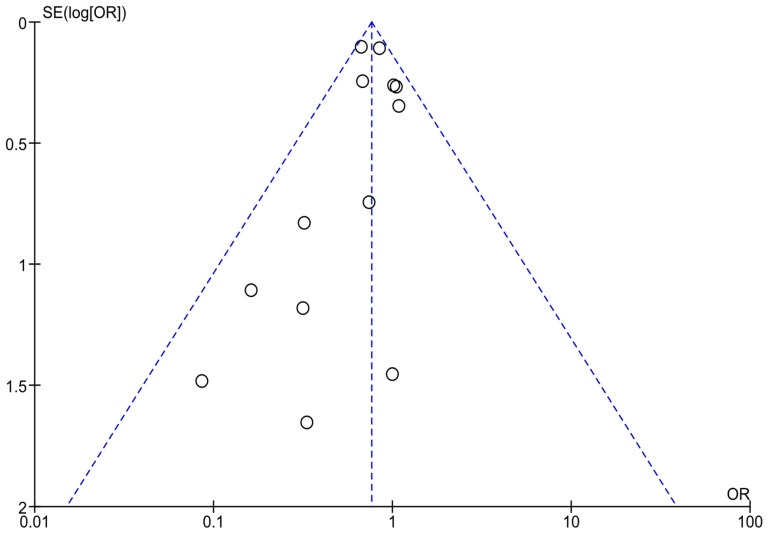
Funnel plots of analyses of studies including metformin.

**Figure 6:**
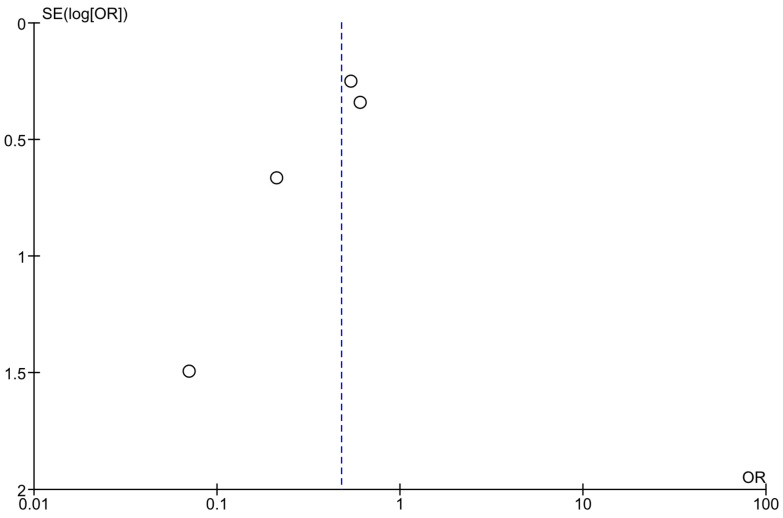
Funnel plots of analyses of studies including metformin and lifestyle interventions.

**Figure 7:**
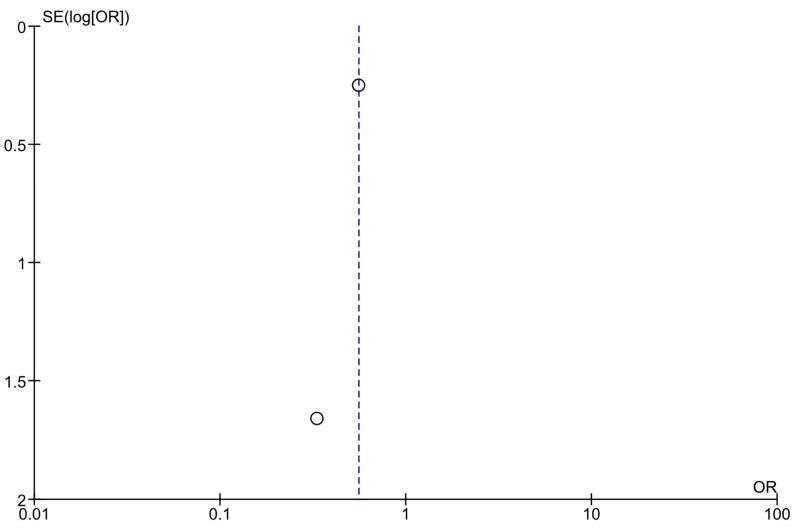
Funnel plots of analyses of studies comparing metformin and standard care.

Optically assessing the funnel plots of our four analyses, the obvious asymmetry found cannot exclude publication bias (Figure 5, Figure 6 and Figure 7). Performing Egger’s test for every separate analysis, the *p*-value was identified as 0.299 in the analysis of metformin’s overall effect, and 0.079 in the analysis of metformin plus lifestyle versus standard care, indicating a non-statistically significant bias.

The funnel plot analyses included studies on the overall effectiveness of metformin in preventing diabetes in high-risk adults [23,24,25,28,34,35,36,37,40,41,42,45,46]; Egger’s test *p*-value = 0.299.

The funnel plot analyses included studies assessing the effectiveness of metformin and lifestyle interventions in preventing diabetes in high-risk adults [38,39,40,43]; Egger’s test *p*-value = 0.079.

The funnel plot analyses included studies comparing metformin versus standard care in preventing diabetes in high-risk adults [38,44].

**Figure 8:**
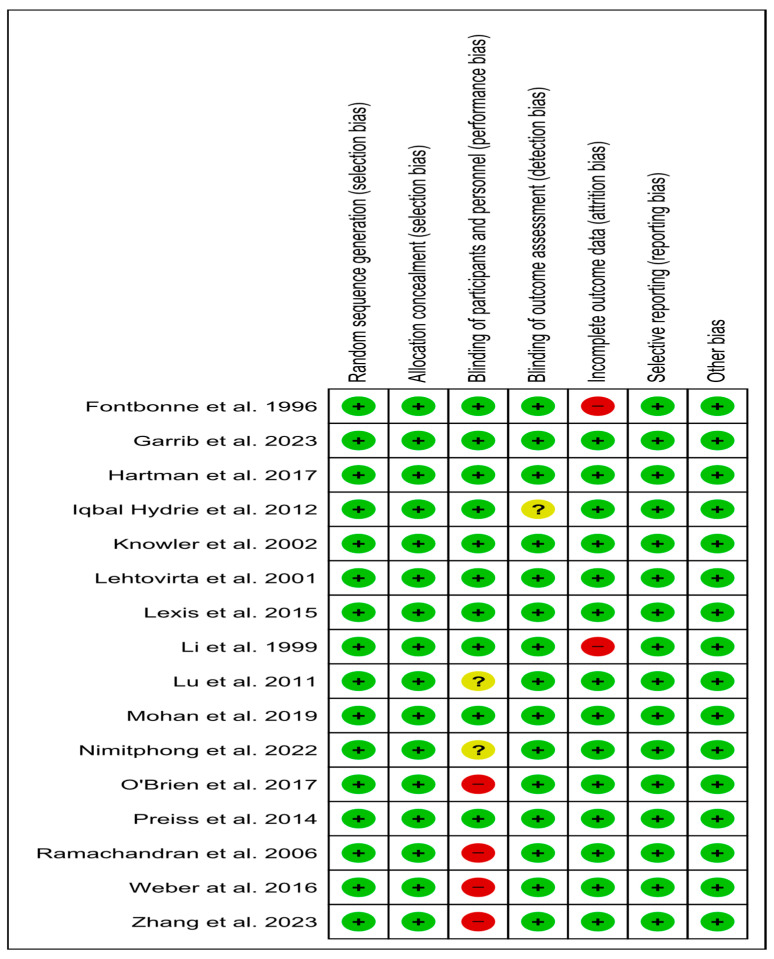
Risk of bias of eligible trials [23,24,25,34,35,36,37,38,39,40,41,42,43,44,45,46].; green color, low risk; red color, high risk; yellow color, unclear risk.

All eligible trials assessed had a low risk of selection, reporting, and other bias [23,24,25,35,36,37,38,39,40,41,42,43,44,45,46]. Evaluating detection bias, 15 trials were judged as low risk [23,24,25,34,35,36,37,38,39,41,42,43,44,45,46], and one did not provide related information, raising an unclear risk [40]. Regarding attrition bias, 14 RCTs were judged as low risk [23,24,25,36,37,38,39,40,41,42,43], and two as high risk [34,35]. Finally, regarding performance bias, the quality characteristics revealed a low risk in ten studies [25,34,35,36,37,40,41,42,45,46], a high risk in four studies [24,38,43,44], and two studies with an unclear risk [23,39] (Figure 8).

The moderate overall quality of evidence demonstrated that persons at risk for T2DM may benefit by reducing diabetes risk with metformin, and metformin combined with lifestyle interventions. The quality of evidence was very low for metformin against standard care (Table 4).

## 5. Discussion

**Figure 9:**
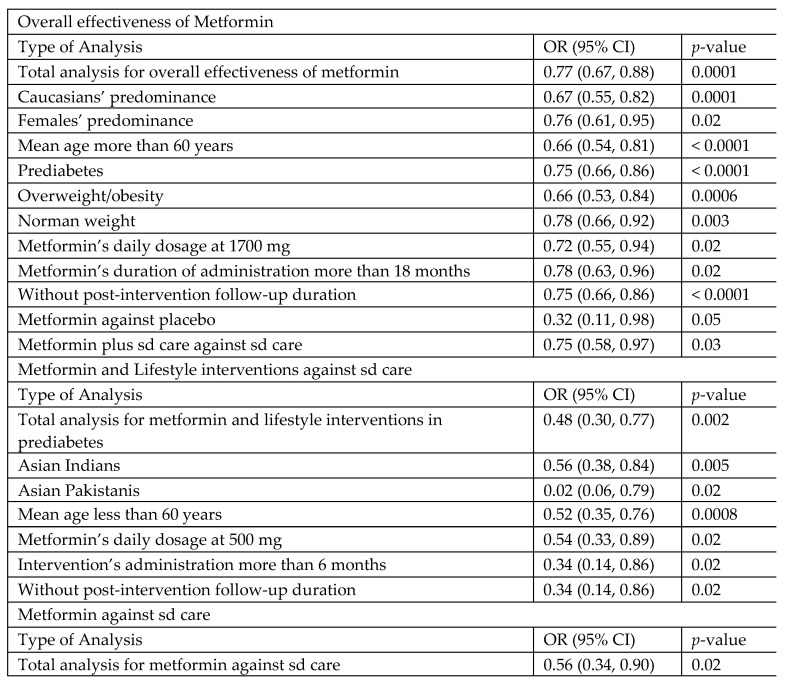
Summary of results for metformin and lifestyle interventions for diabetes prevention in high-risk and metformin-naïve adults. OR, odds ratio; CI, confidence interval; mg, milligram; sd, standard.

In our MA, the results support the beneficial effect of metformin in preventing T2DM among high-risk adults. Interestingly, the beneficial effects concern patients with prediabetes, obese and normal-weight patients, Caucasians, women, and patients over 60 years old. Moreover, it was demonstrated that metformin is effective at a daily dosage of 1700 mg, and after 18 months of administration, while the protective effect weakens after cessation of metformin. Metformin, irrespective of its addition to standard care, is more effective than placebo or standard care alone. Finally, our MA found that metformin and lifestyle interventions are more effective compared to standard care in preventing T2DM in patients with prediabetes. This action is more significant in men and patients younger than 60 years. Interestingly, when combined with lifestyle interventions, metformin’s effective dose is lower than when used alone to prevent T2DM. During the post-intervention period, the benefit weakens. Heterogeneity cannot be excluded in analyses. The quality of evidence is moderate for metformin’s overall effectiveness and for metformin combined with lifestyle interventions, and low for metformin against standard care. Figure 9 summarizes metformin’s overall effectiveness and the effectiveness of metformin combined with lifestyle interventions in preventing T2DM.

### 5.1. Rationale for Eligible Population

In the present MA, we chose to include metformin-naïve patients. Analyzing patients receiving long-term metformin therapy together with metformin-naïve patients might be confusing. We believe that patients receiving metformin or lifestyle interventions on a long-term basis might have different effects concerning the prevention of T2DM compared to patients evaluated from the onset of the intervention. This might hinder the accuracy of comparisons between treatments. To specifically highlight the effect of metformin in preventing T2DM, in our study, we chose to include only drug-naïve patients.

### 5.2. Existing Literature and Comparisons

To our knowledge, this is the first analysis that defines the overall effectiveness of metformin, assessing interactions with placebo, standard care, and lifestyle interventions with special emphasis on studies’ and participants’ characteristics. In the present study, we found that metformin combined with lifestyle interventions is not superior to lifestyle interventions alone, setting lifestyle interventions as a strong factor for diabetes prevention. On the contrary, a previously published MA demonstrated the effectiveness of combined metformin and lifestyle interventions against lifestyle interventions for patients with prediabetes [47]. However, this analysis included pilot RCTs, mixed adolescent and adult populations, and interventions not considered lifestyle adjuncts to metformin (i.e., metformin with placebo, metformin with standard care, or metformin with only exercise not including dietary intervention), reaching a highly biased risk ratio (RR) (RR 0.85) [47]. As far as we know, this is the first study that evaluates metformin and lifestyle interventions versus standard care. We found that the combination of metformin and lifestyle interventions is superior to standard care in patients with prediabetes. Additionally, we found that combined interventions are more effective than metformin alone in preventing T2DM in patients with prediabetes. The effectiveness of metformin alone was confirmed for high-risk patients, as was previously found [13,14,19]. However, in our study, we broadened the inclusion criteria, also considering patients with any diabetes risk factor. In the same line as previous MAs, a protective effect was found for patients with prediabetes [15,16,17,18,19]. Contrariwise to the most recent MA, we excluded RCTs evaluating the effects of other hypoglycemic agents in addition to metformin, and publications reporting secondary analyses of RCTs, which probably conflict with estimations of effectiveness [19]. It should be pointed out that we have included all recently published data.

### 5.3. Interpretation of the Results

#### 5.3.1. Metformin’s Overall Effectiveness

RCTs following patients under metformin and lifestyle interventions for years have declared significant results for both interventions [48,49]. However, the effectiveness of metformin compared to lifestyle interventions is unclear, as the long-term effects of each treatment may have obscured the superiority of one intervention over the other [50,51,52]. In our MA, it was shown that the combination of lifestyle interventions with metformin is not superior to lifestyle interventions alone in preventing T2DM. The combined effect of both interventions was also superior compared to standard care. We have also found that metformin alone is more effective than standard care and placebo. This action might be useful for patients with disabilities, underserved patients, and patients unwilling to adopt lifestyle modifications.

#### 5.3.2. Metformin and Prediabetes

The preventive effect of metformin alone in patients with prediabetes may be attributed to reduced insulin resistance [23,24]. Metformin can also improve endothelial integrity, depicted by a decrease in endothelial dysfunction markers, including soluble intercellular adhesion molecules, soluble vascular cell adhesion molecules, and von Willebrand factors [53]. Long-term use of metformin in patients with prediabetes ameliorates the metabolic profile by reducing systolic and diastolic blood pressure (BP), LDL cholesterol, and triglycerides, and elevating HDL cholesterol levels [16]. Lifestyle interventions alone may also prevent progression to diabetes in patients with prediabetes and reduce participants’ weight [54]. Thus, the combined actions of metformin and lifestyle interventions may be more effective than any intervention alone, explaining our findings.

#### 5.3.3. Metformin and Participants’ Weight

In the present study, we also found that metformin is effective in preventing T2DM in patients with obesity. A recent SR and MA revealed that metformin may significantly reduce body mass index (BMI) in non-diabetic patients with obesity [55]. This action may explain the preventive effect of metformin in obese patients. Additionally, glucagon-like peptide 1 receptor agonists (GLP-1 RAs) may also reduce weight and prevent T2DM in patients with obesity [56,57]. Furthermore, a statistically significant result was also found for patients without obesity. Thus, metformin might be optimal in preventing T2DM in normal-weight patients as well.

#### 5.3.4. Metformin and Cardiovascular Disease

In our study, metformin could not prevent T2DM in patients with CVD. However, it has been estimated that metformin initiated early in patients with prediabetes may reduce total mortality and cardiovascular 10-year risk > 10% in patients without established CVD [58]. Moreover, dapagliflozin is effective in preventing diabetes in patients with CVD [59], and so is semaglutide in patients with obesity and CVD [60]. One RCT with CVD patients that was included in our analysis reported that the treatment was administered for a very short period (four months) [45], while in another three RCTs, the incidence of T2DM was a secondary outcome [41,42,46]. We believe that the treatment duration might have obscured the beneficial effects of metformin in preventing T2DM in CVD patients, while considering the prevention as a primary outcome is also of great importance. In our MA, we found that effectiveness was significant when T2DM was the primary outcome of the studies. Further research is needed for those patients.

#### 5.3.5. Post-Intervention Effectiveness

Investigating the post-intervention effectiveness of both metformin alone and metformin with lifestyle interventions, we found that it might be weakened. Particularly, according to sensitivity analyses on the effect of RCTs that investigated patients who received metformin for 4 months in a 24-month follow-up post-intervention period [45], the overall effectiveness of metformin changed to non-significant. This result may explain potential heterogeneity. Additionally, the secondary analysis of the RCT with the largest sample size [37], analyzing metformin’s post-intervention effects, reported a higher incidence of diabetes [61]. These findings raise a high suspicion that effectiveness decreases after cessation of interventions.

### 5.4. Cost-Effectiveness

Lifestyle interventions have been assessed as cost-effective for quality of life (QUALY), increased survival, disability, and diabetes complications compared to usual care for patients with prediabetes [54]. Additionally, the benefits of lifestyle interventions versus standard care are value for money for diabetes prevention in patients with prediabetes, according to an economic evaluation study [62]. Metformin administration is also cost-effective for QUALY and healthcare systems [63]; however, it is not widely adopted in real life for diabetes prevention, enhancing unfavorable circumstances for both patients and healthcare systems [63]. Moreover, metformin combined with lifestyle interventions against standard care is also cost-effective for patients with prediabetes in India [64]. Incremental cost-effectiveness ratios (ICERs) of USD 145 per percentage point of diabetes risk reduction were found using screening, and USD 14,539 was saved per prevented T2DM case [64]. Finally, either lifestyle interventions or metformin is cost-effective compared to standard care for patients with prediabetes and overweight or obesity in Australia [65]. Particularly, the ICERs for metformin compared to standard care were USD 17,767 and, for lifestyle interventions versus standard care, USD 2702 per quality-adjusted life year gained [65].

### 5.5. Clinical Recommendations and Differences

According to the results of the present study, metformin may have a protective effect at a daily dosage of 1700 mg and after 18 months of administration in high-risk adults with a mean age over 60 years. Concerning specific subgroups of patients that were found to benefit from metformin in the prevention of T2DM, our MA identified Caucasians, women, and patients with prediabetes, obesity, and normal weight.

Metformin, when combined with lifestyle interventions, is effective at a lower dosage of 500 mg daily. The combined action is effective in younger participants with a mean age less than 60 years and when adopted for more than six months. The combination of metformin with lifestyle interventions is effective in Asian Indians, Asian Pakistanis, and men. Metformin and lifestyle interventions may prevent diabetes in patients with prediabetes faster compared to metformin alone. The metformin dosage is lower when combined with lifestyle interventions in younger patients, while the dosage should be increased in older persons. Those differences may be attributed to lower adherence and ability to follow lifestyle programs in older populations. Moreover, lifestyle interventions need ongoing support so that the beneficial effects are maintained in the long term [22].

### 5.6. Strengths and Limitations

To date, our MA is the most comprehensive as it includes updated data. We evaluated patients with any diabetes risk factors and aimed to answer clinical questions that arise in everyday clinical practice. In addition, we included only studies concerning metformin-naïve patients, in order to evaluate and emphasize the true effects of metformin on the prevention of T2DM. In this way, the results are not biased by the time period of metformin treatment. Moreover, the quality of evidence was moderate for the overall effectiveness of metformin and for the combination of metformin with lifestyle interventions. The study also has several limitations. The entire spectrum of patients with any predisposing risk factor (i.e., family history of diabetes, history of GDM, HY, dyslipidemia) is not entirely represented. Additionally, women in metformin and lifestyle interventions are underestimated; therefore, the true effect on women might have been underappreciated. Yet, we have included all the available data of patient populations that have been evaluated in existing RCTs. The combination of metformin with other interventions has not been widely assessed in RCTs across the world. Regarding heterogeneity, it could not be excluded. Subgroup differences may explain heterogeneity; however, sensitivity analyses failed to explain heterogeneity. Additionally, in sensitivity analyses, heterogeneity is also probably present. Finally, the comparison of metformin and standard care had a low sample size with only two RCTs, and the quality of evidence was low.

## 6. Conclusions

In conclusion, metformin alone or combined with lifestyle interventions may prevent T2DM in patients with prediabetes. The combined intervention is more effective in those patients. Additionally, metformin may prevent diabetes in patients with obesity and normal-weight patients as well. Metformin alone is more effective in Caucasian women, patients older than 60 years, and metformin with lifestyle interventions in Asian Indian men and those younger than 60 years. The combined intervention reduces metformin dosage and may act faster than metformin alone. Future research is needed to investigate its effectiveness in patients with more diabetes risk factors worldwide.

## Figures and Tables

**Table 1:** Characteristics of eligible RCTs.

First Author’s Name, Publication Year	Country	No of Arms	No of Centers	Study Duration (mo)	Post-Intervention Follow-Up Duration (mo)	Drop-Out Rate %
Fontbonne, 1996 [34]	France	2	>1, NR	12	0	29
Li, 1999 [35]	China	2	1	12	0	22
Lehtovirta, 2001 [36]	Finland	2	1	18	0	0
Knowler, 2002 [37]	USA	3	27	33.6	0	10
Ramachandran, 2006 [38]	India	4	1	30	0	0
Lu, 2011 [39]	China	2	1	24	0	13
Iqbal Hydrie, 2012 [40]	Pakistan	3	1	18	0	14
Preiss, 2014 [41]	Scotland, UK	2	1	18	0	8
Lexis, 2015 [42]	The Netherlands	2	1	4	0	0
Weber, 2016 [43]	India	2	1	36	30	5
O’Brien, 2017 [44]	USA	3	1	12	0	2
Hartman, 2017 [45]	The Netherlands	2	1	28	24	0
Mohan, 2019 [46]	Scotland, UK	2	1	12	0	7
Nimitphong, 2022 [23]	Thailand	2	1	12	0	0
Zhang, 2023 [24]	China	2	1	48	0	5
Garrib, 2023 [25]	Tanzania	2	>1, NR	12	0	19

RCTs, randomized controlled trials; No, number; mo, months; NR, not reported; USA, United States of America; UK, United Kingdom.

**Table 2:** Characteristics of participants.

First Author’s Name, Publication Year	Sample Size	Male/Female %	Mean Age in Years	Ethnicities (%)	Risk Factors for T2DM	
					Overall	Probable Coexistence
Fontbonne, 1996 [34]	457	33/67	49.5	NR	Overweight/obesity, central adiposity	Family history of T2DM
Li, 1999 [35]	90	71/29	49.5	Asian Chinese (100)	Prediabetes (IGT)	Family history of T2DM
Lehtovirta, 2001 [36]	40	63/37	57.9	Caucasian (100)	First-degree relatives with T2DM, prediabetes (IGT), overweight/obesity	N/A
Knowler, 2002 [37]	3234	34/66	67.5	Caucasian (54.7), African American (19.9), Hispanic (15.7), American Indian (5.3), Asian (4.4) ^1^	Overweight/obesity, prediabetes (IFG and/or IGT)	Family history of T2DM, history of GDM
Ramachandran, 2006 [38]	531	79/21	45.9	Asian Indian (100)	Prediabetes (IGT)	HY, family history of T2DM
Lu, 2011 [39]	110	52/48	63.6	Asian Chinese (100)	Prediabetes (IFG and/or IGT)	N/A
Iqbal Hydrie, 2012 [40]	317	75/25	43.6	Asian Pakistani (100)	Prediabetes (IGT)	N/A
Preiss, 2014 [41]	173	55/45	63.5	Caucasian (100)	CHD, central adiposity	N/A
Lexis, 2015 [42]	346	13/87	58.1	Caucasian (97), Asian (2), African (1)	CAD (STEMI)	N/A
Weber, 2016 [43]	576	63/37	44.4	Asian Indian (100)	Overweight/obesity, central adiposity, prediabetes (IGT)	Family history of T2DM
O’Brien, 2017 [44]	92	0/100	45.1	Hispanic (100)	Prediabetes (IFG, and/or elevated HbA1C)	N/A
Hartman, 2017 [45]	379	75/25	58.1	Caucasian (97), Asian (2), African (1)	CAD (STEMI)	N/A
Mohan, 2019 [46]	68	47/53	64.5	Caucasian (100)	CAD	Prediabetes (IGT), HY
Nimitphong, 2022 [23]	74	69/31	49.5	Asian Thailand (100)	HIV infection, Prediabetes (IFG and/or IGT)	HY, low HDL cholesterol, elevated TG, family history of T2DM, history of GDM
Zhang, 2023 [24]	1678	47/53	53.0	Asian Chinese (100)	Prediabetes (IGT)	N/A
Garrib, 2023 [25]	364	82/18	46.5	African (100)	HIV infection, prediabetes (IFG and/or IGT)	Overweight/obesity

SD, standard deviation; T2DM, Type-2 Diabetes Mellitus; NR, not reported; N/A; not applicable; IGT, impaired glucose tolerance; IFG, impaired fasting glucose; GDM, gestational diabetes mellitus; HY, hypertension; CHD, coronary heart disease; CAD, coronary artery disease; STEMI, ST elevation myocardial infraction; HbA1C, hemoglobin A1C; HIV, human immunodeficiency virus; HDL, high-density lipoprotein; TG, triglyceride. ^1^ In total, 14.1% of them were Pacific Islanders.

**Table 3:** Characteristics of interventions and comparators.

First Author’s Name, Publication Year	Characteristics of Compared Arms			
	Component of Compared Arms	Dosage, Description	Duration (mo)	Assessment of Adherence
Fontbonne, 1996 [34]	Metformin plus Sd Care	Metformin 850 mg twice daily	12	NR
	Sd Care plus Placebo	Lifestyle counseling and matching placebo		
Li, 1999 [35]	Metformin	Metformin 250 mg three times daily	12	NR
	Placebo	Matching placebo		
Lehtovirta, 2001 [36]	Metformin	Metformin 500 mg twice daily	6	NR
	Placebo	Matching placebo		
Knowler, 2002 [37]	Metformin plus Sd Care	Metformin 850 mg twice daily; initial dose 850 mg once daily for one mo.	33.6 ^1^	Pill counts and structured interview
		Written information, annual individual lifestyle counseling.		
	Lifestyle	Ιntensive face-to-face and group program. Low-calorie and low-fat diet. Moderate aerobic PA for 150 min per wk. Motivational strategies.		Self-reported, questionaires
	Sd Care plus Placebo	Written information, annual individual lifestyle counseling.		Pill counts and structured interview
		Matching placebo		
Ramachandran, 2006 [38]	Metformin plus Lifestyle	Metformin 250 mg twice daily; 500 mg twice daily for the first 50 subjects for 40 days during the last 12 mo.	30	Diaries, pill count, self-reported
		Low-calorie diet with refined carbohydrates and fats, fiber-rich foods and avoidance of sugar. Moderate aerobic PA for 30 min daily at least. Motivation strategies.		Self-reported
	Metformin	Metformin 250 mg twice daily; 500 mg twice daily for the first 50 subjects for 40 days during the last 12 mo.		Pill count
	Lifestyle	Low-calorie diet with refined carbohydrates and fats, fiber-rich foods and avoidance of sugar. Moderate aerobic PA for 30 min daily at least. Motivation strategies.		Self-reported
	Sd Care	Lifestyle counseling		N/A
Lu, 2011 [39]	Metformin plus Lifestyle	Metformin 250 mg three times daily	24	NR
		Lectures on diet and exercise given face-to-face once every 3 mo and by telephone once per mo. Motivation strategies.		
	Sd Care	Health education and follow-up		
Iqbal Hydrie, 2012 [40]	Metformin plus Lifestyle	Metformin 500 mg twice daily	18	MD
		Intensive program. Dietary modification with total fat intake < 30% of total daily energy consumed and fiber intake of 15 g/1000 kcal. Moderate aerobic PA for 30 min daily at least. Motivation strategies.		
	Lifestyle	Dietary modification with total fat intake < 30% of total daily energy consumed and fiber intake of 15 g/1000 kcal. Moderate aerobic PA for 30 min daily at least. Motivation strategies.		MD
	Sd Care	Lifestyle counseling		N/A
Preiss, 2014 [41]	Metformin	850 mg twice daily; initial dose 850 mg once daily for one wk.	18	Tablet counts of numbered bottles
	Placebo	Matching placebo		
Lexis, 2015 [42]	Metformin plus Sd Care	Metformin 500 mg twice daily	4	NR
		Sd care		
	Sd Care	Sd care		N/A
Weber, 2016 [43]	Metformin plus Lifestyle	Metformin 500 mg twice daily	6	Pill count
		Adaption of the US DPP lifestyle program, including motivational strategies [29].		Self-reported, questionaires
	Sd Care	Lifestyle counseling		N/A
O’Brien, 2017 [44]	Metformin	Metformin 850 mg twice daily	12	Pill counts and structured interview
	Lifestyle	Adaption of the US DPP lifestyle program, including motivational strategies [29]		Self-reported
	Sd Care	Written information, annual individual lifestyle counseling.		N/A
Hartman, 2017 [45]	Metformin plus Sd Care	Metformin 500 mg twice daily	4	NR
		Sd care		
	Sd Care	Sd care		N/A
Mohan, 2019 [46]	Metformin	Metformin 1000 mg twice daily; initial dosage 500 mg twice daily for two wks.	12	NR
	Placebo	Matching placebo		
Nimitphong, 2022 [23]	Metformin plus Sd Care	Metformin 500 mg twice daily	12	Pill count
		Lifestyle counseling		
	Sd Care	Lifestyle counseling		N/A
Zhang, 2023 [24]	Metformin plus Lifestyle	Metformin 850 mg twice daily; initial dosage 850 mg daily for two wks.	24	Pill count
		Dietary modification with vegetable intake ≥500 g daily, reduced carboxylate intake by 50 g per meal for participants’ BMI ≥ 25 kg/m^2^, no consumption of sugar-sweetened beverages, no food intake after dinner and eating out up to one time per wk. Moderate aerobic PA for 30 min daily at least, five days per wk.		Self-reported, questionnaires
	Lifestyle	Dietary modification with vegetable intake ≥500 g daily, reduced carboxylate intake by 50 g per meal for participants’ BMI ≥ 25 kg/m^2^, no consumption of sugar-sweetened beverages, no food intake after dinner, and eating out up to one time per wk. Moderate aerobic PA for 30 min daily at least, five days per wk.		Self-reported questionnaires
Garrib, 2023 [25]	Metformin plus Sd Care	Metformin 2000 mg daily, dispensed in 500 mg tablets.	12	Medicine adherence at every visit
		Lifestyle counseling		
	Sd Care	Lifestyle counseling		N/A

mo, months; Sd, standard; mg, milligram; NR, non-reported; PA, physical activity; min, minute; wk, week; N/A, not applicable; MD, medical doctor; g, gram; kcal, kilocalories; US, United States; DPP, Diabetes Prevention Program; BMI; body mass index; kg, kilogram; m, meter. ^1^ 33.6 months on average; range: 21.6–55.2 months.

**Table 4:** GRADE evaluation of the overall evidence of studies according to analyses.

Evidence of metformin-including interventions for Type-2 Diabetes Mellitus prevention						
Patient or population: patients at risk for Type-2 Diabetes Mellitus Settings: randomized controlled trials Intervention–Control: (1) metformin’s overall effect, (2) metformin plus lifestyle–standard care, (3) metformin–standard care						
Outcomes	Illustrative comparative risks * (95% CI)		Relative effect (95% CI)	No. of participants (studies)	Quality of the evidence (GRADE)	Comments
	Assumed risk	Corresponding risk				
	Placebo	Metformin				
(1) Type-2 Diabetes	Study population		OR 0.76 (0.67 to 0.86)	5329 (13 studies)	moderate	
	290 per 1000	237 per 1000 (215 to 260)				
(2) Type-2 Diabetes	Study population		OR 0.48 (0.30 to 0.77)	687 (4 studies)	moderate	
	344 per 1000	201 per 1000 (136 to 288)				
(3) Type-2 Diabetes	Study population		OR 0.56 (0.34 to 0.90)	316 (2 studies)	low	
	460 per 1000	323 per 1000 (224 to 434)				

* The basis for the assumed risk (e.g., the median control group risk across studies) is provided in footnotes. The corresponding risk (and its 95% confidence interval) is based on the assumed risk in the comparison group and the relative effect of the intervention (and its 95% CI). CI: confidence interval; OR: odds ratio; GRADE Working Group grades of evidence; high quality: further research is very unlikely to change our confidence in the estimate of effects; moderate quality: further research is likely to have an important impact on our confidence in the estimate of effects and may change the estimate; low quality: further research is very likely to have an important impact on our confidence in the estimate of effects and is likely to change the estimate; very low quality: we are very uncertain about the estimate.

## Data Availability

Data are contained within the article.

## Supplementary Materials

The following supporting information can be downloaded at https://www.mdpi.com/article/10.3390/jcm14144947/s1: Figure S1:Subgroup Analysis for overall effectiveness of metformin based on same performance countries; Figure S2: Subgroup Analysis for overall effectiveness of metformin based on gender predominance; Figure S3: Subgroup Analysis for overall effectiveness of metformin based on participants’ mean age; Figure S4: Subgroup Analysis for overall effectiveness of metformin based on prediabetes as diabetes’ risk factor; Figure S5: Subgroup Analysis for overall effectiveness of metformin based on overweight/obesity as diabetes’ risk factor; Figure S6: Subgroup Analysis for overall effectiveness of metformin based on cardiovascular disease as diabetes’ risk factor; Figure S7: Subgroup Analysis for overall effectiveness of metformin based on Human Immunodeficiency Virus as diabetes’ risk factor; Figure S8: Subgroup Analysis for overall effectiveness of metformin based on metformin’s daily dosage; Figure S9: Subgroup Analysis for overall effectiveness of metformin based on metformin’s intervention duration.; Figure S10: Subgroup Analysis for overall effectiveness of metformin based on metformin’s post-intervention duration; Figure S11: Subgroup Analysis for overall effectiveness of metformin based on diabetes’ outcome assessment; Figure S12: Subgroup Analysis for overall effectiveness of metformin based on studies’ compared arms characteristics.; Figure S13: Subgroup Analysis for effectiveness of metformin and lifestyle interventions based on same performance countries; Figure S14: Subgroup Analysis for effectiveness of metformin and lifestyle interventions based on participants’ mean age; Figure S15: Subgroup Analysis for effectiveness of metformin and lifestyle interventions based on metformin’s daily dosage; Figure S16: Subgroup Analysis for effectiveness of metformin and lifestyle interventions based on metformin’s intervention duration; Figure S17: Subgroup Analysis for effectiveness of metformin and lifestyle interventions based on metformin’s post-intervention duration; Figure S18: Sensitivity Analysis for RCT with the largest sample size in overall effectiveness of metformin; Figure S19: Sensitivity Analysis for RCT with post-intervention in overall effectiveness of metformin; Figure S20: Sensitivity Analysis for RCTs with drop-out rate more than 10% in overall effectiveness of metformin; Figure S21: Sensitivity Analysis for RCT with the largest sample size in effectiveness of metformin and lifestyle interventions; Figure S22: Sensitivity Analysis for RCT with post-intervention in effectiveness of metformin and lifestyle interventions; Figure S23: Sensitivity Analysis for RCTs with drop-out rate more than 10% in effectiveness of metformin and lifestyle interventions; Table S1: Search strategy; Table S2: Efficacy and safety of Metformin’s included interventions in preventing Type-2 Diabetes; Table S3: Subgroup and sensitivity analyses for metformin’s overall effectiveness and metformin with lifestyle interventions compared to standard care; Table S4. Meta-regression for T2DM OR in metformin’s overall effect and metformin combined with lifestyle interventions.
