# Supplementary material for: Metformin’s Overall Effectiveness and Combined Action with Lifestyle Interventions in Preventing Type-2 Diabetes Mellitus in High-Risk Metformin-Naïve Patients: An Updated Systematic Review and Meta-Analysis of Published RCTs
Source: J Clin Med. 2025 Jul 12;14(14):4947. doi: 10.3390/jcm14144947 (PMC12295976; doi:10.3390/jcm14144947)
Supplement: Supplementary file 1 [file jcm-14-04947-s001.zip › jcm-3735639-supplementary.pdf]

Metformin's overall effectiveness and combined action with lifestyle interventions in preventing Type-2 Diabetes Mellitus in high-risk metformin naïve patients: An updated Systematic Review and Meta-Analyses of published RCTs

Georgios I. Tsironikos[1], Vasiliki Tsolaki*[2], George Zakynthinos[3], Vasiliki Rammou[4], Despoina Kyprianidou[5], Thomas Antonogiannis[6], Epameinondas Zakynthinos[2], Alexandra Bargiota[7]

1. Department of Research for General Medicine and Primary Health Care, Faculty of Medicine, University of Ioannina, Ioannina, University Campus, 45110 Ioannina, Greece; g.tsironikos@uoi.gr

2. Department of Critical Care, University Hospital of Larissa, Faculty of Medicine, University of Thessaly, Mezourlo, 41335 Larissa, Greece; vastsolaki@uth.gr; ezakynth@med.uth.gr

3. 3rd Department of Cardiology, "Sotiria" Chest Diseases Hospital, Medical School, National and Kapodistrian University of Athens, 11527 Athens, Greece; gzakynthinos@uth.gr

4. Faculty of Medicine, University of Thessaly, Mezourlo, 41335 Larissa, Greece; vas19ram@gmail.com; thomas.antonogiannis@gmail.com

5. Medical School, National and Kapodistrian University of Athens, 11527 Athens, Greece; depykyp@med.uoa.gr

6. Department of Internal Medicine-Endocrinology, University Hospital of Larissa, Faculty of Medicine, University of Thessaly, Mezourlo, 41335 Larissa, Greece; abargio@med.uth.gr

*Correspondence: vastsolaki@uth.gr

Supplemental Table S1. Search strategy

PubMed	(diet OR nutrition) OR (exercise OR "physical activit*" OR workout) OR (lifestyle) OR (metformin) AND ((diabetes)) AND ("Clinical Trials as Topic"[Mesh] OR "randomized controlled trial"[pt] OR "controlled clinical trial"[pt] OR randomized[tiab] OR placebo[tiab] OR randomly[tiab] OR trial[tiab])<br><br>with PubMed publication date Between Jan 1950 and May 2025
CENTRAL	((diet OR nutrition) OR (exercise OR “physical NEXT activit” OR workout) OR (lifestyle) OR (metformin)) AND (diabetes)<br><br>with Cochrane Library publication date from Jan 1950 to May 2025, (Word variations have been searched)

Supplemental Table S2. Efficacy and safety of Metformin's included interventions in preventing Type-2 Diabetes

Overall effectiveness and safety of metformin					
**First author's name, publication year**	**Outcome of T2DM assessed as primary or secondary**	**Participants at risk for T2DM (I / C)**	**Events of T2DM n (%) (I / C)**	**Diagnostic modalities**	**Total adverse events, side effects n (%) (I / C)**
Fontbonne, 1996 [34]	Secondary	164 / 160	0 (0) / 5 (3.1)	2h 75 g OGTT	Diarrhea 45 (48.9) / 10 (23.8), Nausea/vomiting 14 (15.2) / 6 (14.3), Abdominal pain 7 (7.6) / 11 (26.2), Constipation 2 (2.2) / 0 (0), Cramps 1 (1.1) / 1 (2.4), Headache/fatigue 3 (3.3) / 2 (4.8), Mood shifts 2 (2.2) / 1 (2.4), Cutaneous rash 1 (1.1) / 4 (9.6), Hunger 3 (3.3) / 2 (4.8), Bud taste in mouth 0 (0) / 4 (9.6)
Li, 1999 [35]	Primary	33 / 37	1 (3) / 6 (16.2)	2h 75 g OGTT	NR
Lehtovirta, 2001 [36]	Secondary	20 / 20	1 (5) / 1 (5)	2h 75 g OGTT	Mild gastrointestinal symptoms 8 (40) / 5 (25)
Knowler, 2002 [37]	Primary	893 / 910	225 (25.2) / 304 (33.4)	FPG, 2h 75 g OGTT	Gastrointestinal symptoms 543 (77.8) / 196 (30.7), Musculoskeletal symptoms 140 (20) / 135 (21.1) [1, 2]
Ramachandran, 2006 [38]	Primary	51 / 52	19 (37.2) / 20 (38.5)	FPG, 2h 75 g OGTT	Gastrointestinal symptoms 22 (8.4) / 0 (0) [3], Hypoglycemia symptoms 5 (1.9) / 0 (0) [4], CVD events 5 (3.9) / 4 (3), Deaths 1 (0.8) / 1 (0.7)
Iqbal Hydrie, 2012 [40]	Primary	85 / 107	3 (3.5) / 5 (4.7)	FPG, 2h 75 g OGTT	Gastrointestinal or hypoglycemia symptoms 5 (5.3) / 0 (0.0)
Preiss, 2014 [41]	Secondary	86 / 87	2 (2) / 6 (7)	HbA1c	Gastrointestinal symptoms 28 (32.6) / 5 (5.7), CVD events 7 (8.1) / 16 (18.4), Newly diagnosed neoplasm 1 (1.0) / 4 (5), Deaths 1 (1) / 0 (0)
Lexis, 2015 [42]	Secondary	162 / 156	20 (12.3) / 18 (11.5)	FPG, 2h 75 g OGTT, HbA1c	NR
Hartman, 2017 [45]	Primary	191 / 188	34 (17.8) / 32 (17.0)	HbA1c	NR
Mohan, 2019 [46]	Secondary	31 / 32	0 (0) / 1 (3.1)	FPG, HbA1c	Mild-to-serious gastrointestinal symptoms 24 (70.6) / 19 (55.9), Stroke 1 (2.9) / 0 (0)
Nimitphong, 2022 [23]	Primary	37 / 37	1 (2.7) / 3 (8.1)	FPG, 2h 75 g OGTT, HbA1c	NR
Zhang, 2023 [24]	Primary	635 / 714	244 (38.4) / 304 (24.6)	FPG, 2h 75 g OGTT, HbA1c	Gastrointestinal symptoms 191 (22.9) / 8 (0.9), Hypoglycemia symptoms 13 (1.6) / 6 (0.7), Hyperlipemia 37 (4.4) / 57 (6.7), Deaths 1 (0.1) / 0 (0)
Garrib, 2023 [25]	Primary	145 / 158	42 (28.9) / 59 (37.3)	FPG, 2h 75 g OGTT, HbA1C [5]	Mild-to-moderate gastrointestinal symptoms 118 (64.9) / 96 (52.7), serious adverse events or deaths 9 (4.9) / 9 (4.9) [6]
**Effectiveness and safety of metformin plus lifestyle interventions vs sd care**					
**First author's name, publication year**	**Outcome of T2DM assessed as primary or secondary**	**Patients at risk for T2DM that were analyzed (I / C)**	**Events of T2DM n (%) (I / C)**	**Diagnostic modalities**	**Adverse events / side effects (I / C)**
Ramachandran, 2006 [38]	Primary	121 / 133	48 (39.7) / 73 (54.9)	FPG, 2h 75 g OGTT	Gastrointestinal symptoms 22 (8.4) / 0 (0) [3], Hypoglycemia symptoms 5 (1.9) / 0 (0) [4], CVD events 5 (3.9) / 2 (1.5), Deaths 1 (1.9) / 0 (0)
Lu, 2011 [39]	Primary	46 / 41	0 (0) / 5 (12.2)	2h 75 g OGTT	NR
Iqbal Hydrie, 2012 [40]	Primary	85 / 82	3 (3.5) / 12 (14.6)	FPG, 2h 75 g OGTT	Gastrointestinal or hypoglycemia symptoms 5 (5.3) / 0 (0)
Weber, 2016 [43]	Primary	104 / 75 [7]	23 (22.1) / 24 (32) [8]	FPG, 2h 75 g OGTT	Rash 1 (0.3) / 0 (0)
**Effectiveness and safety of metformin vs sd care**					
**First author's name, publication year**	**Outcome of T2DM assessed as primary or secondary**	**Participants at risk for T2DM (I / C)**	**Events of T2DM n (%) (I / C)**	**Diagnostic modalities**	**Adverse events / side effects (I / C)**

Ramachandran, 2006 [38]	Primary	128 / 133	52 (41) / 73 (54.9)	FPG, 2h 75 g OGTT	Gastrointestinal symptoms 22 (8.4) / 0 (0) [3], Hypoglycemia symptoms 5 (1.9) / 0 (0) [4], CVD events 0 (0) / 2 (1.5)
O'Brien, 2017 [44]	Secondary	27 / 28	0 (0) / 1 (3.6)	FPG, HbA1c	Gastrointestinal symptoms 8 (27.6) / 0 (0.0, dizziness/vertigo 1 (3.4) / 0 (0.0), headache 1 (3.4) / 0 (0.0)

T2DM, Type-2 Diabetes Mellitus; I, Intervention; C, Comparator; h, hour; g, gram; OGTT, oral glucose tolerance test; NR, non-reported; FPG, fasting plasma glucose; CVD, cardiovascular disease; HbA1c, Hemoglobin A1c; vs, versus. [1] adverse events at year 4; [2] hospitalizations or deaths not-associated with intervention; [3] total number and percentage in both lifestyle plus metformin and metformin group participants (n = 262); [4] total number and percentage in both lifestyle plus metformin and metformin group participants (n = 262); [5] different results by any modality; diagnoses based on HbA1c (more cases than FPG, 2 h 75g OGTT); [6] serious adverse events or deaths not-associated with intervention; [7] participants at risk for diabetes at year 3; [8] events of diabetes at year 3

Supplemental Table S3. Subgroup and sensitivity analyses for metformin's overall effectiveness and metformin with lifestyle interventions compared to standard care

**Subgroup Analyses**								
**Overall effectiveness of metformin**								
**Factor**	**Subgroup**	**No of studies**	**OR (95% CI)**	**P-value**	**Cochran's Q statistic**	**Cochran's Q statistic P-value**	**I^2 (95% CI)**	**P-value test of difference**
Similar Countries	Asia (China, Thailand)	3	0.58 (0.23, 1.46)	0.25	2.87	0.24	30% (0, 94%)	0.34
	Asia (India)	1	1.02 (0.61, 1.71)	0.94	N/A	N/A	N/A	
	Asia (Pakistan)	1	0.75 (0.17, 3.22)	0.69	N/A	N/A	N/A	
	Europe (Scotland, UK)	2	0.32 (0.08, 1.39)	0.13	0.00	0.98	0% (N/A)	
	Europe (Netherlands)	2	1.06 (0.70, 1.62)	0.77	0.00	0.96	0% (N/A)	
	Europe (France)	1	0.09 (0.00, 1.57)	1.00	N/A	N/A	N/A	
	Europe (Finland)	1	1.00 (0.06, 17.18)	1.00	N/A	N/A	N/A	
	North America (USA)	1	0.67 (0.55, 0.82)	0.0001	N/A	N/A	N/A	
	Africa (Tanzania)	1	0.68 (0.42, 1.11)	0.12	N/A	N/A	N/A	
Gender	Male more than female	8	0.82 (0.62, 1.09)	0.17	6.23	0.51	0% (0, 62%)	0.68
	Female more than male	5	0.76 (0.61, 0.95)	0.02	5.69	0.22	30% (0, 86%)	
Mean age	More than 60 years	3	0.66 (0.54, 0.81)	< 0.0001	0.95	0.62	0% (0, 49%)	0.06
	Less than 60 years	10	0.85 (0.72, 1.00)	0.06	7.81	0.55	0% (0, 57%)	
Prediabetes as main risk factor	Included	8	0.75 (0.66, 0.86)	< 0.0001	6.21	0.52	0% (0, 62%)	0.66
	Not included	5	0.85 (0.50, 1.45)	0.55	5.12	0.28	22% (0, 84%)	
Overweight/obesity as main risk factor	Included	3	0.66 (0.53, 0.84)	0.0006	2.01	0.37	0% (0, 88%)	0.27
	Not included	10	0.78 (0.66, 0.92)	0.003	7.76	0.56	0% (0, 56%)	
CVD as main risk factor	Included	4	0.97 (0.65, 1.45)	0.89	2.38	0.50	0% (0, 88%)	0.23
	Not included	9	0.75 (0.64, 0.87)	0.0001	8.36	0.40	4% (0, 65%)	
HIV infection as main risk factor	Included	2	0.66 (0.41, 1.06)	0.09	0.42	0.52	0% (N/A)	0.49
	Not included	11	0.79 (0.67, 0.94)	0.007	11.39	0.33	12% (0, 65%)	
Metformin's daily dosage	500 mg	1	1.02 (0.61, 1.71)	0.94	N/A	N/A	N/A	0.26
	750 mg	1	0.16 (0.02, 1.42)	0.10	N/A	N/A	N/A	
	1000 mg	5	1.00 (0.68, 1.48)	1.00	1.21	0.88	0% (0, 33%)	
	1700 mg	4	0.72 (0.55, 0.94)	0.02	5.33	0.15	44% (0, 95%)	
	2000 mg	2	0.67 (0.42, 1.09)	0.10	0.19	0.67	0% (N/A)	
Intervention's duration	More than 18 mo	3	0.78 (0.63, 0.96)	0.02	3.50	0.17	43% (0, 93%)	0.99
	Up to 18 mo	10	0.78 (0.58, 1.05)	0.10	8.77	0.46	0% (0, 61%)	
Post-intervention follow-up	Included	1	1.06 (0.62, 1.80)	0.84	N/A	N/A	N/A	0.23
	Not included	12	0.75 (0.66, 0.86)	< 0.0001	10.75	0.46	0% (%)	
T2DM outcome assessment	Primary	8	0.77 (0.66, 0.90)	0.0007	7.66	0.36	9% (0, 69%)	0.72
	Secondary	5	0.67 (0.30, 1.47)	0.31	4.68	0.32	15% (0, 87%)	
Types of intervention/control	Metformin vs Placebo	4	0.32 (0.11, 0.98)	0.05	1.00	0.80	0% (0, 73%)	0.18
	Metformin plus sd care vs sd care	6	0.75 (0.58, 0.97)	0.03	6.42	0.27	22% (0, 81%)	
	Metformin plus lifestyle intervention vs lifestyle intervention	3	0.86 (0.71, 1.05)	0.15	0.50	0.78	0% (0, 50%)	
**Metformin and lifestyle interventions vs sd care**								
**Factor**	**Subgroup**	**No of studies**	**OR (95% CI)**	**P-value**	**Cochran's Q statistic**	**Cochran's Q statistic P-value**	**I^2 (95% CI)**	**P-value test of difference**
Similar Countries	Asia (India)	2	0.56 (0.38, 0.84)	0.005	0.07	0.8	0% (N/A)	0.16
	Asia (China)	1	0.07 (0.00, 1.33)	0.08	N/A	N/A	N/A	
	Asia (Pakistan)	1	0.02 (0.06, 0.79)	0.02	N/A	N/A	N/A	
Mean age	More than 60 years	1	0.07 (0.00, 1.33)	0.08	N/A	N/A	N/A	0.19
	Less than 60 years	3	0.52 (0.35, 0.76)	0.0008	2.01	0.37	1% (0, 88%)	
Metformin's daily dosage	500 mg	1	0.54 (0.33, 0.89)	0.02	N/A	N/A	N/A	0.38
	750 mg	1	0.07 (0.00, 1.33)	0.08	N/A	N/A	N/A	
	1700 mg	2	0.42 (0.16, 1.11)	0.08	1.95	0.16	49% (N/A)	
Intervention's duration	More than 6 mo	3	0.34 (0.14, 0.86)	0.02	3.36	0.19	40% (0, 93%)	0.43
	Up to 6 mo	1	0.60 (0.31, 1.18)	0.14	N/A	N/A	N/A	
Post-intervention follow-up	Included	1	0.60 (0.31, 1.18)	0.14	N/A	N/A	N/A	0.33
	Not included	3	0.34 (0.14, 0.86)	0.02	3.36	0.19	40% (0, 93%)	

Sensitivity Analyses							
**Overall effectiveness of metformin**							
**Factor**	**Subgroup**	**No of studies**	**OR (95% CI)**	**P-value**	**Cochran's Q statistic**	**Cochran's Q statistic P-value**	**I^2 (95% CI)**
Effect of RCT with the largest sample size	Lower sample size	12	0.84 (0.71, 0.99)	0.04	9.48	0.58	0% (0, 50%)
Effect of RCT with post-intervention follow-up	Without post-intervention follow-up	12	0.75 (0.66, 0.86)	< 0.0001	10.75	0.46	0% (0, 57%)
Effect of RCTs with drop-out rate more than 10%	With drop-out rate up to 10%	9	0.78 (0.68, 0.89)	0.0003	7.73	0.46	0% (0, 65%)
**Metformin and lifestyle interventions vs sd care**							
**Factor**	**Subgroup**	**No of studies**	**OR (95% CI)**	**P-value**	**Cochran's Q statistic**	**Cochran's Q statistic P-value**	**I^2 (95% CI)**
Effect of RCT with the largest sample size	Lower sample size	3	0.34 (0.12, 0.95)	0.04	3.62	0.16	45% (0, 93%)
Effect of RCT with post-intervention follow-up	Without post-intervention follow-up	3	0.34 (0.14, 0.86)	0.02	3.36	0.19	40% (0, 93%)
Effect of RCTs with drop-out rate more than 10%	With drop-out rate up to 10%	2	0.56 (0.38, 0.84)	0.005	0.07	0.80	0% (N/A)

No, number; OR, odds ratio; CI, confidence interval; N/A, not applicable; UK, United Kingdom; USA, United States of America; CVD, cardiovascular disease; HIV, Human Immunodeficiency Virus; mg, milligram; mo, months; T2DM, Type-2 Diabetes Mellitus; vs, versus; sd, standard; RCT, randomized controlled trial

Supplemental Table S4. Meta-regression for T2DM OR in metformin's overall effect and metformin combined with lifestyle interventions.

**Overall effectiveness of metformin**					
**Covariate**	**No of Studies**	**Coefficient b (95% CI)**	**SE**	**P-value**	**t**
Intervention duration (mo)	13	0.015 (-0.02224, 0.05224)	0.019	0.437	0.807
Baseline risk	13	2.309 (-0.52908, 5.14708)	1.448	0.139	1.595
**Metformin and lifestyle interventions**					
**Covariate**	**No of Studies**	**Coefficient b (95% CI)**	**SE**	**P-value**	**t**
Intervention duration (mo)	4	-0.029 (-0.1564, 0.0984)	0.065	0.698	-0.449
Baseline risk	4	3.940 (-0.36612, 8.24612)	2.197	0.215	1.793

T2DM, Type-2 Diabetes Mellitus, No, numbers; CI, confidence interval; SE, standard error; mo, months

Supplemental Figure S1. Subgroup Analysis for overall effectiveness of metformin based on same performance countries.

Study or Subgroup	Metformin +/- sd care, lf Events	Metformin +/- sd care, lf Total	Placebo or sd care or lf Events	Placebo or sd care or lf Total	Weight	Odds Ratio M-H, Random, 95% CI
**1.2.1 Asia (China, Thailand)**						
Nimitphong et al. 2022	1	37	3	37	0.3%	0.31 [0.03, 3.18]
Li et al. 1999	1	33	6	37	0.4%	0.16 [0.02, 1.42]
Zhang et al. 2023	244	635	304	714	34.3%	0.84 [0.68, 1.05]
**Subtotal (95% CI)**		**705**		**788**	**35.0%**	**0.58 [0.23, 1.46]**
Total events	246		313			
Heterogeneity: Tau² = 0.29; Chi² = 2.87, df = 2 (P = 0.24); I² = 30%						
Test for overall effect: Z = 1.15 (P = 0.25)						
**1.2.2 Asia (India)**						
Ramachdran et al. 2006	48	121	47	120	6.6%	1.02 [0.61, 1.71]
**Subtotal (95% CI)**		**121**		**120**	**6.6%**	**1.02 [0.61, 1.71]**
Total events	48		47			
Heterogeneity: Not applicable						
Test for overall effect: Z = 0.08 (P = 0.94)						
**1.2.3 Asia (Pakistan)**						
Iqbal Hydrie et al. 2012	3	85	5	107	0.8%	0.75 [0.17, 3.22]
**Subtotal (95% CI)**		**85**		**107**	**0.8%**	**0.75 [0.17, 3.22]**
Total events	3		5			
Heterogeneity: Not applicable						
Test for overall effect: Z = 0.39 (P = 0.69)						
**1.2.4 Europe (Scotland, UK)**						
Mohan et al. 2019	0	31	1	32	0.2%	0.33 [0.01, 8.50]
Preiss et al. 2014	2	86	6	87	0.7%	0.32 [0.06, 1.64]
**Subtotal (95% CI)**		**117**		**119**	**0.9%**	**0.32 [0.08, 1.39]**
Total events	2		7			
Heterogeneity: Tau² = 0.00; Chi² = 0.00, df = 1 (P = 0.98); I² = 0%						
Test for overall effect: Z = 1.52 (P = 0.13)						
**1.2.5 Europe (Netherlands)**						
Lexis et al. 2015	20	162	18	156	3.9%	1.08 [0.55, 2.13]
Hartman et al. 2017	34	191	32	188	6.3%	1.06 [0.62, 1.80]
**Subtotal (95% CI)**		**353**		**344**	**10.2%**	**1.06 [0.70, 1.62]**
Total events	54		50			
Heterogeneity: Tau² = 0.00; Chi² = 0.00, df = 1 (P = 0.96); I² = 0%						
Test for overall effect: Z = 0.29 (P = 0.77)						
**1.2.6 Europe (France)**						
Fontbonne et al. 1996	0	164	5	160	0.2%	0.09 [0.00, 1.57]
**Subtotal (95% CI)**		**164**		**160**	**0.2%**	**0.09 [0.00, 1.57]**
Total events	0		5			
Heterogeneity: Not applicable						
Test for overall effect: Z = 1.66 (P = 0.10)						
**1.2.7 Europe (Finland)**						
Lehtovira et al. 2001	1	20	1	20	0.2%	1.00 [0.06, 17.18]
**Subtotal (95% CI)**		**20**		**20**	**0.2%**	**1.00 [0.06, 17.18]**
Total events	1		1			
Heterogeneity: Not applicable						
Test for overall effect: Z = 0.00 (P = 1.00)						
**1.2.8 North America (USA)**						
Knowler et al. 2002	225	893	304	910	38.5%	0.67 [0.55, 0.82]
**Subtotal (95% CI)**		**893**		**910**	**38.5%**	**0.67 [0.55, 0.82]**
Total events	225		304			
Heterogeneity: Not applicable						
Test for overall effect: Z = 3.82 (P = 0.0001)						
**1.2.9 Africa (Tanzania)**						
Garrib et al. 2023	42	145	59	158	7.6%	0.68 [0.42, 1.11]
**Subtotal (95% CI)**		**145**		**158**	**7.6%**	**0.68 [0.42, 1.11]**
Total events	42		59			
Heterogeneity: Not applicable						
Test for overall effect: Z = 1.54 (P = 0.12)						
**Total (95% CI)**		**2603**		**2726**	**100.0%**	**0.77 [0.67, 0.88]**
Total events	621		791			
Heterogeneity: Tau² = 0.00; Chi² = 12.22, df = 12 (P = 0.43); I² = 2%						
Test for overall effect: Z = 3.82 (P = 0.0001)						
Test for subgroup differences: Chi² = 9.02, df = 8 (P = 0.34), I² = 11.3%						

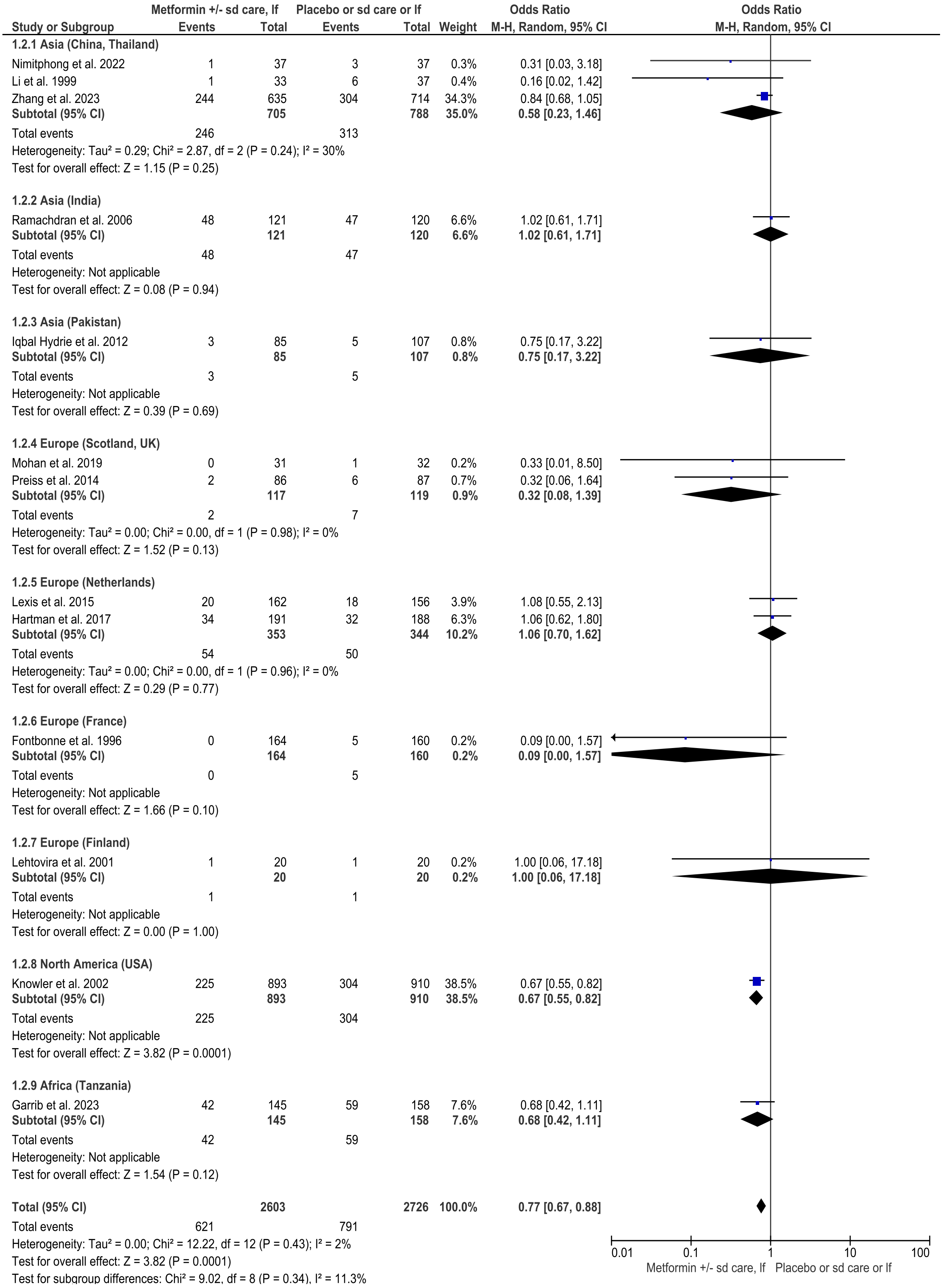

Sd, standard; lf, lifestyle; Events, number of participants with Type-2 Diabetes Mellitus; Total, number of participants at risk for Type-2 Diabetes Mellitus; Blue dots, weight of studies; Black blocks, 95% confidence interval of studies; Diamonds, estimates with 95% confidence interval.

Supplemental Figure S2. Subgroup Analysis for overall effectiveness of metformin based on gender predominance.

Study or Subgroup	Metformin +/- sd care, lf Events	Metformin +/- sd care, lf Total	Placebo or sd care or lf Events	Placebo or sd care or lf Total	Weight	Odds Ratio M-H, Random, 95% CI
**1.3.1 Men more than women**						
Lehtovira et al. 2001	1	20	1	20	0.2%	1.00 [0.06, 17.18]
Nimitphong et al. 2022	1	37	3	37	0.3%	0.31 [0.03, 3.18]
Li et al. 1999	1	33	6	37	0.4%	0.16 [0.02, 1.42]
Preiss et al. 2014	2	86	6	87	0.7%	0.32 [0.06, 1.64]
Iqbal Hydrie et al. 2012	3	85	5	107	0.8%	0.75 [0.17, 3.22]
Hartman et al. 2017	34	191	32	188	6.3%	1.06 [0.62, 1.80]
Ramachdran et al. 2006	48	121	47	120	6.6%	1.02 [0.61, 1.71]
Garrib et al. 2023	42	145	59	158	7.6%	0.68 [0.42, 1.11]
**Subtotal (95% CI)**		**718**		**754**	**23.0%**	**0.82 [0.62, 1.09]**
Total events	132		159			
Heterogeneity: Tau² = 0.00; Chi² = 6.23, df = 7 (P = 0.51); I² = 0%						
Test for overall effect: Z = 1.38 (P = 0.17)						
**1.3.2 Women more than men**						
Mohan et al. 2019	0	31	1	32	0.2%	0.33 [0.01, 8.50]
Fontbonne et al. 1996	0	164	5	160	0.2%	0.09 [0.00, 1.57]
Lexis et al. 2015	20	162	18	156	3.9%	1.08 [0.55, 2.13]
Zhang et al. 2023	244	635	304	714	34.3%	0.84 [0.68, 1.05]
Knowler et al. 2002	225	893	304	910	38.5%	0.67 [0.55, 0.82]
**Subtotal (95% CI)**		**1885**		**1972**	**77.0%**	**0.76 [0.61, 0.95]**
Total events	489		632			
Heterogeneity: Tau² = 0.02; Chi² = 5.69, df = 4 (P = 0.22); I² = 30%						
Test for overall effect: Z = 2.39 (P = 0.02)						
**Total (95% CI)**		**2603**		**2726**	**100.0%**	**0.77 [0.67, 0.88]**
Total events	621		791			
Heterogeneity: Tau² = 0.00; Chi² = 12.22, df = 12 (P = 0.43); I² = 2%						
Test for overall effect: Z = 3.82 (P = 0.0001)						
Test for subgroup differences: Chi² = 0.17, df = 1 (P = 0.68), I² = 0%						

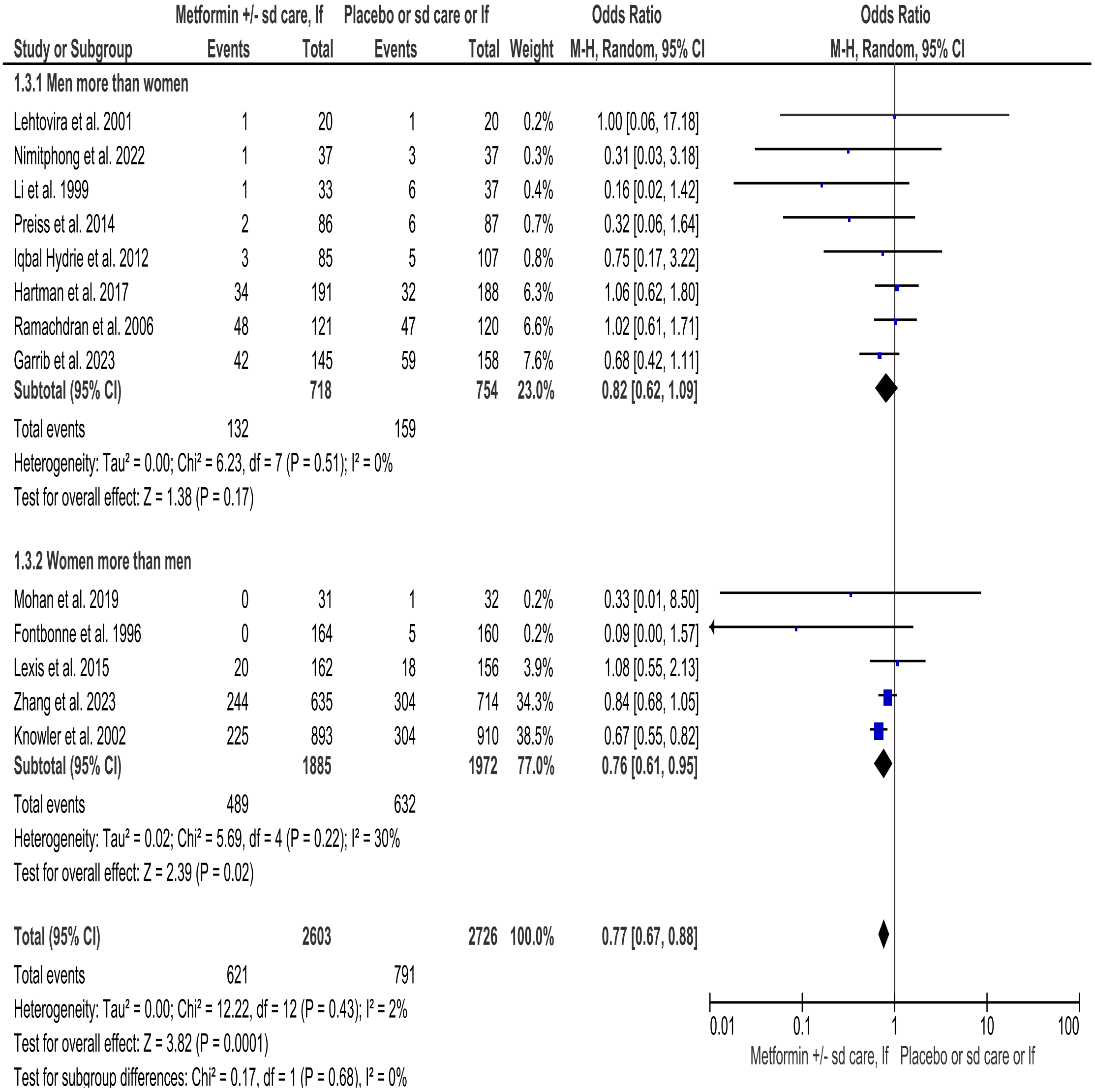

Sd, standard; lf, lifestyle; Events, number of participants with Type-2 Diabetes Mellitus; Total, number of participants at risk for Type-2 Diabetes Mellitus; Blue dots, weight of studies; Black blocks, 95% confidence interval of studies; Diamonds, estimates with 95% confidence interval.

Supplemental Figure S3. Subgroup Analysis for overall effectiveness of metformin based on participants' mean age.

Study or Subgroup	Metformin +/- sd care, lf Events	Metformin +/- sd care, lf Total	Placebo or sd care or lf Events	Placebo or sd care or lf Total	Weight	Odds Ratio M-H, Random, 95% CI
**1.4.1 Mean age more than 60 years**						
Mohan et al. 2019	0	31	1	32	0.2%	0.33 [0.01, 8.50]
Preiss et al. 2014	2	86	6	87	0.7%	0.32 [0.06, 1.64]
Knowler et al. 2002	225	893	304	910	38.5%	0.67 [0.55, 0.82]
**Subtotal (95% CI)**		**1010**		**1029**	**39.3%**	**0.66 [0.54, 0.81]**
Total events	227		311			
Heterogeneity: Tau² = 0.00; Chi² = 0.95, df = 2 (P = 0.62); I² = 0%						
Test for overall effect: Z = 3.99 (P < 0.0001)						
**1.4.2 Mean age less than 60 years**						
Fontbonne et al. 1996	0	164	5	160	0.2%	0.09 [0.00, 1.57]
Lehtovira et al. 2001	1	20	1	20	0.2%	1.00 [0.06, 17.18]
Nimitphong et al. 2022	1	37	3	37	0.3%	0.31 [0.03, 3.18]
Li et al. 1999	1	33	6	37	0.4%	0.16 [0.02, 1.42]
Iqbal Hydrie et al. 2012	3	85	5	107	0.8%	0.75 [0.17, 3.22]
Lexis et al. 2015	20	162	18	156	3.9%	1.08 [0.55, 2.13]
Hartman et al. 2017	34	191	32	188	6.3%	1.06 [0.62, 1.80]
Ramachdran et al. 2006	48	121	47	120	6.6%	1.02 [0.61, 1.71]
Garrib et al. 2023	42	145	59	158	7.6%	0.68 [0.42, 1.11]
Zhang et al. 2023	244	635	304	714	34.3%	0.84 [0.68, 1.05]
**Subtotal (95% CI)**		**1593**		**1697**	**60.7%**	**0.85 [0.72, 1.00]**
Total events	394		480			
Heterogeneity: Tau² = 0.00; Chi² = 7.81, df = 9 (P = 0.55); I² = 0%						
Test for overall effect: Z = 1.91 (P = 0.06)						
**Total (95% CI)**		**2603**		**2726**	**100.0%**	**0.77 [0.67, 0.88]**
Total events	621		791			
Heterogeneity: Tau² = 0.00; Chi² = 12.22, df = 12 (P = 0.43); I² = 2%						
Test for overall effect: Z = 3.82 (P = 0.0001)						
Test for subgroup differences: Chi² = 3.47, df = 1 (P = 0.06), I² = 71.2%						

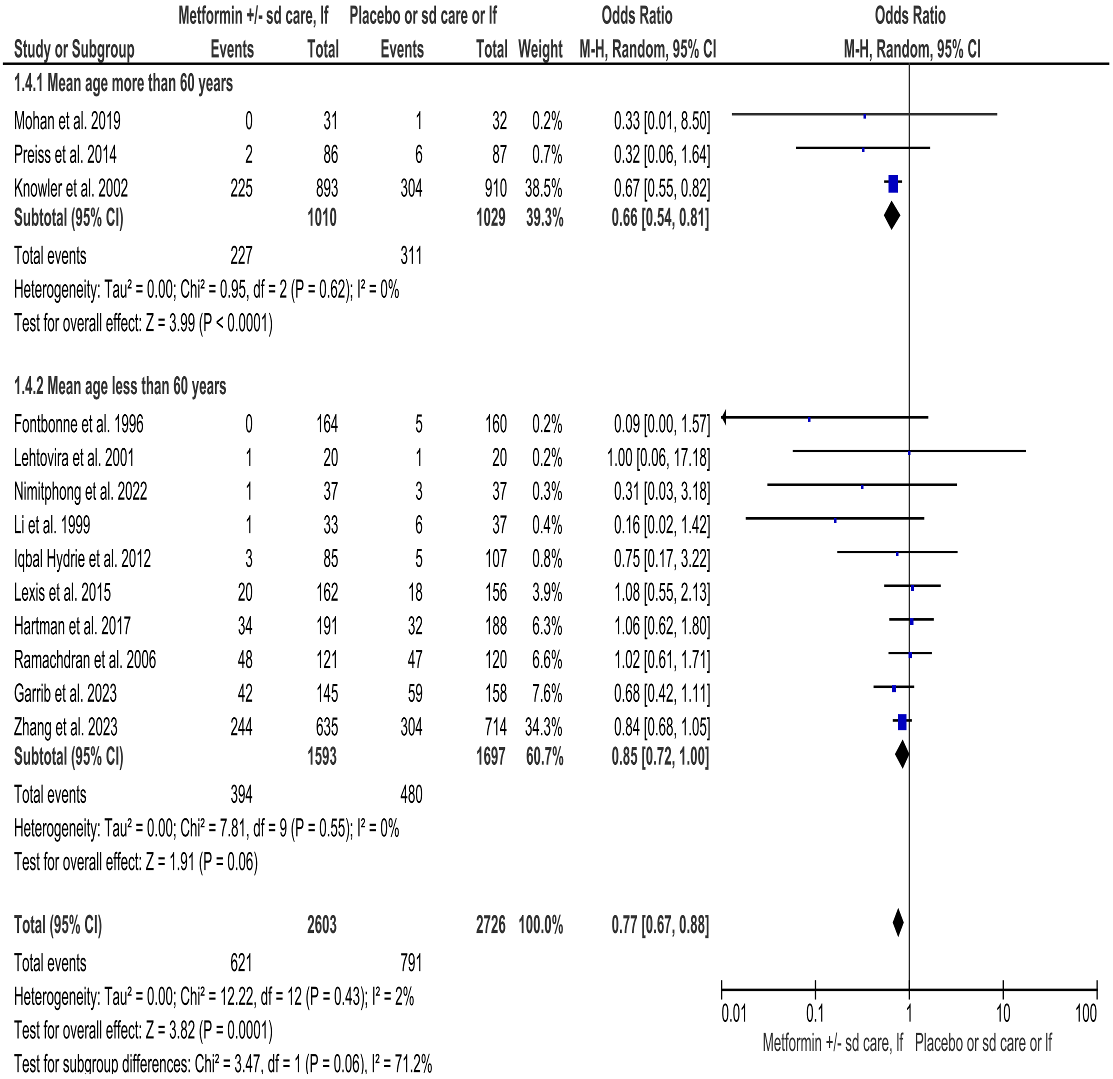

Sd, standard; lf, lifestyle; Events, number of participants with Type-2 Diabetes Mellitus; Total, number of participants at risk for Type-2 Diabetes Mellitus; Blue dots, weight of studies; Black blocks, 95% confidence interval of studies; Diamonds, estimates with 95% confidence interval.

Supplemental Figure S4. Subgroup Analysis for overall effectiveness of metformin based on prediabetes as diabetes' risk factor.

Study or Subgroup	Metformin +/- sd care, lf Events	Metformin +/- sd care, lf Total	Placebo or sd care or lf Events	Placebo or sd care or lf Total	Weight	Odds Ratio M-H, Random, 95% CI
**1.5.1 Prediabetes as main risk factor**						
Lehtovira et al. 2001	1	20	1	20	0.2%	1.00 [0.06, 17.18]
Nimitphong et al. 2022	1	37	3	37	0.3%	0.31 [0.03, 3.18]
Li et al. 1999	1	33	6	37	0.4%	0.16 [0.02, 1.42]
Iqbal Hydrie et al. 2012	3	85	5	107	0.8%	0.75 [0.17, 3.22]
Ramachdran et al. 2006	48	121	47	120	6.6%	1.02 [0.61, 1.71]
Garrib et al. 2023	42	145	59	158	7.6%	0.68 [0.42, 1.11]
Zhang et al. 2023	244	635	304	714	34.3%	0.84 [0.68, 1.05]
Knowler et al. 2002	225	893	304	910	38.5%	0.67 [0.55, 0.82]
**Subtotal (95% CI)**		**1969**		**2103**	**88.8%**	**0.75 [0.66, 0.86]**
Total events	565		729			
Heterogeneity: Tau² = 0.00; Chi² = 6.21, df = 7 (P = 0.52); I² = 0%						
Test for overall effect: Z = 4.12 (P < 0.0001)						
**1.5.2 No prediabetes as main risk factor**						
Mohan et al. 2019	0	31	1	32	0.2%	0.33 [0.01, 8.50]
Fontbonne et al. 1996	0	164	5	160	0.2%	0.09 [0.00, 1.57]
Preiss et al. 2014	2	86	6	87	0.7%	0.32 [0.06, 1.64]
Lexis et al. 2015	20	162	18	156	3.9%	1.08 [0.55, 2.13]
Hartman et al. 2017	34	191	32	188	6.3%	1.06 [0.62, 1.80]
**Subtotal (95% CI)**		**634**		**623**	**11.2%**	**0.85 [0.50, 1.45]**
Total events	56		62			
Heterogeneity: Tau² = 0.08; Chi² = 5.12, df = 4 (P = 0.28); I² = 22%						
Test for overall effect: Z = 0.60 (P = 0.55)						
**Total (95% CI)**		**2603**		**2726**	**100.0%**	**0.77 [0.67, 0.88]**
Total events	621		791			
Heterogeneity: Tau² = 0.00; Chi² = 12.22, df = 12 (P = 0.43); I² = 2%						
Test for overall effect: Z = 3.82 (P = 0.0001)						
Test for subgroup differences: Chi² = 0.19, df = 1 (P = 0.66), I² = 0%						

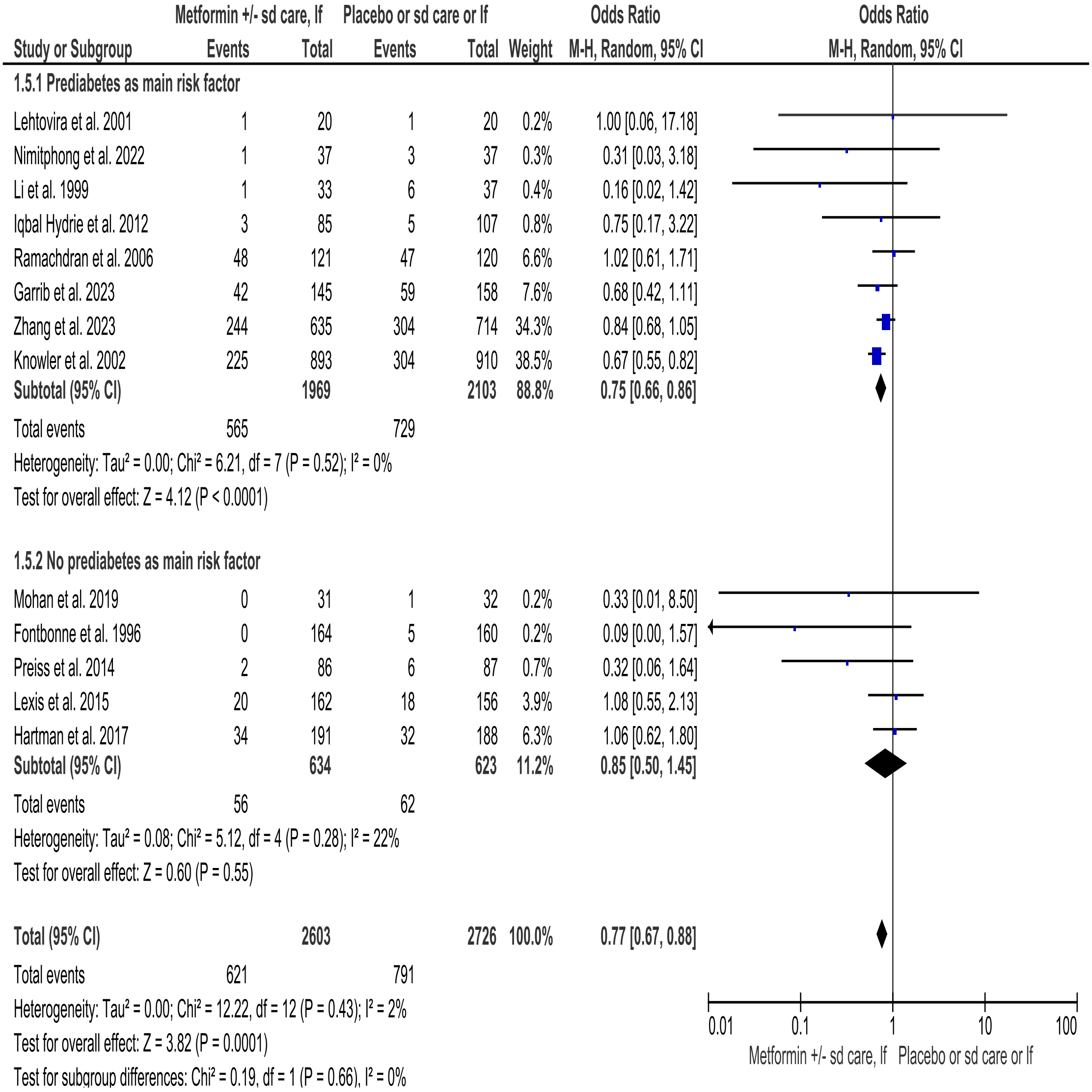

Sd, standard; lf, lifestyle; Events, number of participants with Type-2 Diabetes Mellitus; Total, number of participants at risk for Type-2 Diabetes Mellitus; Blue dots, weight of studies; Black blocks, 95% confidence interval of studies; Diamonds, estimates with 95% confidence interval.

Supplemental Figure S5. Subgroup Analysis for overall effectiveness of metformin based on overweight/obesity as diabetes' risk factor.

Study or Subgroup	Metformin +/- sd care, lf Events	Metformin +/- sd care, lf Total	Placebo or sd care or lf Events	Placebo or sd care or lf Total	Weight	Odds Ratio M-H, Random, 95% CI
**1.5.1 Prediabetes as main risk factor**						
Lehtovira et al. 2001	1	20	1	20	0.2%	1.00 [0.06, 17.18]
Nimitphong et al. 2022	1	37	3	37	0.3%	0.31 [0.03, 3.18]
Li et al. 1999	1	33	6	37	0.4%	0.16 [0.02, 1.42]
Iqbal Hydrie et al. 2012	3	85	5	107	0.8%	0.75 [0.17, 3.22]
Ramachdran et al. 2006	48	121	47	120	6.6%	1.02 [0.61, 1.71]
Garrib et al. 2023	42	145	59	158	7.6%	0.68 [0.42, 1.11]
Zhang et al. 2023	244	635	304	714	34.3%	0.84 [0.68, 1.05]
Knowler et al. 2002	225	893	304	910	38.5%	0.67 [0.55, 0.82]
**Subtotal (95% CI)**		**1969**		**2103**	**88.8%**	**0.75 [0.66, 0.86]**
Total events	565		729			
Heterogeneity: Tau² = 0.00; Chi² = 6.21, df = 7 (P = 0.52); I² = 0%						
Test for overall effect: Z = 4.12 (P < 0.0001)						
**1.5.2 No prediabetes as main risk factor**						
Mohan et al. 2019	0	31	1	32	0.2%	0.33 [0.01, 8.50]
Fontbonne et al. 1996	0	164	5	160	0.2%	0.09 [0.00, 1.57]
Preiss et al. 2014	2	86	6	87	0.7%	0.32 [0.06, 1.64]
Lexis et al. 2015	20	162	18	156	3.9%	1.08 [0.55, 2.13]
Hartman et al. 2017	34	191	32	188	6.3%	1.06 [0.62, 1.80]
**Subtotal (95% CI)**		**634**		**623**	**11.2%**	**0.85 [0.50, 1.45]**
Total events	56		62			
Heterogeneity: Tau² = 0.08; Chi² = 5.12, df = 4 (P = 0.28); I² = 22%						
Test for overall effect: Z = 0.60 (P = 0.55)						
**Total (95% CI)**		**2603**		**2726**	**100.0%**	**0.77 [0.67, 0.88]**
Total events	621		791			
Heterogeneity: Tau² = 0.00; Chi² = 12.22, df = 12 (P = 0.43); I² = 2%						
Test for overall effect: Z = 3.82 (P = 0.0001)						
Test for subgroup differences: Chi² = 0.19, df = 1 (P = 0.66), I² = 0%						

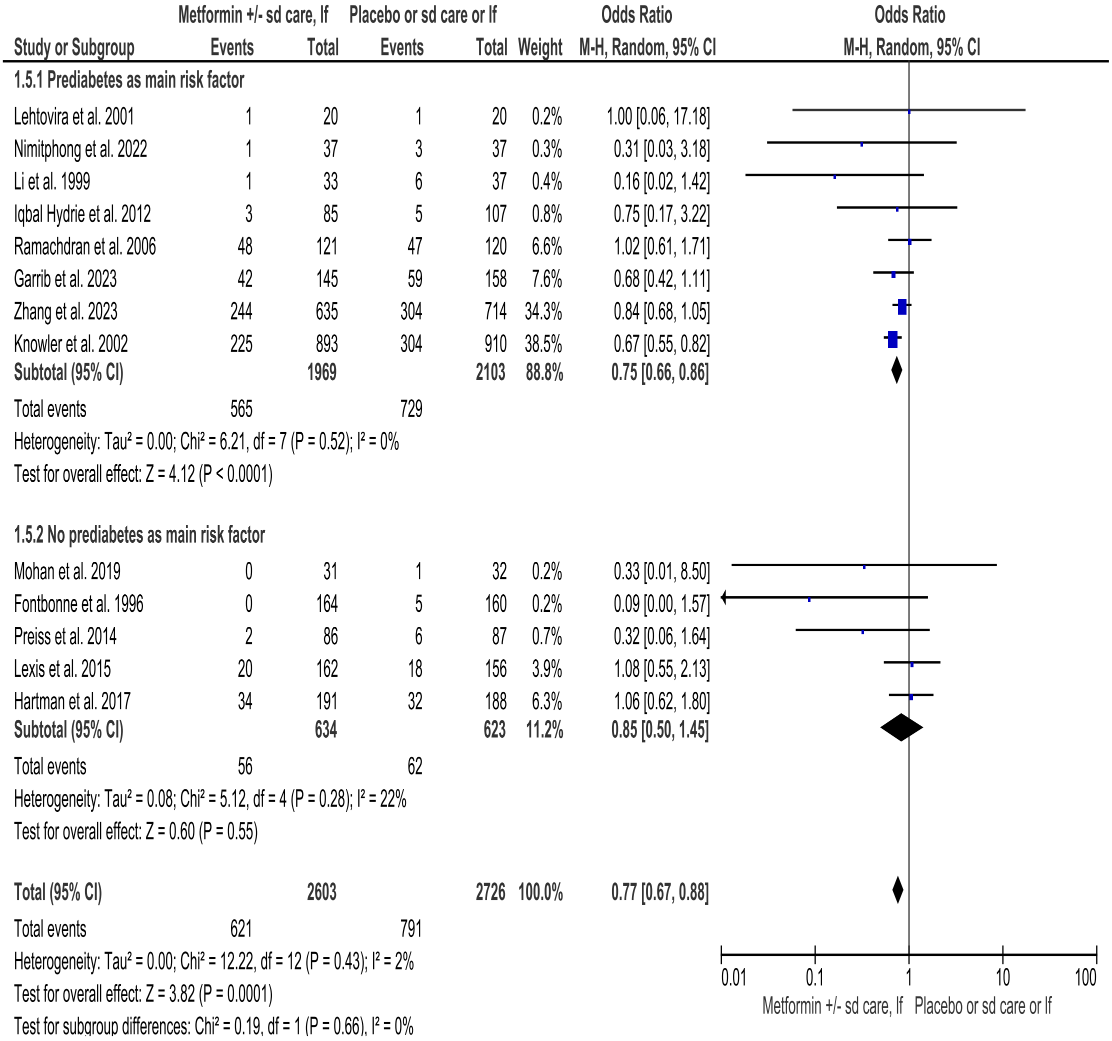

Sd, standard; lf, lifestyle; Events, number of participants with Type-2 Diabetes Mellitus; Total, number of participants at risk for Type-2 Diabetes Mellitus; Blue dots, weight of studies; Black blocks, 95% confidence interval of studies; Diamonds, estimates with 95% confidence interval.

Supplemental Figure S6. Subgroup Analysis for overall effectiveness of metformin based on cardiovascular disease as diabetes' risk factor.

	Metformin +/- sd care, lf		Placebo or sd care or lf			Odds Ratio
Study or Subgroup	Events	Total	Events	Total	Weight	M-H, Random, 95% CI
**1.5.1 Prediabetes as main risk factor**						
Lehtovira et al. 2001	1	20	1	20	0.2%	1.00 [0.06, 17.18]
Nimitphong et al. 2022	1	37	3	37	0.3%	0.31 [0.03, 3.18]
Li et al. 1999	1	33	6	37	0.4%	0.16 [0.02, 1.42]
Iqbal Hydrie et al. 2012	3	85	5	107	0.8%	0.75 [0.17, 3.22]
Ramachdran et al. 2006	48	121	47	120	6.6%	1.02 [0.61, 1.71]
Garrib et al. 2023	42	145	59	158	7.6%	0.68 [0.42, 1.11]
Zhang et al. 2023	244	635	304	714	34.3%	0.84 [0.68, 1.05]
Knowler et al. 2002	225	893	304	910	38.5%	0.67 [0.55, 0.82]
**Subtotal (95% CI)**		**1969**		**2103**	**88.8%**	**0.75 [0.66, 0.86]**
Total events	565		729			
Heterogeneity: Tau² = 0.00; Chi² = 6.21, df = 7 (P = 0.52); I² = 0%						
Test for overall effect: Z = 4.12 (P < 0.0001)						
**1.5.2 No prediabetes as main risk factor**						
Mohan et al. 2019	0	31	1	32	0.2%	0.33 [0.01, 8.50]
Fontbonne et al. 1996	0	164	5	160	0.2%	0.09 [0.00, 1.57]
Preiss et al. 2014	2	86	6	87	0.7%	0.32 [0.06, 1.64]
Lexis et al. 2015	20	162	18	156	3.9%	1.08 [0.55, 2.13]
Hartman et al. 2017	34	191	32	188	6.3%	1.06 [0.62, 1.80]
**Subtotal (95% CI)**		**634**		**623**	**11.2%**	**0.85 [0.50, 1.45]**
Total events	56		62			
Heterogeneity: Tau² = 0.08; Chi² = 5.12, df = 4 (P = 0.28); I² = 22%						
Test for overall effect: Z = 0.60 (P = 0.55)						
**Total (95% CI)**		**2603**		**2726**	**100.0%**	**0.77 [0.67, 0.88]**
Total events	621		791			
Heterogeneity: Tau² = 0.00; Chi² = 12.22, df = 12 (P = 0.43); I² = 2%						
Test for overall effect: Z = 3.82 (P = 0.0001)						
Test for subgroup differences: Chi² = 0.19, df = 1 (P = 0.66), I² = 0%						

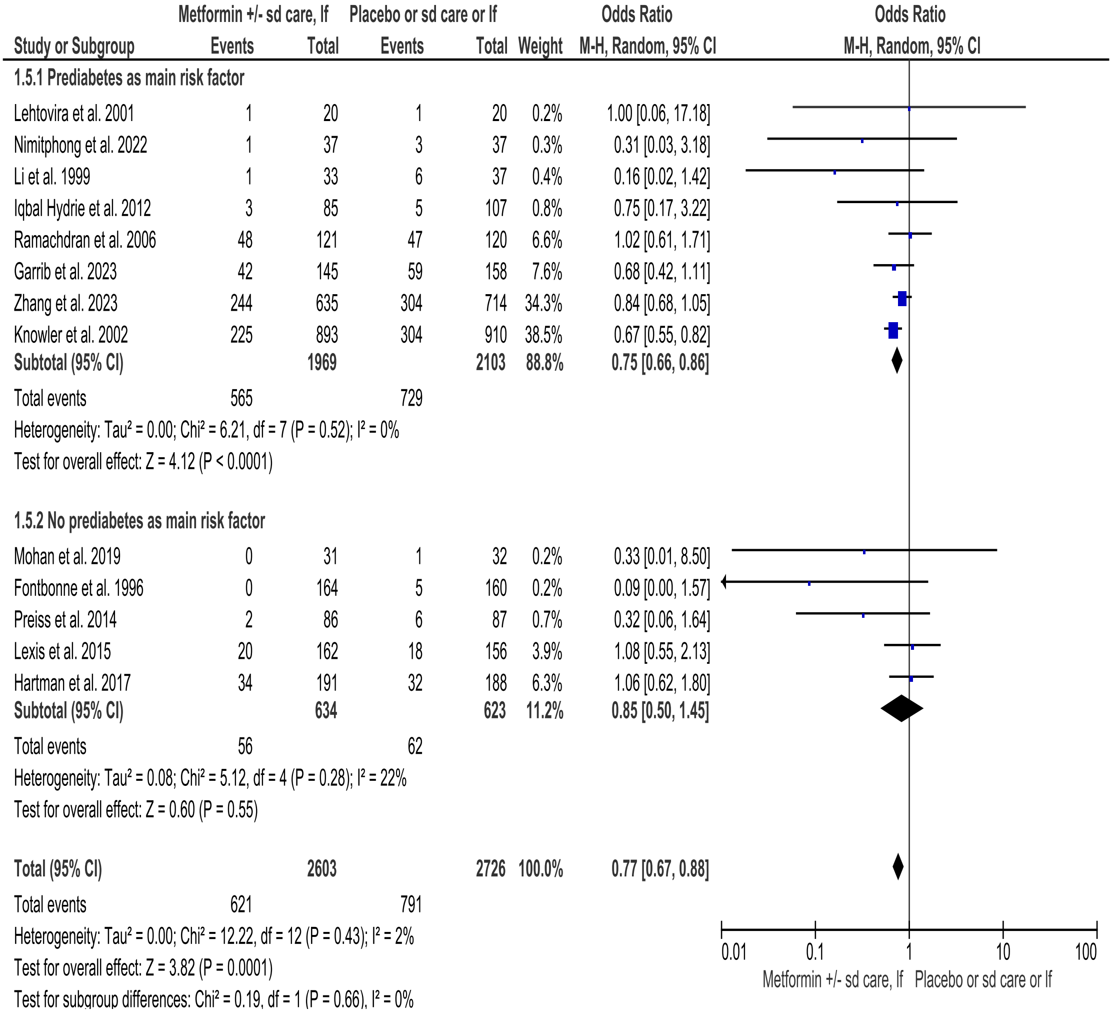

Sd, standard; lf, lifestyle; Events, number of participants with Type-2 Diabetes Mellitus; Total, number of participants at risk for Type-2 Diabetes Mellitus; Blue dots, weight of studies; Black blocks, 95% confidence interval of studies; Diamonds, estimates with 95% confidence interval.

Supplemental Figure S7. Subgroup Analysis for overall effectiveness of metformin based on Human Immunodeficiency Virus as diabetes' risk factor.

Study or Subgroup	Metformin +/- sd care, lf Events	Metformin +/- sd care, lf Total	Placebo or sd care or lf Events	Placebo or sd care or lf Total	Weight	Odds Ratio M-H, Random, 95% CI
**1.8.1 HIV as main risk factor**						
Nimitphong et al. 2022	1	37	3	37	0.3%	0.31 [0.03, 3.18]
Garrib et al. 2023	42	145	59	158	7.6%	0.68 [0.42, 1.11]
**Subtotal (95% CI)**		**182**		**195**	**7.9%**	**0.66 [0.41, 1.06]**
Total events	43		62			
Heterogeneity: Tau² = 0.00; Chi² = 0.42, df = 1 (P = 0.52); I² = 0%						
Test for overall effect: Z = 1.71 (P = 0.09)						
**1.8.2 No HIV as main risk factor**						
Mohan et al. 2019	0	31	1	32	0.2%	0.33 [0.01, 8.50]
Fontbonne et al. 1996	0	164	5	160	0.2%	0.09 [0.00, 1.57]
Lehtovira et al. 2001	1	20	1	20	0.2%	1.00 [0.06, 17.18]
Li et al. 1999	1	33	6	37	0.4%	0.16 [0.02, 1.42]
Preiss et al. 2014	2	86	6	87	0.7%	0.32 [0.06, 1.64]
Iqbal Hydrie et al. 2012	3	85	5	107	0.8%	0.75 [0.17, 3.22]
Lexis et al. 2015	20	162	18	156	3.9%	1.08 [0.55, 2.13]
Hartman et al. 2017	34	191	32	188	6.3%	1.06 [0.62, 1.80]
Ramachdran et al. 2006	48	121	47	120	6.6%	1.02 [0.61, 1.71]
Zhang et al. 2023	244	635	304	714	34.3%	0.84 [0.68, 1.05]
Knowler et al. 2002	225	893	304	910	38.5%	0.67 [0.55, 0.82]
**Subtotal (95% CI)**		**2421**		**2531**	**92.1%**	**0.79 [0.67, 0.94]**
Total events	578		729			
Heterogeneity: Tau² = 0.01; Chi² = 11.39, df = 10 (P = 0.33); I² = 12%						
Test for overall effect: Z = 2.68 (P = 0.007)						
**Total (95% CI)**		**2603**		**2726**	**100.0%**	**0.77 [0.67, 0.88]**
Total events	621		791			
Heterogeneity: Tau² = 0.00; Chi² = 12.22, df = 12 (P = 0.43); I² = 2%						
Test for overall effect: Z = 3.82 (P = 0.0001)						
Test for subgroup differences: Chi² = 0.48, df = 1 (P = 0.49), I² = 0%						

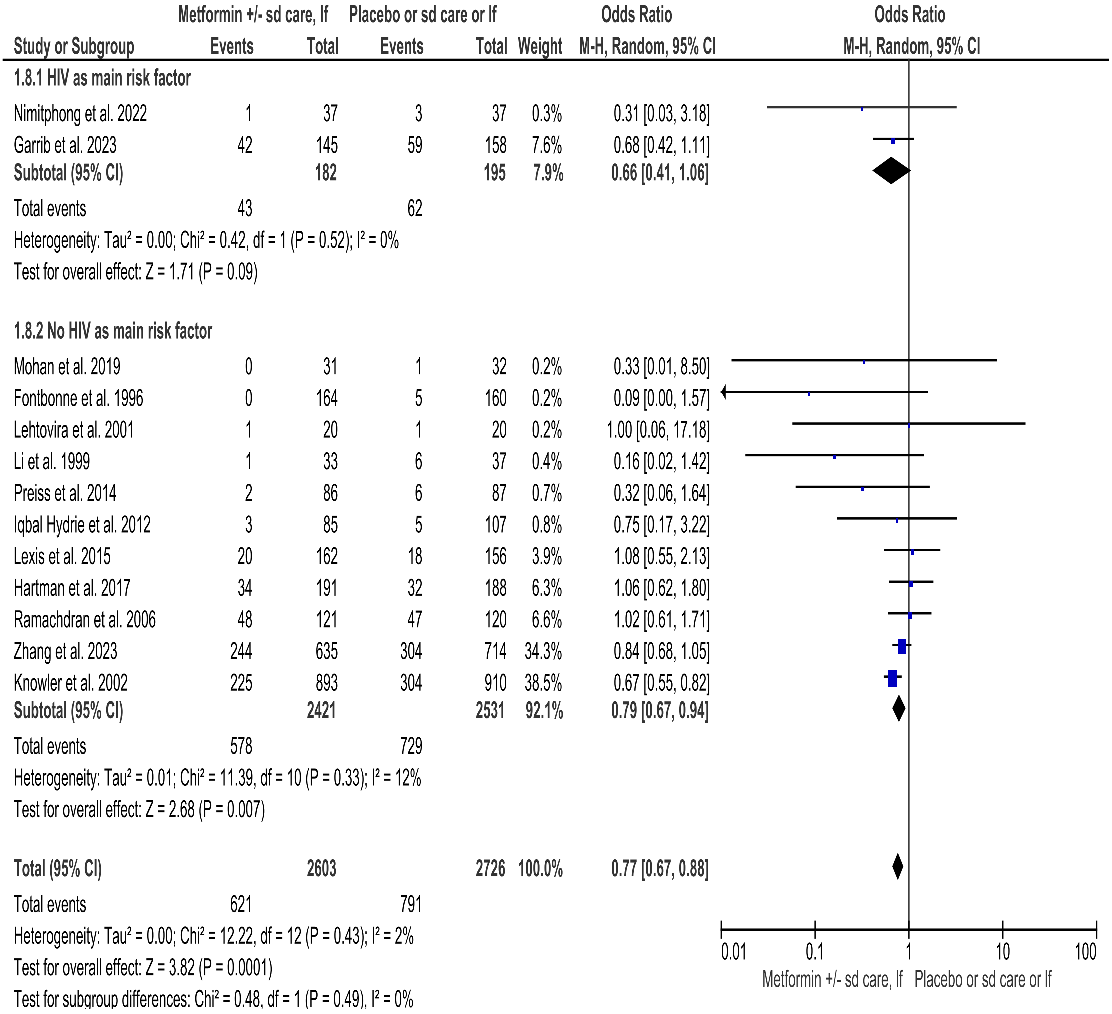

Sd, standard; lf, lifestyle; Events, number of participants with Type-2 Diabetes Mellitus; Total, number of participants at risk for Type-2 Diabetes Mellitus; Blue dots, weight of studies; Black blocks, 95% confidence interval of studies; Diamonds, estimates with 95% confidence interval.

Supplemental Figure S8. Subgroup Analysis for overall effectiveness of metformin based on metformin's daily dosage.

Study or Subgroup	Metformin +/- sd care, lf Events	Metformin +/- sd care, lf Total	Placebo or sd care or lf Events	Placebo or sd care or lf Total	Weight	Odds Ratio M-H, Random, 95% CI
**1.9.1 Metformin 500 mg daily**						
Ramachdran et al. 2006	48	121	47	120	6.6%	1.02 [0.61, 1.71]
**Subtotal (95% CI)**		**121**		**120**	**6.6%**	**1.02 [0.61, 1.71]**
Total events	48		47			
Heterogeneity: Not applicable						
Test for overall effect: Z = 0.08 (P = 0.94)						
**1.9.2 Metformin 750 mg daily**						
Li et al. 1999	1	33	6	37	0.4%	0.16 [0.02, 1.42]
**Subtotal (95% CI)**		**33**		**37**	**0.4%**	**0.16 [0.02, 1.42]**
Total events	1		6			
Heterogeneity: Not applicable						
Test for overall effect: Z = 1.64 (P = 0.10)						
**1.9.3 Metformin 1000 mg daily**						
Lehtovira et al. 2001	1	20	1	20	0.2%	1.00 [0.06, 17.18]
Nimitphong et al. 2022	1	37	3	37	0.3%	0.31 [0.03, 3.18]
Iqbal Hydrie et al. 2012	3	85	5	107	0.8%	0.75 [0.17, 3.22]
Lexis et al. 2015	20	162	18	156	3.9%	1.08 [0.55, 2.13]
Hartman et al. 2017	34	191	32	188	6.3%	1.06 [0.62, 1.80]
**Subtotal (95% CI)**		**495**		**508**	**11.6%**	**1.00 [0.68, 1.48]**
Total events	59		59			
Heterogeneity: Tau² = 0.00; Chi² = 1.21, df = 4 (P = 0.88); I² = 0%						
Test for overall effect: Z = 0.00 (P = 1.00)						
**1.9.4 Metformin 1700 mg daily**						
Fontbonne et al. 1996	0	164	5	160	0.2%	0.09 [0.00, 1.57]
Preiss et al. 2014	2	86	6	87	0.7%	0.32 [0.06, 1.64]
Zhang et al. 2023	244	635	304	714	34.3%	0.84 [0.68, 1.05]
Knowler et al. 2002	225	893	304	910	38.5%	0.67 [0.55, 0.82]
**Subtotal (95% CI)**		**1778**		**1871**	**73.7%**	**0.72 [0.55, 0.94]**
Total events	471		619			
Heterogeneity: Tau² = 0.03; Chi² = 5.33, df = 3 (P = 0.15); I² = 44%						
Test for overall effect: Z = 2.42 (P = 0.02)						
**1.9.5 Metformin 2000 mg daily**						
Mohan et al. 2019	0	31	1	32	0.2%	0.33 [0.01, 8.50]
Garrib et al. 2023	42	145	59	158	7.6%	0.68 [0.42, 1.11]
**Subtotal (95% CI)**		**176**		**190**	**7.8%**	**0.67 [0.42, 1.09]**
Total events	42		60			
Heterogeneity: Tau² = 0.00; Chi² = 0.19, df = 1 (P = 0.67); I² = 0%						
Test for overall effect: Z = 1.62 (P = 0.10)						
**Total (95% CI)**		**2603**		**2726**	**100.0%**	**0.77 [0.67, 0.88]**
Total events	621		791			
Heterogeneity: Tau² = 0.00; Chi² = 12.22, df = 12 (P = 0.43); I² = 2%						
Test for overall effect: Z = 3.82 (P = 0.0001)						
Test for subgroup differences: Chi² = 5.28, df = 4 (P = 0.26), I² = 24.2%						

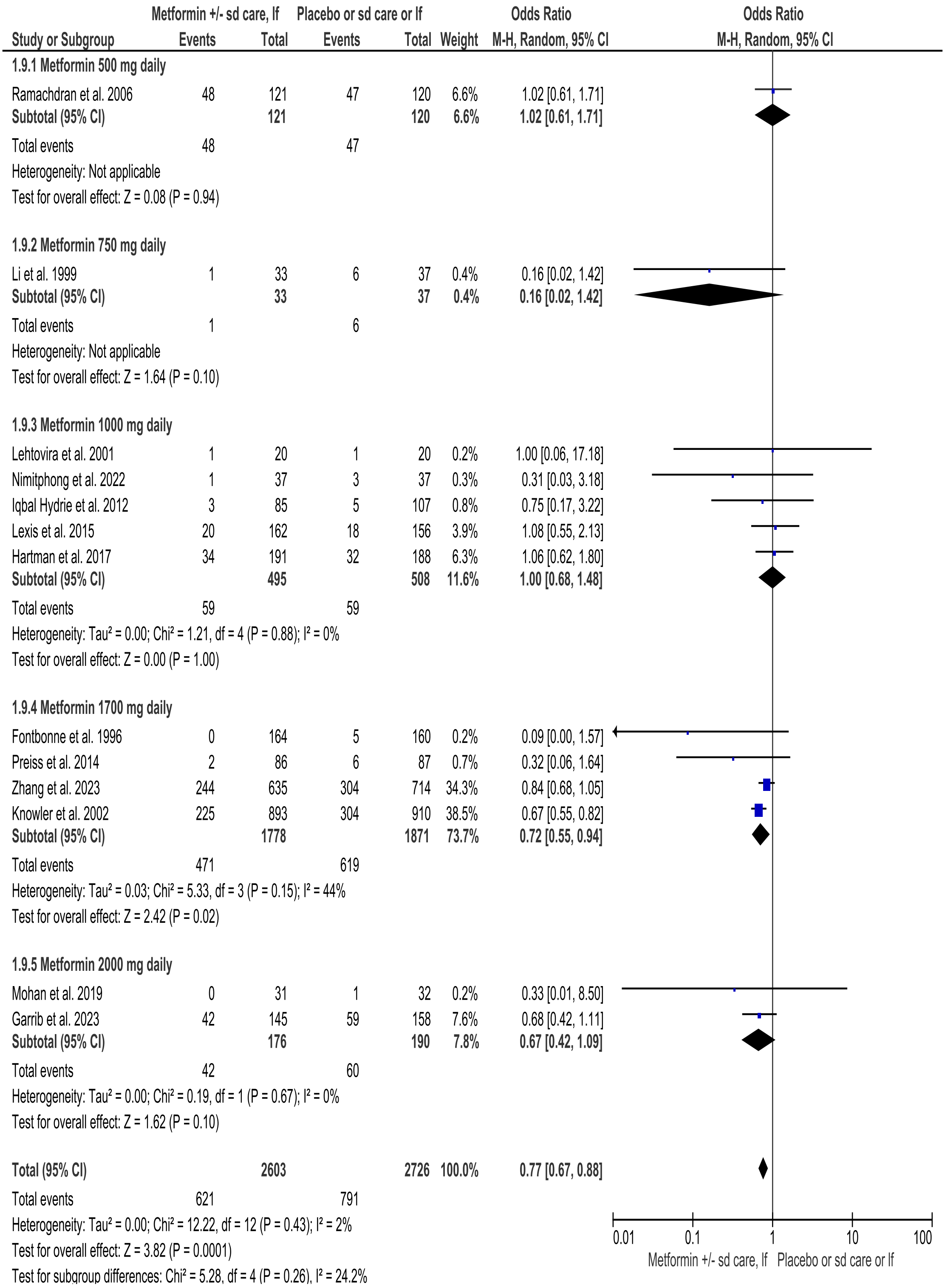

Sd, standard; lf, lifestyle; Events, number of participants with Type-2 Diabetes Mellitus; Total, number of participants at risk for Type-2 Diabetes Mellitus; Blue dots, weight of studies; Black blocks, 95% confidence interval of studies; Diamonds, estimates with 95% confidence interval.

Supplemental Figure S9. Subgroup Analysis for overall effectiveness of metformin based on metformin's intervention duration.

Study or Subgroup	Metformin +/- sd care, lf Events	Metformin +/- sd care, lf Total	Placebo or sd care or lf Events	Placebo or sd care or lf Total	Weight	Odds Ratio M-H, Random, 95% CI
**1.10.1 Intervention's duration more than 18 months**						
Ramachdran et al. 2006	48	121	47	120	6.6%	1.02 [0.61, 1.71]
Zhang et al. 2023	244	635	304	714	34.3%	0.84 [0.68, 1.05]
Knowler et al. 2002	225	893	304	910	38.5%	0.67 [0.55, 0.82]
**Subtotal (95% CI)**		**1649**		**1744**	**79.4%**	**0.78 [0.63, 0.96]**
Total events	517		655			
Heterogeneity: Tau² = 0.01; Chi² = 3.50, df = 2 (P = 0.17); I² = 43%						
Test for overall effect: Z = 2.34 (P = 0.02)						
**1.10.2 Intervention's up to 18 months**						
Mohan et al. 2019	0	31	1	32	0.2%	0.33 [0.01, 8.50]
Fontbonne et al. 1996	0	164	5	160	0.2%	0.09 [0.00, 1.57]
Lehtovira et al. 2001	1	20	1	20	0.2%	1.00 [0.06, 17.18]
Nimitphong et al. 2022	1	37	3	37	0.3%	0.31 [0.03, 3.18]
Li et al. 1999	1	33	6	37	0.4%	0.16 [0.02, 1.42]
Preiss et al. 2014	2	86	6	87	0.7%	0.32 [0.06, 1.64]
Iqbal Hydrie et al. 2012	3	85	5	107	0.8%	0.75 [0.17, 3.22]
Lexis et al. 2015	20	162	18	156	3.9%	1.08 [0.55, 2.13]
Hartman et al. 2017	34	191	32	188	6.3%	1.06 [0.62, 1.80]
Garrib et al. 2023	42	145	59	158	7.6%	0.68 [0.42, 1.11]
**Subtotal (95% CI)**		**954**		**982**	**20.6%**	**0.78 [0.58, 1.05]**
Total events	104		136			
Heterogeneity: Tau² = 0.00; Chi² = 8.77, df = 9 (P = 0.46); I² = 0%						
Test for overall effect: Z = 1.63 (P = 0.10)						
**Total (95% CI)**		**2603**		**2726**	**100.0%**	**0.77 [0.67, 0.88]**
Total events	621		791			
Heterogeneity: Tau² = 0.00; Chi² = 12.22, df = 12 (P = 0.43); I² = 2%						
Test for overall effect: Z = 3.82 (P = 0.0001)						
Test for subgroup differences: Chi² = 0.00, df = 1 (P = 0.99), I² = 0%						

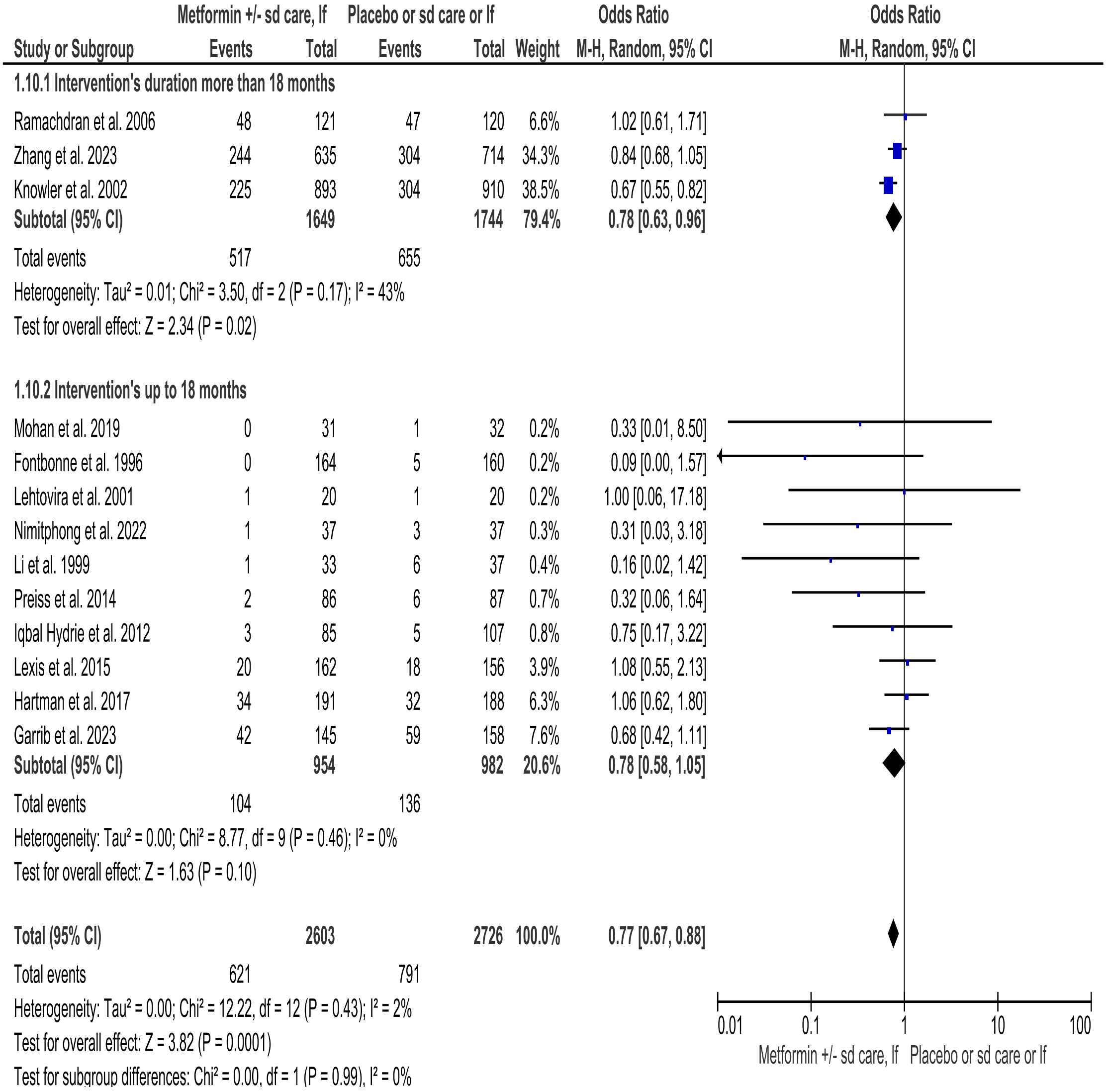

Sd, standard; lf, lifestyle; Events, number of participants with Type-2 Diabetes Mellitus; Total, number of participants at risk for Type-2 Diabetes Mellitus; Blue dots, weight of studies; Black blocks, 95% confidence interval of studies; Diamonds, estimates with 95% confidence interval.

Supplemental Figure S10. Subgroup Analysis for overall effectiveness of metformin based on metformin's post-intervention duration.

Study or Subgroup	Metformin +/- sd care, lf Events	Metformin +/- sd care, lf Total	Placebo or sd care or lf Events	Placebo or sd care or lf Total	Weight	Odds Ratio M-H, Random, 95% CI
**1.11.1 Post-intervention follow-up included**						
Hartman et al. 2017	34	191	32	188	6.3%	1.06 [0.62, 1.80]
**Subtotal (95% CI)**		**191**		**188**	**6.3%**	**1.06 [0.62, 1.80]**
Total events	34		32			
Heterogeneity: Not applicable						
Test for overall effect: Z = 0.20 (P = 0.84)						
**1.11.2 Post-intervention follow-up not included**						
Mohan et al. 2019	0	31	1	32	0.2%	0.33 [0.01, 8.50]
Fontbonne et al. 1996	0	164	5	160	0.2%	0.09 [0.00, 1.57]
Lehtovira et al. 2001	1	20	1	20	0.2%	1.00 [0.06, 17.18]
Nimitphong et al. 2022	1	37	3	37	0.3%	0.31 [0.03, 3.18]
Li et al. 1999	1	33	6	37	0.4%	0.16 [0.02, 1.42]
Preiss et al. 2014	2	86	6	87	0.7%	0.32 [0.06, 1.64]
Iqbal Hydrie et al. 2012	3	85	5	107	0.8%	0.75 [0.17, 3.22]
Lexis et al. 2015	20	162	18	156	3.9%	1.08 [0.55, 2.13]
Ramachdran et al. 2006	48	121	47	120	6.6%	1.02 [0.61, 1.71]
Garrib et al. 2023	42	145	59	158	7.6%	0.68 [0.42, 1.11]
Zhang et al. 2023	244	635	304	714	34.3%	0.84 [0.68, 1.05]
Knowler et al. 2002	225	893	304	910	38.5%	0.67 [0.55, 0.82]
**Subtotal (95% CI)**		**2412**		**2538**	**93.7%**	**0.75 [0.66, 0.86]**
Total events	587		759			
Heterogeneity: Tau² = 0.00; Chi² = 10.75, df = 11 (P = 0.46); I² = 0%						
Test for overall effect: Z = 4.19 (P < 0.0001)						
**Total (95% CI)**		**2603**		**2726**	**100.0%**	**0.77 [0.67, 0.88]**
Total events	621		791			
Heterogeneity: Tau² = 0.00; Chi² = 12.22, df = 12 (P = 0.43); I² = 2%						
Test for overall effect: Z = 3.82 (P = 0.0001)						
Test for subgroup differences: Chi² = 1.46, df = 1 (P = 0.23), I² = 31.7%						

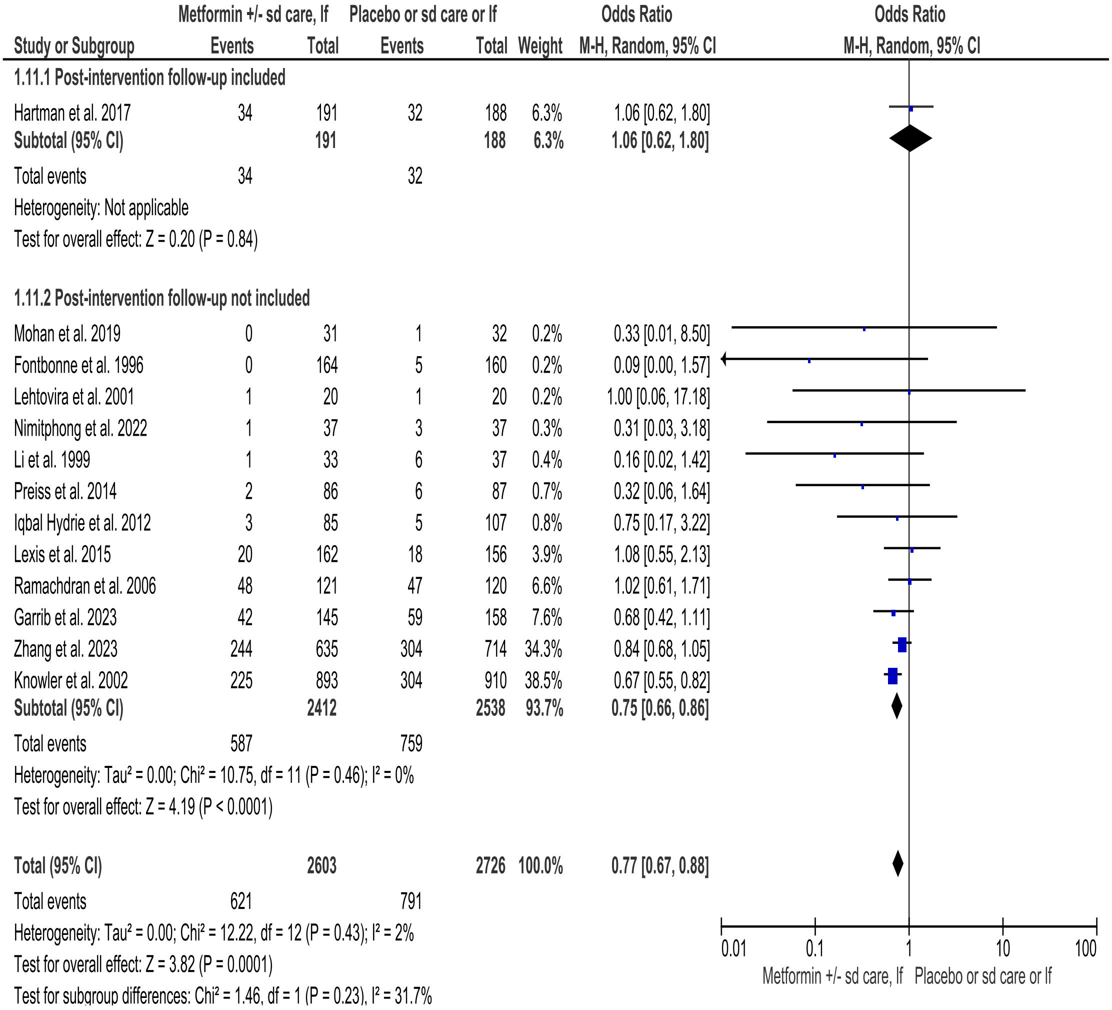

Sd, standard; lf, lifestyle; Events, number of participants with Type-2 Diabetes Mellitus; Total, number of participants at risk for Type-2 Diabetes Mellitus; Blue dots, weight of studies; Black blocks, 95% confidence interval of studies; Diamonds, estimates with 95% confidence interval.

Supplemental Figure S11. Subgroup Analysis for overall effectiveness of metformin based on diabetes' outcome assessment.

Study or Subgroup	Metformin +/- sd care, lf Events	Metformin +/- sd care, lf Total	Placebo or sd care or lf Events	Placebo or sd care or lf Total	Weight	Odds Ratio M-H, Random, 95% CI
**1.12.1 Type-2 Diabetes Mellitus as primary outcome**						
Nimitphong et al. 2022	1	37	3	37	0.3%	0.31 [0.03, 3.18]
Li et al. 1999	1	33	6	37	0.4%	0.16 [0.02, 1.42]
Iqbal Hydrie et al. 2012	3	85	5	107	0.8%	0.75 [0.17, 3.22]
Hartman et al. 2017	34	191	32	188	6.3%	1.06 [0.62, 1.80]
Ramachdran et al. 2006	48	121	47	120	6.6%	1.02 [0.61, 1.71]
Garrib et al. 2023	42	145	59	158	7.6%	0.68 [0.42, 1.11]
Zhang et al. 2023	244	635	304	714	34.3%	0.84 [0.68, 1.05]
Knowler et al. 2002	225	893	304	910	38.5%	0.67 [0.55, 0.82]
**Subtotal (95% CI)**		**2140**		**2271**	**94.8%**	**0.77 [0.66, 0.90]**
Total events	598		760			
Heterogeneity: Tau² = 0.00; Chi² = 7.66, df = 7 (P = 0.36); I² = 9%						
Test for overall effect: Z = 3.38 (P = 0.0007)						
**1.12.2 Type-2 Diabetes Mellitus as secondary outcome**						
Mohan et al. 2019	0	31	1	32	0.2%	0.33 [0.01, 8.50]
Fontbonne et al. 1996	0	164	5	160	0.2%	0.09 [0.00, 1.57]
Lehtovira et al. 2001	1	20	1	20	0.2%	1.00 [0.06, 17.18]
Preiss et al. 2014	2	86	6	87	0.7%	0.32 [0.06, 1.64]
Lexis et al. 2015	20	162	18	156	3.9%	1.08 [0.55, 2.13]
**Subtotal (95% CI)**		**463**		**455**	**5.2%**	**0.67 [0.30, 1.47]**
Total events	23		31			
Heterogeneity: Tau² = 0.15; Chi² = 4.68, df = 4 (P = 0.32); I² = 15%						
Test for overall effect: Z = 1.01 (P = 0.31)						
**Total (95% CI)**		**2603**		**2726**	**100.0%**	**0.77 [0.67, 0.88]**
Total events	621		791			
Heterogeneity: Tau² = 0.00; Chi² = 12.22, df = 12 (P = 0.43); I² = 2%						
Test for overall effect: Z = 3.82 (P = 0.0001)						
Test for subgroup differences: Chi² = 0.13, df = 1 (P = 0.72), I² = 0%						

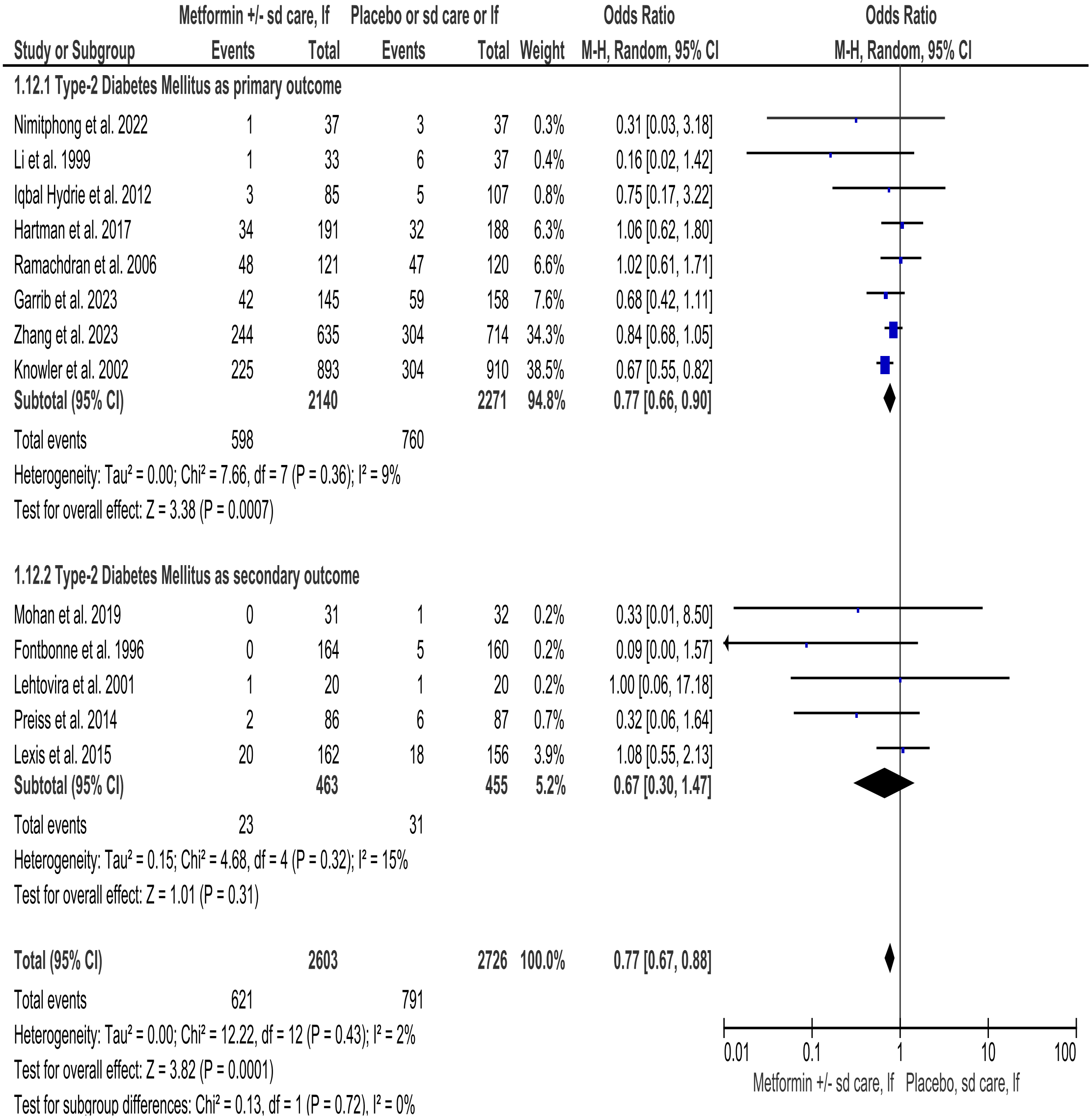

Sd, standard; lf, lifestyle; Events, number of participants with Type-2 Diabetes Mellitus; Total, number of participants at risk for Type-2 Diabetes Mellitus; Blue dots, weight of studies; Black blocks, 95% confidence interval of studies; Diamonds, estimates with 95% confidence interval.

Supplemental Figure S12. Subgroup Analysis for overall effectiveness of metformin based on studies' compared arms characteristics.

Study or Subgroup	Metformin +/- sd care, lf Events	Metformin +/- sd care, lf Total	Placebo or sd care or lf Events	Placebo or sd care or lf Total	Weight	Odds Ratio M-H, Random, 95% CI
**1.13.1 Metformin versus placebo**						
Mohan et al. 2019	0	31	1	32	0.2%	0.33 [0.01, 8.50]
Lehtovira et al. 2001	1	20	1	20	0.2%	1.00 [0.06, 17.18]
Li et al. 1999	1	33	6	37	0.4%	0.16 [0.02, 1.42]
Preiss et al. 2014	2	86	6	87	0.7%	0.32 [0.06, 1.64]
**Subtotal (95% CI)**		**170**		**176**	**1.5%**	**0.32 [0.11, 0.98]**
Total events	4		14			
Heterogeneity: Tau² = 0.00; Chi² = 1.00, df = 3 (P = 0.80); I² = 0%						
Test for overall effect: Z = 2.00 (P = 0.05)						
**1.13.2 Metformin plus sd care versus sd care**						
Fontbonne et al. 1996	0	164	5	160	0.2%	0.09 [0.00, 1.57]
Nimitphong et al. 2022	1	37	3	37	0.3%	0.31 [0.03, 3.18]
Lexis et al. 2015	20	162	18	156	3.9%	1.08 [0.55, 2.13]
Hartman et al. 2017	34	191	32	188	6.3%	1.06 [0.62, 1.80]
Garrib et al. 2023	42	145	59	158	7.6%	0.68 [0.42, 1.11]
Knowler et al. 2002	225	893	304	910	38.5%	0.67 [0.55, 0.82]
**Subtotal (95% CI)**		**1592**		**1609**	**56.8%**	**0.75 [0.58, 0.97]**
Total events	322		421			
Heterogeneity: Tau² = 0.02; Chi² = 6.42, df = 5 (P = 0.27); I² = 22%						
Test for overall effect: Z = 2.23 (P = 0.03)						
**1.13.3 Metformin plus lf intervention versus lf intervention**						
Iqbal Hydrie et al. 2012	3	85	5	107	0.8%	0.75 [0.17, 3.22]
Ramachdran et al. 2006	48	121	47	120	6.6%	1.02 [0.61, 1.71]
Zhang et al. 2023	244	635	304	714	34.3%	0.84 [0.68, 1.05]
**Subtotal (95% CI)**		**841**		**941**	**41.7%**	**0.86 [0.71, 1.05]**
Total events	295		356			
Heterogeneity: Tau² = 0.00; Chi² = 0.50, df = 2 (P = 0.78); I² = 0%						
Test for overall effect: Z = 1.44 (P = 0.15)						
**Total (95% CI)**		**2603**		**2726**	**100.0%**	**0.77 [0.67, 0.88]**
Total events	621		791			
Heterogeneity: Tau² = 0.00; Chi² = 12.22, df = 12 (P = 0.43); I² = 2%						
Test for overall effect: Z = 3.82 (P = 0.0001)						
Test for subgroup differences: Chi² = 3.41, df = 2 (P = 0.18), I² = 41.3%						

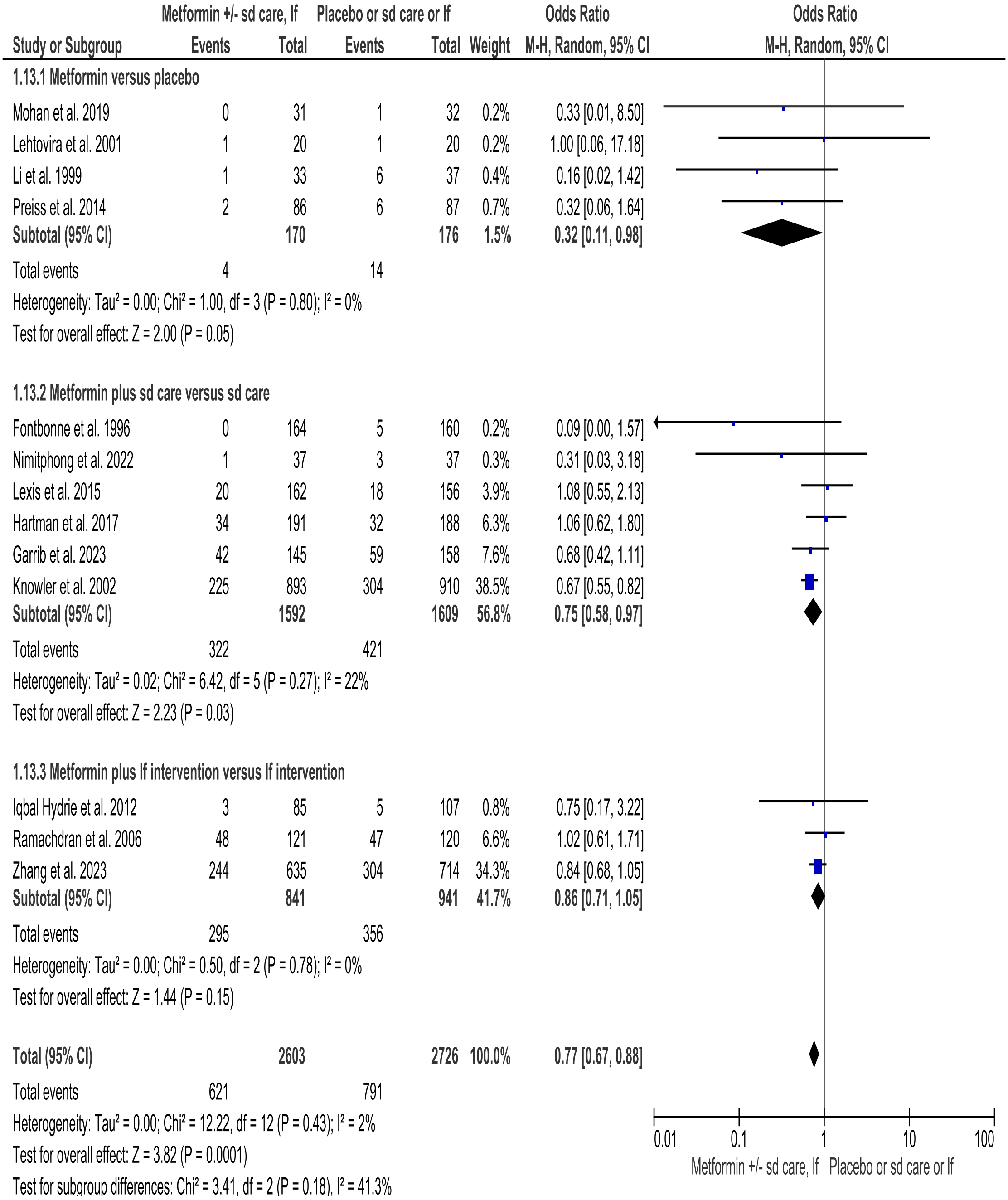

Sd, standard; lf, lifestyle; Events, number of participants with Type-2 Diabetes Mellitus; Total, number of participants at risk for Type-2 Diabetes Mellitus; Blue dots, weight of studies; Black blocks, 95% confidence interval of studies; Diamonds, estimates with 95% confidence interval.

Supplemental Figure S13. Subgroup Analysis for effectiveness of metformin and lifestyle interventions based on same performance countries.

	Metformin & lifestyle		Standard care			Odds Ratio
Study or Subgroup	Events	Total	Events	Total	Weight	M-H, Random, 95% CI
**1.2.1 Asia (India)**						
Weber et al. 2016	23	104	24	75	35.0%	0.60 [0.31, 1.18]
Ramachandran et al. 2006	48	121	73	133	50.4%	0.54 [0.33, 0.89]
**Subtotal (95% CI)**		**225**		**208**	**85.4%**	**0.56 [0.38, 0.84]**
Total events	71		97			
Heterogeneity: $Tau^2 = 0.00$; $Chi^2 = 0.07$, df = 1 (P = 0.80); $I^2 = 0\%$						
Test for overall effect: Z = 2.82 (P = 0.005)						
**1.2.2 Asia (China)**						
Lu et al. 2011	0	46	5	41	2.6%	0.07 [0.00, 1.33]
**Subtotal (95% CI)**		**46**		**41**	**2.6%**	**0.07 [0.00, 1.33]**
Total events	0		5			
Heterogeneity: Not applicable						
Test for overall effect: Z = 1.77 (P = 0.08)						
**1.2.3 Asia (Pakistan)**						
Iqbal Hydrie et al. 2012	3	85	12	82	12.0%	0.21 [0.06, 0.79]
**Subtotal (95% CI)**		**85**		**82**	**12.0%**	**0.21 [0.06, 0.79]**
Total events	3		12			
Heterogeneity: Not applicable						
Test for overall effect: Z = 2.32 (P = 0.02)						
**Total (95% CI)**		**356**		**331**	**100.0%**	**0.48 [0.30, 0.77]**
Total events	74		114			
Heterogeneity: $Tau^2 = 0.05$; $Chi^2 = 3.79$, df = 3 (P = 0.28); $I^2 = 21\%$						
Test for overall effect: Z = 3.04 (P = 0.002)						
Test for subgroup differences: $Chi^2 = 3.66$, df = 2 (P = 0.16), $I^2 = 45.4\%$						

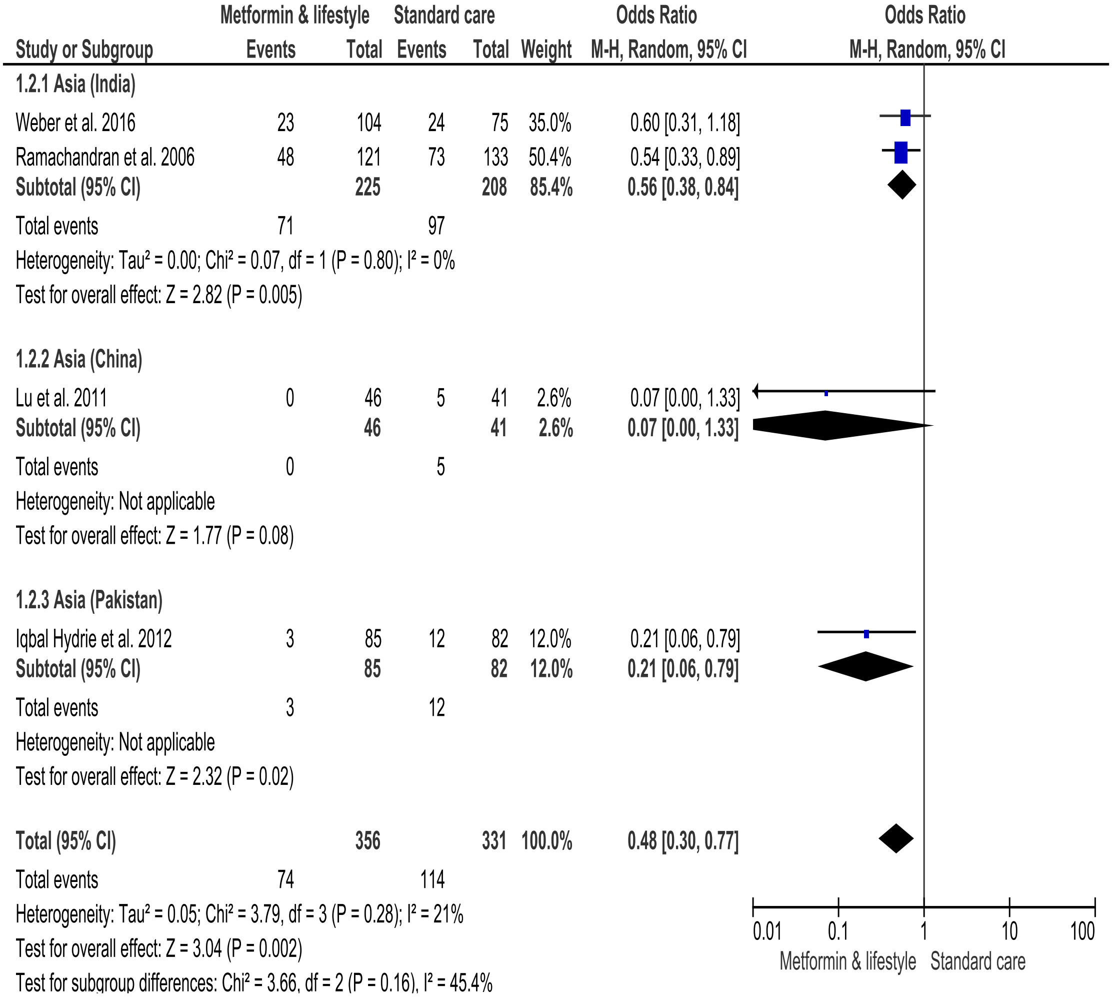

Events, number of participants with Type-2 Diabetes Mellitus; Total, number of participants at risk for Type-2 Diabetes Mellitus; Blue dots, weight of studies; Black blocks, 95% confidence interval of studies; Diamonds, estimates with 95% confidence interval.

Supplemental Figure S14. Subgroup Analysis for effectiveness of metformin and lifestyle interventions based on participants' mean age.

Study or Subgroup	Metformin & lifestyle Events	Metformin & lifestyle Total	Standard care Events	Standard care Total	Weight	Odds Ratio M-H, Random, 95% CI
**1.3.1 Mean age more than 60 years**						
Lu et al. 2011	0	46	5	41	2.6%	0.07 [0.00, 1.33]
**Subtotal (95% CI)**		**46**		**41**	**2.6%**	**0.07 [0.00, 1.33]**
Total events	0		5			
Heterogeneity: Not applicable						
Test for overall effect: Z = 1.77 (P = 0.08)						
**1.3.2 Mean age less than 60 years**						
Iqbal Hydrie et al. 2012	3	85	12	82	12.0%	0.21 [0.06, 0.79]
Weber et al. 2016	23	104	24	75	35.0%	0.60 [0.31, 1.18]
Ramachandran et al. 2006	48	121	73	133	50.4%	0.54 [0.33, 0.89]
**Subtotal (95% CI)**		**310**		**290**	**97.4%**	**0.52 [0.35, 0.76]**
Total events	74		109			
Heterogeneity: Tau² = 0.00; Chi² = 2.01, df = 2 (P = 0.37); I² = 1%						
Test for overall effect: Z = 3.36 (P = 0.0008)						
**Total (95% CI)**		**356**		**331**	**100.0%**	**0.48 [0.30, 0.77]**
Total events	74		114			
Heterogeneity: Tau² = 0.05; Chi² = 3.79, df = 3 (P = 0.28); I² = 21%						
Test for overall effect: Z = 3.04 (P = 0.002)						
Test for subgroup differences: Chi² = 1.73, df = 1 (P = 0.19), I² = 42.1%						

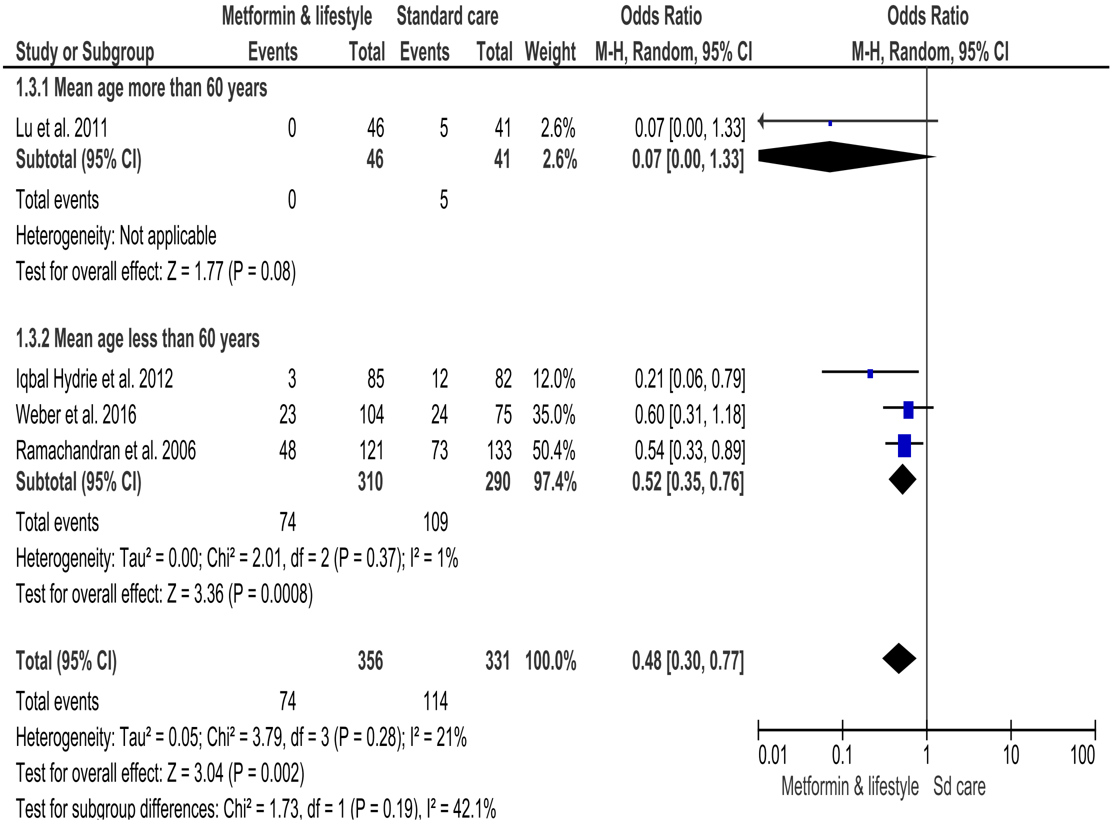

Events, number of participants with Type-2 Diabetes Mellitus; Total, number of participants at risk for Type-2 Diabetes Mellitus; Blue dots, weight of studies; Black blocks, 95% confidence interval of studies; Diamonds, estimates with 95% confidence interval.

Supplemental Figure S15. Subgroup Analysis for effectiveness of metformin and lifestyle interventions based on metformin's daily dosage.

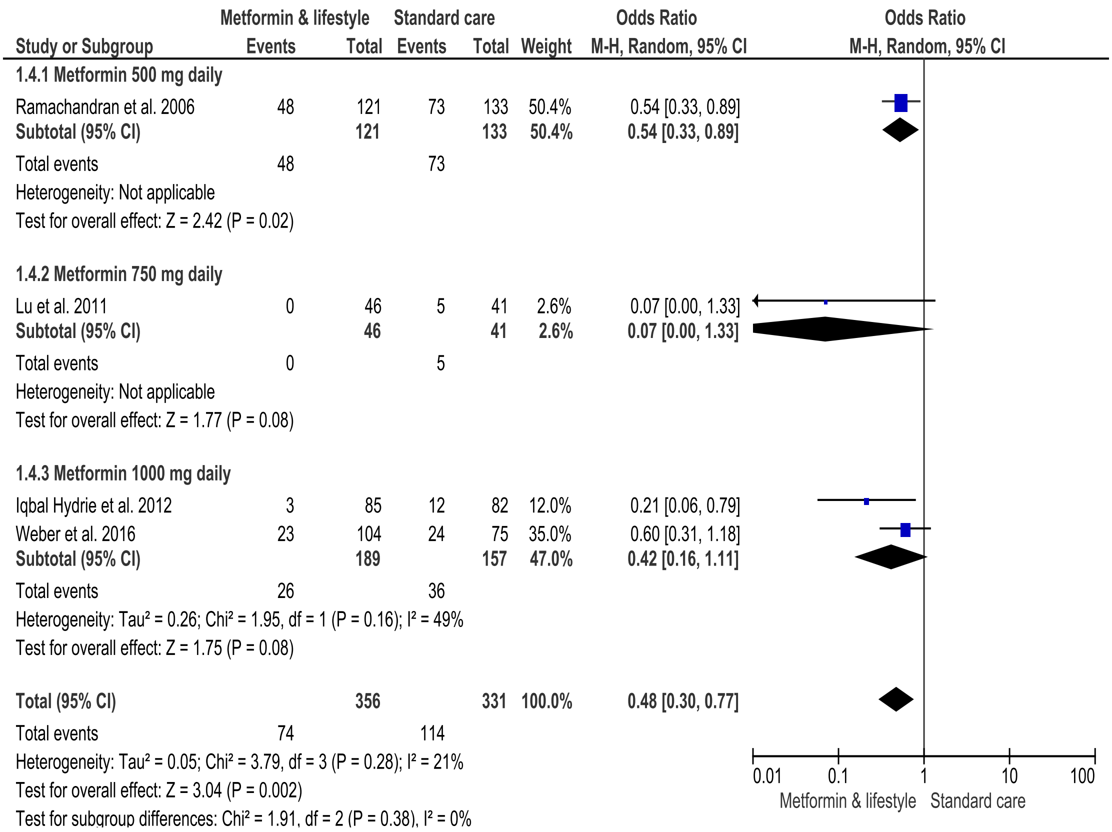

Events, number of participants with Type-2 Diabetes Mellitus; Total, number of participants at risk for Type-2 Diabetes Mellitus; Blue dots, weight of studies; Black blocks, 95% confidence interval of studies; Diamonds, estimates with 95% confidence interval.

Supplemental Figure S16. Subgroup Analysis for effectiveness of metformin and lifestyle interventions based on metformin's intervention duration.

	Metformin & lifestyle		Standard care			Odds Ratio
Study or Subgroup	Events	Total	Events	Total	Weight	M-H, Random, 95% CI
**1.5.1 Intervention's duration more than 6 months**						
Lu et al. 2011	0	46	5	41	2.6%	0.07 [0.00, 1.33]
Iqbal Hydrie et al. 2012	3	85	12	82	12.0%	0.21 [0.06, 0.79]
Ramachandran et al. 2006	48	121	73	133	50.4%	0.54 [0.33, 0.89]
**Subtotal (95% CI)**		**252**		**256**	**65.0%**	**0.34 [0.14, 0.86]**
Total events	51		90			
Heterogeneity: Tau² = 0.29; Chi² = 3.36, df = 2 (P = 0.19); I² = 40%						
Test for overall effect: Z = 2.29 (P = 0.02)						
**1.5.2 Intervention's duration up to 6 months**						
Weber et al. 2016	23	104	24	75	35.0%	0.60 [0.31, 1.18]
**Subtotal (95% CI)**		**104**		**75**	**35.0%**	**0.60 [0.31, 1.18]**
Total events	23		24			
Heterogeneity: Not applicable						
Test for overall effect: Z = 1.48 (P = 0.14)						
**Total (95% CI)**		**356**		**331**	**100.0%**	**0.48 [0.30, 0.77]**
Total events	74		114			
Heterogeneity: Tau² = 0.05; Chi² = 3.79, df = 3 (P = 0.28); I² = 21%						
Test for overall effect: Z = 3.04 (P = 0.002)						
Test for subgroup differences: Chi² = 0.94, df = 1 (P = 0.33), I² = 0%						

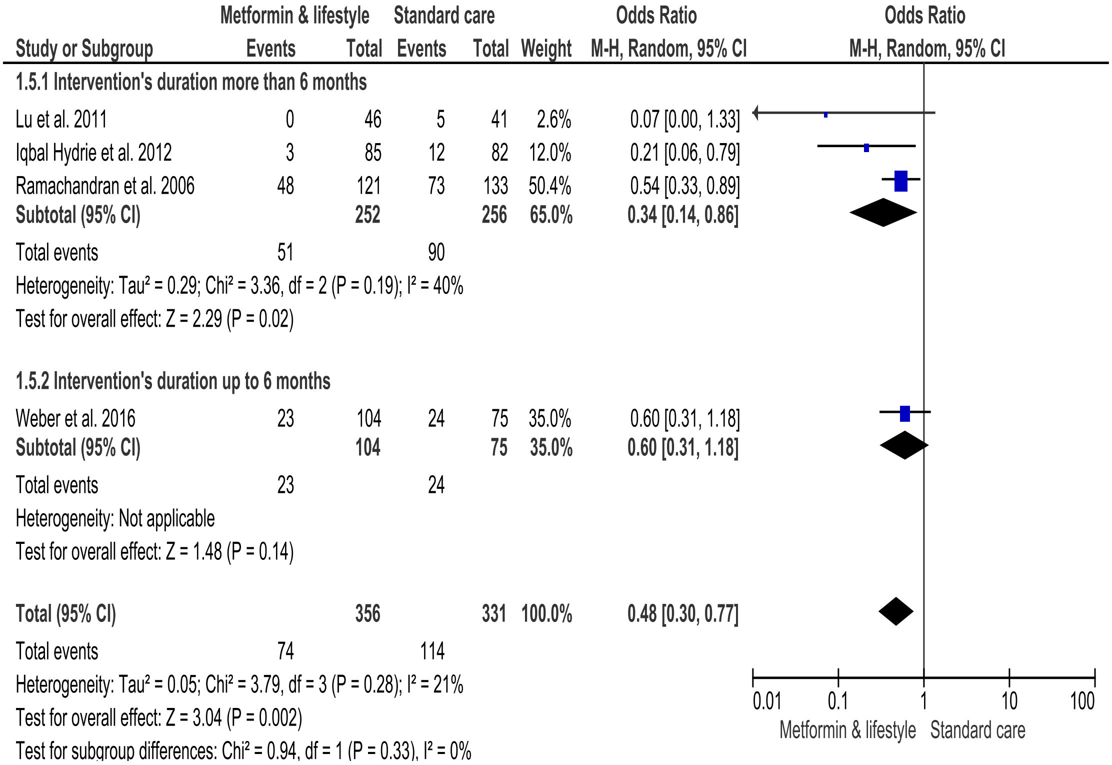

Events, number of participants with Type-2 Diabetes Mellitus; Total, number of participants at risk for Type-2 Diabetes Mellitus; Blue dots, weight of studies; Black blocks, 95% confidence interval of studies; Diamonds, estimates with 95% confidence interval.

Supplemental Figure S17. Subgroup Analysis for effectiveness of metformin and lifestyle interventions based on metformin's post-intervention duration.

Study or Subgroup	Metformin & lifestyle Events	Metformin & lifestyle Total	Standard care Events	Standard care Total	Weight	Odds Ratio M-H, Random, 95% CI
**1.6.1 Post-intervention follow-up included**						
Weber et al. 2016	23	104	24	75	35.0%	0.60 [0.31, 1.18]
**Subtotal (95% CI)**		**104**		**75**	**35.0%**	**0.60 [0.31, 1.18]**
Total events	23		24			
Heterogeneity: Not applicable						
Test for overall effect: Z = 1.48 (P = 0.14)						
**1.6.2 Post-intervention follow-up not included**						
Lu et al. 2011	0	46	5	41	2.6%	0.07 [0.00, 1.33]
Iqbal Hydrie et al. 2012	3	85	12	82	12.0%	0.21 [0.06, 0.79]
Ramachandran et al. 2006	48	121	73	133	50.4%	0.54 [0.33, 0.89]
**Subtotal (95% CI)**		**252**		**256**	**65.0%**	**0.34 [0.14, 0.86]**
Total events	51		90			
Heterogeneity: Tau² = 0.29; Chi² = 3.36, df = 2 (P = 0.19); I² = 40%						
Test for overall effect: Z = 2.29 (P = 0.02)						
**Total (95% CI)**		**356**		**331**	**100.0%**	**0.48 [0.30, 0.77]**
Total events	74		114			
Heterogeneity: Tau² = 0.05; Chi² = 3.79, df = 3 (P = 0.28); I² = 21%						
Test for overall effect: Z = 3.04 (P = 0.002)						
Test for subgroup differences: Chi² = 0.94, df = 1 (P = 0.33), I² = 0%						

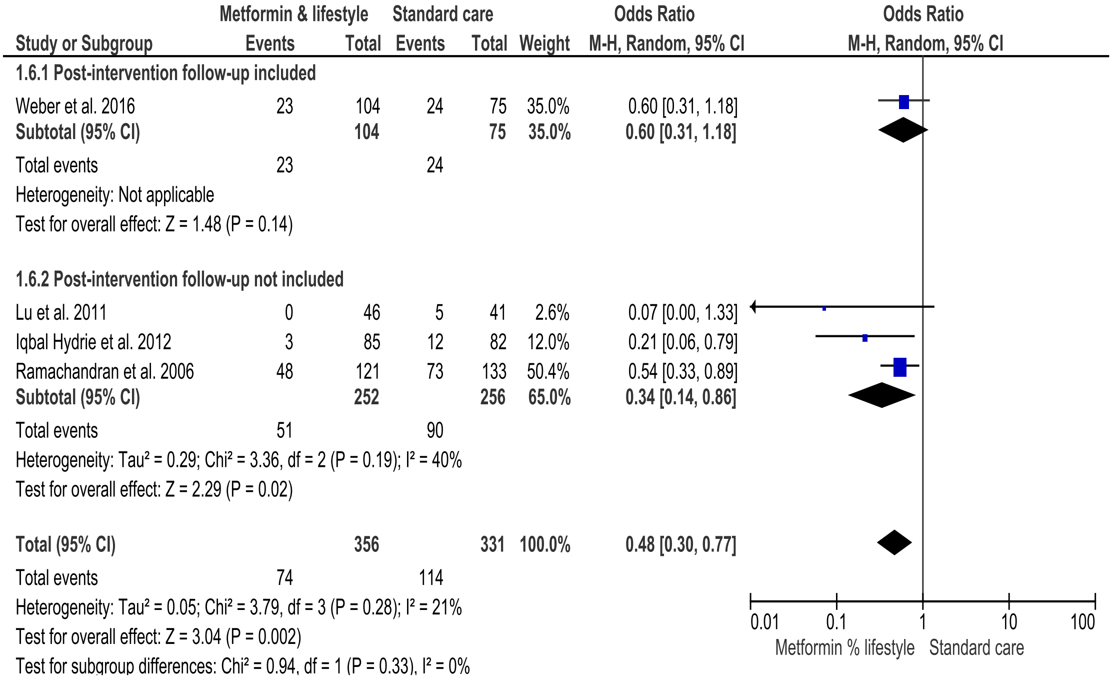

Events, number of participants with Type-2 Diabetes Mellitus; Total, number of participants at risk for Type-2 Diabetes Mellitus; Blue dots, weight of studies; Black blocks, 95% confidence interval of studies; Diamonds, estimates with 95% confidence interval.

Supplemental Figure S18. Sensitivity Analysis for RCT with the largest sample size in overall effectiveness of metformin.

	Metformin +/- sd care, lf		Placebo or sd care or lf			Odds Ratio
Study or Subgroup	Events	Total	Events	Total	Weight	M-H, Random, 95% CI
Mohan et al. 2019	0	31	1	32	0.3%	0.33 [0.01, 8.50]
Fontbonne et al. 1996	0	164	5	160	0.3%	0.09 [0.00, 1.57]
Lehtovira et al. 2001	1	20	1	20	0.3%	1.00 [0.06, 17.18]
Nimitphong et al. 2022	1	37	3	37	0.5%	0.31 [0.03, 3.18]
Li et al. 1999	1	33	6	37	0.6%	0.16 [0.02, 1.42]
Preiss et al. 2014	2	86	6	87	1.0%	0.32 [0.06, 1.64]
Iqbal Hydrie et al. 2012	3	85	5	107	1.3%	0.75 [0.17, 3.22]
Lexis et al. 2015	20	162	18	156	6.0%	1.08 [0.55, 2.13]
Hartman et al. 2017	34	191	32	188	9.7%	1.06 [0.62, 1.80]
Ramachdran et al. 2006	48	121	47	120	10.3%	1.02 [0.61, 1.71]
Garrib et al. 2023	42	145	59	158	11.8%	0.68 [0.42, 1.11]
Knowler et al. 2002	225	893	304	910	0.0%	0.67 [0.55, 0.82]
Zhang et al. 2023	244	635	304	714	57.8%	0.84 [0.68, 1.05]
**Total (95% CI)**		**1710**		**1816**	**100.0%**	**0.84 [0.71, 0.99]**
Total events	396		487			

Heterogeneity: Tau² = 0.00; Chi² = 9.48, df = 11 (P = 0.58); I² = 0%

Test for overall effect: Z = 2.07 (P = 0.04)

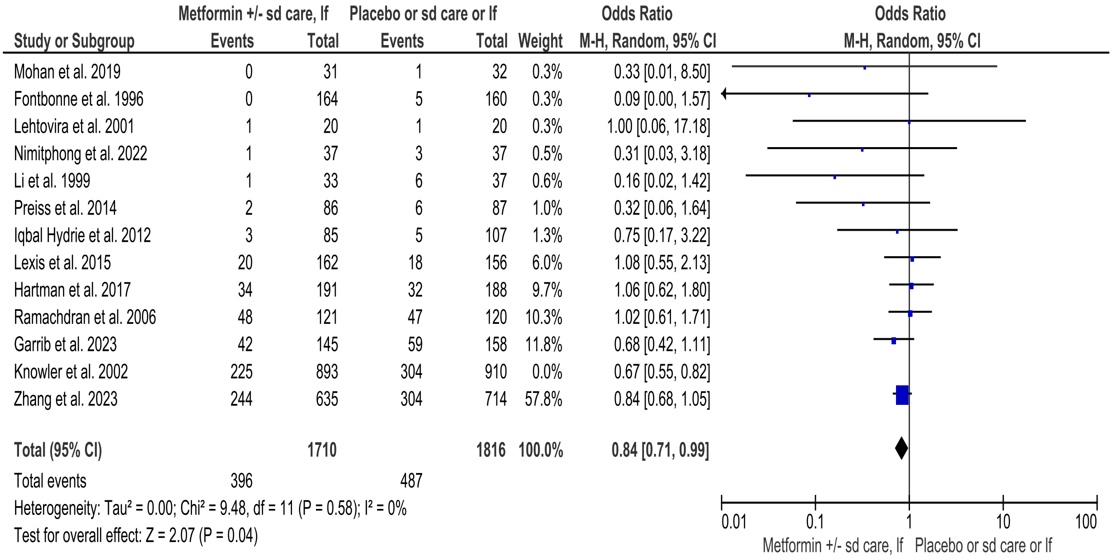

Sd, standard; lf, lifestyle; Events, number of participants with Type-2 Diabetes Mellitus; Total, number of participants at risk for Type-2 Diabetes Mellitus; Blue dots, weight of studies; Black blocks, 95% confidence interval of studies; Diamonds, estimates with 95% confidence interval.

Supplemental Figure S19. Sensitivity Analysis for RCT with post-intervention in overall effectiveness of metformin.

	Metformin +/- sd care, lf		Placebo or sd care or lf			Odds Ratio
Study or Subgroup	Events	Total	Events	Total	Weight	M-H, Random, 95% CI
Mohan et al. 2019	0	31	1	32	0.2%	0.33 [0.01, 8.50]
Fontbonne et al. 1996	0	164	5	160	0.2%	0.09 [0.00, 1.57]
Lehtovira et al. 2001	1	20	1	20	0.2%	1.00 [0.06, 17.18]
Nimitphong et al. 2022	1	37	3	37	0.3%	0.31 [0.03, 3.18]
Li et al. 1999	1	33	6	37	0.4%	0.16 [0.02, 1.42]
Preiss et al. 2014	2	86	6	87	0.7%	0.32 [0.06, 1.64]
Iqbal Hydrie et al. 2012	3	85	5	107	0.8%	0.75 [0.17, 3.22]
Lexis et al. 2015	20	162	18	156	3.8%	1.08 [0.55, 2.13]
Hartman et al. 2017	34	191	32	188	0.0%	1.06 [0.62, 1.80]
Ramachdran et al. 2006	48	121	47	120	6.6%	1.02 [0.61, 1.71]
Garrib et al. 2023	42	145	59	158	7.6%	0.68 [0.42, 1.11]
Zhang et al. 2023	244	635	304	714	37.0%	0.84 [0.68, 1.05]
Knowler et al. 2002	225	893	304	910	42.2%	0.67 [0.55, 0.82]
**Total (95% CI)**		**2412**		**2538**	**100.0%**	**0.75 [0.66, 0.86]**
Total events	587		759			

Heterogeneity: Tau² = 0.00; Chi² = 10.75, df = 11 (P = 0.46); I² = 0%

Test for overall effect: Z = 4.19 (P < 0.0001)

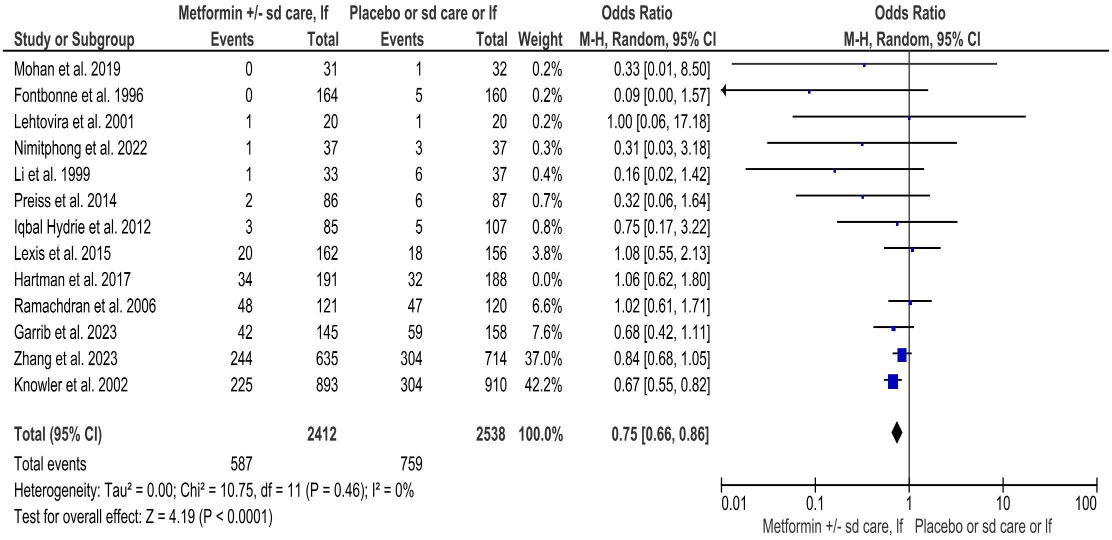

Sd, standard; lf, lifestyle; Events, number of participants with Type-2 Diabetes Mellitus; Total, number of participants at risk for Type-2 Diabetes Mellitus; Blue dots, weight of studies; Black blocks, 95% confidence interval of studies; Diamonds, estimates with 95% confidence interval.

Supplemental Figure S20. Sensitivity Analysis for RCTs with drop-out rate more than 10% in overall effectiveness of metformin.

Study or Subgroup	Metformin +/- sd care, lf Events	Metformin +/- sd care, lf Total	Placebo or sd care or lf Events	Placebo or sd care or lf Total	Weight	Odds Ratio M-H, Random, 95% CI
Mohan et al. 2019	0	31	1	32	0.2%	0.33 [0.01, 8.50]
Fontbonne et al. 1996	0	164	5	160	0.0%	0.09 [0.00, 1.57]
Lehtovira et al. 2001	1	20	1	20	0.2%	1.00 [0.06, 17.18]
Nimitphong et al. 2022	1	37	3	37	0.3%	0.31 [0.03, 3.18]
Li et al. 1999	1	33	6	37	0.0%	0.16 [0.02, 1.42]
Preiss et al. 2014	2	86	6	87	0.7%	0.32 [0.06, 1.64]
Iqbal Hydrie et al. 2012	3	85	5	107	0.0%	0.75 [0.17, 3.22]
Lexis et al. 2015	20	162	18	156	3.9%	1.08 [0.55, 2.13]
Hartman et al. 2017	34	191	32	188	6.4%	1.06 [0.62, 1.80]
Ramachdran et al. 2006	48	121	47	120	6.8%	1.02 [0.61, 1.71]
Garrib et al. 2023	42	145	59	158	0.0%	0.68 [0.42, 1.11]
Zhang et al. 2023	244	635	304	714	38.1%	0.84 [0.68, 1.05]
Knowler et al. 2002	225	893	304	910	43.3%	0.67 [0.55, 0.82]
**Total (95% CI)**		**2176**		**2264**	**100.0%**	**0.78 [0.68, 0.90]**
Total events	575		716			

Heterogeneity: Tau² = 0.00; Chi² = 7.73, df = 8 (P = 0.46); I² = 0%

Test for overall effect: Z = 3.55 (P = 0.0004)

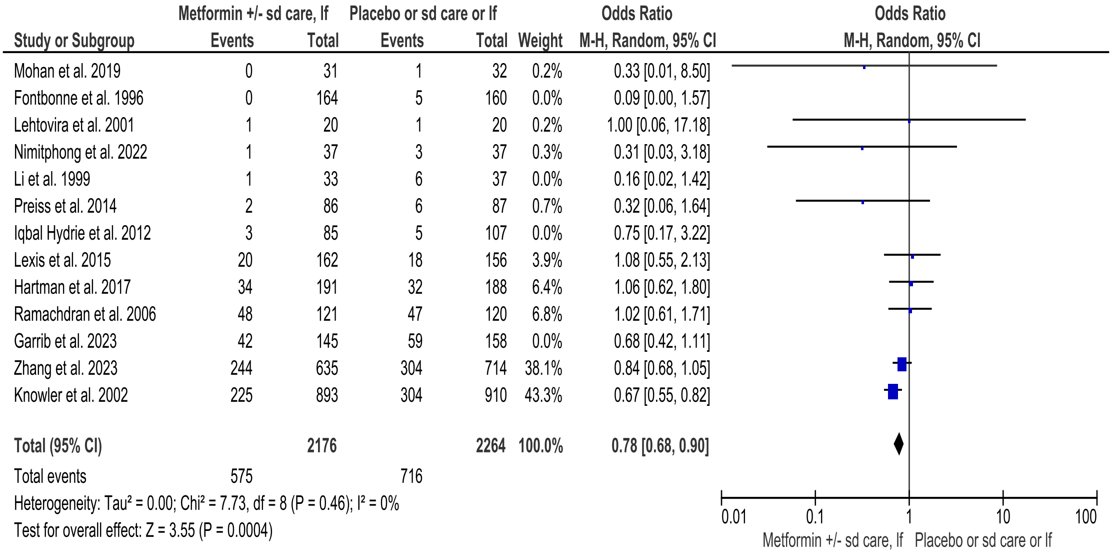

Events, number of participants with Type-2 Diabetes Mellitus; Total, number of participants at risk for Type-2 Diabetes Mellitus; Blue dots, weight of studies; Black blocks, 95% confidence interval of studies; Diamonds, estimates with 95% confidence interval.

Supplemental Figure S21. Sensitivity Analysis for RCT with the largest sample size in effectiveness of metformin and lifestyle interventions.

	Metformin & lifestyle		Standard care			Odds Ratio
Study or Subgroup	Events	Total	Events	Total	Weight	M-H, Random, 95% CI
Lu et al. 2011	0	46	5	41	10.6%	0.07 [0.00, 1.33]
Iqbal Hydrie et al. 2012	3	85	12	82	33.6%	0.21 [0.06, 0.79]
Ramachandran et al. 2006	48	121	73	133	0.0%	0.54 [0.33, 0.89]
Weber et al. 2016	23	104	24	75	55.8%	0.60 [0.31, 1.18]
**Total (95% CI)**		**235**		**198**	**100.0%**	**0.34 [0.12, 0.95]**
Total events	26		41			

Heterogeneity: $Tau^2 = 0.38$; $Chi^2 = 3.62$, df = 2 (P = 0.16); $I^2 = 45\%$

Test for overall effect: Z = 2.06 (P = 0.04)

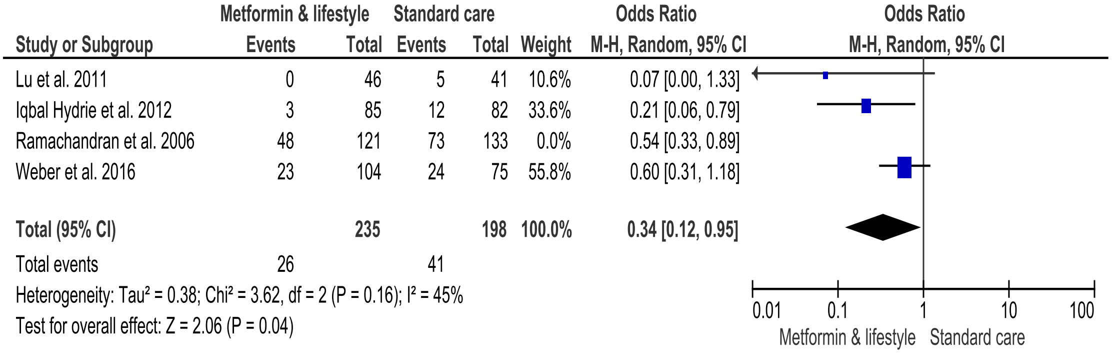

Events, number of participants with Type-2 Diabetes Mellitus; Total, number of participants at risk for Type-2 Diabetes Mellitus; Blue dots, weight of studies; Black blocks, 95% confidence interval of studies; Diamonds, estimates with 95% confidence interval.

Supplemental Figure S22. Sensitivity Analysis for RCT with post-intervention in effectiveness of metformin and lifestyle interventions.

	Metformin & lifestyle		Standard care			Odds Ratio
Study or Subgroup	Events	Total	Events	Total	Weight	M-H, Random, 95% CI
Lu et al. 2011	0	46	5	41	8.6%	0.07 [0.00, 1.33]
Iqbal Hydrie et al. 2012	3	85	12	82	29.7%	0.21 [0.06, 0.79]
Weber et al. 2016	23	104	24	75	0.0%	0.60 [0.31, 1.18]
Ramachandran et al. 2006	48	121	73	133	61.6%	0.54 [0.33, 0.89]
**Total (95% CI)**		**252**		**256**	**100.0%**	**0.34 [0.14, 0.86]**
Total events	51		90			

Heterogeneity: Tau² = 0.29; Chi² = 3.36, df = 2 (P = 0.19); I² = 40%

Test for overall effect: Z = 2.29 (P = 0.02)

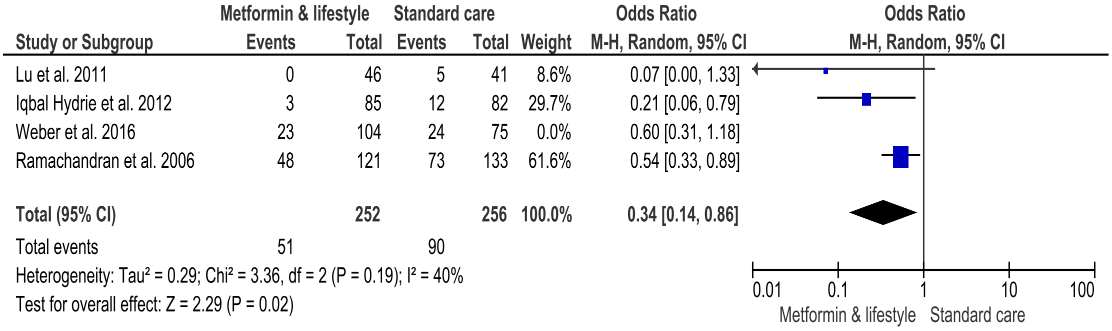

Events, number of participants with Type-2 Diabetes Mellitus; Total, number of participants at risk for Type-2 Diabetes Mellitus; Blue dots, weight of studies; Black blocks, 95% confidence interval of studies; Diamonds, estimates with 95% confidence interval.

Supplemental Figure S23. Sensitivity Analysis for RCTs with drop-out rate more than 10% in effectiveness of metformin and lifestyle interventions.

	Metformin & lifestyle		Standard care			Odds Ratio	Odds Ratio
**Study or Subgroup**	**Events**	**Total**	**Events**	**Total**	**Weight**	**M-H, Random, 95% CI**	**M-H, Random, 95% CI**
Lu et al. 2011	0	46	5	41	0.0%	0.07 [0.00, 1.33]	
Iqbal Hydrie et al. 2012	3	85	12	82	0.0%	0.21 [0.06, 0.79]	
Weber et al. 2016	23	104	24	75	35.7%	0.60 [0.31, 1.18]	
Ramachandran et al. 2006	48	121	73	133	64.3%	0.54 [0.33, 0.89]	
**Total (95% CI)**		**225**		**208**	**100.0%**	**0.56 [0.38, 0.84]**	
Total events	71		97				

Heterogeneity: Tau² = 0.00; Chi² = 0.07, df = 1 (P = 0.80); I² = 0%
Test for overall effect: Z = 2.82 (P = 0.005)

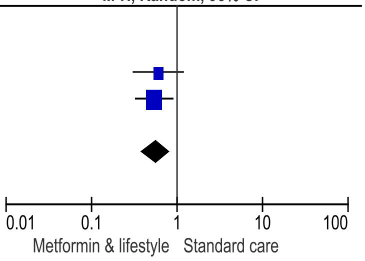

Events, number of participants with Type-2 Diabetes Mellitus; Total, number of participants at risk for Type-2 Diabetes Mellitus; Blue dots, weight of studies; Black blocks, 95% confidence interval of studies; Diamonds, estimates with 95% confidence interval.
